# Synthesis of Tricyclic and Tetracyclic Lactone Derivatives of Thieno[2,3-*b*]pyrazine or Thieno[2,3-*b*]quinoline: Preliminary Antitumor and Antiparasitic Activity Evaluation

**DOI:** 10.3390/molecules30091999

**Published:** 2025-04-30

**Authors:** Maria F. Martins, Francisco Ribeiro, Ana Borges, Ricardo C. Calhelha, Nuno Santarém, Anabela Cordeiro-da-Silva, Maria-João R. P. Queiroz

**Affiliations:** 1 Centro de Química, Universidade do Minho (CQ-UM), Campus de Gualtar, 4710-057 Braga, Portugal; mariamartinst19@gmail.com (M.F.M.); franciscoribeiroo1113@gmail.com (F.R.); 2 Centro de Investigação de Montanha (CIMO), Instituto Politécnico de Bragança, Campus de Santa Apolónia, 5300-253 Bragança, Portugal; ana.borges@ipb.pt (A.B.); calhelha@ipb.pt (R.C.C.); 3 Laboratório Associado para a Sustentabilidade e Tecnologia em Regiões de Montanha (SusTEC), Instituto Politécnico de Bragança, Campus de Santa Apolónia, 5300-253 Bragança, Portugal; 4 Host-Parasite Interactions, IBMC/I3S, Rua Alfredo Allen, 208, 4200-135 Porto, Portugal; santarem@ibmc.up.pt (N.S.); cordeiro@i3s.up.pt (A.C.-d.-S.); 5 Laboratório de Microbiologia, Departamento de Ciências Biológicas, Faculdade de Farmácia, Universidade do Porto, 4050-313 Porto, Portugal

**Keywords:** C-H activation, lactonization, metal-catalyzed cycloaddition, 2*H*-pyrones, Sonogashira coupling, thieno[2,3-*b*]pyrazines, thieno[2,3-*b*]quinolines

## Abstract

Tricyclic and tetracyclic lactone derivatives of thieno[2,3-*b*]pyrazine or thieno[2,3-*b*]quinoline, and 2*H*-pyrones were prepared using different methodologies. Pd/Cu-catalyzed Sonogashira coupling using Et_3_N as a base, of methyl 7-bromothieno[2,3-*b*]pyrazine-6-carboxylate and (het)arylalkynes to yield the Sonogashira ester products, gave also the corresponding tricyclic lactones as minor products. However, the major products did not cyclize with TFA. Tricyclic lactones were then obtained by a tandem one-pot Sonogashira coupling and 6-*endo-dig* lactonization of 7-bromothieno[2,3-*b*]pyrazine-6-carboxylic acid with (het)arylalkynes, in good yields. Halogenated tricyclic lactones were synthesized by halocyclization using CuX and NXS. Tetracyclic lactones were synthesized through a Rh(III)-catalyzed formal [4+2] cycloaddition, between thieno[2,3-*b*]quinoline-2-carboxylic acid and internal alkynes, triggered by C-H activation, with the carboxylic group acting as a directing group. Using the SRB assay, the antitumor activity of both Sonogashira products and lactones was evaluated across five human cancer cell lines (CaCo-2, MCF-7, AGS, HeLa, NCI-H460). The best-performing compound was a Sonogashira product showing a GI_50_ < 10 µM in all tumor cell lines and low toxicity in PLP2 cells. Additionally, antiparasitic testing against *Trypanosoma brucei* and *Leishmania infantum* revealed some compounds with IC_50_ < 11 µM, though some level of cytotoxicity was observed in THP-1—derived macrophages.

## 1. Introduction

Thieno[2,3-*b*]pyrazine derivatives constitute a significant class of aromatic heterocyclic compounds, largely due to the important biological activities exhibited by some of their derivatives. The synthesis and biological activities of these compounds have been predominantly reported in patents as inhibitors of the serine/threonine kinase B-Raf, which plays a role in the signal transduction pathways regulating cell growth and proliferation [1], as inhibitors of cancer cell growth and proliferation of different human tumor cell lines at low concentrations, as well as inducing apoptosis [2], as selective inhibitors of interleukin-1 receptor-associated kinase 4 (IRAK4) [3], or, as inhibitors of ubiquitin-proteasome system components, ubiquitin specific peptidase 28 (USP28) and/or ubiquitin specific peptidase 25 (USP25), that are involved in the treatment of cancer and inflammatory, autoimmune, and infectious diseases [4].

Thieno[2,3-*b*]quinolines have also attracted significant attention in medicinal chemistry due to their diverse biological activities [5]. Some derivatives have been described as selective inhibitors of the interaction between protein kinase C isoenzyme PKCε and the receptor for activated C-kinase 2 (RACK2) [6], as antioxidant and anti-inflammatory agents [7], or, as epidermal growth factor receptor (EGFR) inhibitors of tyrosine kinase domain (TKIs) [8].

2*H*-pyran-2-ones and their benzo derivatives, isocoumarins, are significant components of various lactone-based natural products that exhibit a broad variety of biological activities, including antifungal and antimicrobial properties [9,10].

**Scheme 1:**
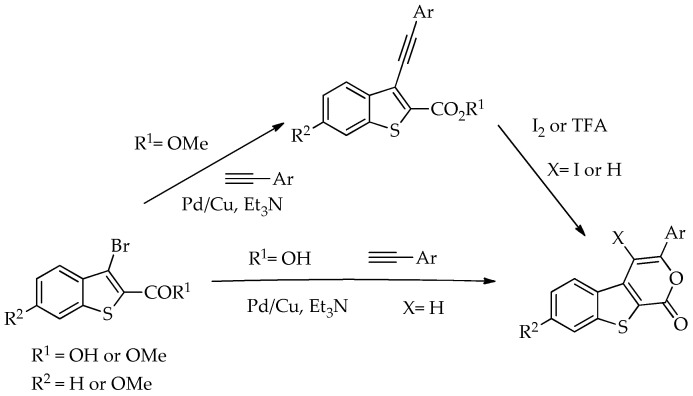
Synthesis of tricyclic lactones derivatives of benzo[*b*]thiophene [11].

In 2009, some of us reported the synthesis of 3-(aryl)benzothieno[2,3-*c*]pyran-1-ones. These compounds were synthesized via Pd/Cu-catalyzed Sonogashira coupling reactions of methyl 3-bromobenzo[*b*]thiophene-2-carboxylates with aryl alkynes followed by an electrophilic 6-*endo-dig* cyclization to give the aryl substituted pyranone ring iodinated or not using I_2_ or TFA. In the same study, the corresponding tricyclic lactones were also synthesized through a tandem one-pot reaction, which involved Pd/Cu-catalyzed Sonogashira coupling between 3-bromobenzo[*b*]thiophene-2-carboxylic acid and arylalkynes, followed by 6-*endo-dig* cyclization (Scheme 1). All the compounds prepared including the Sonogashira ester products were submitted to antitumor evaluation in tumor cell lines and some of them showed to be promising [11].

**Scheme 2:**
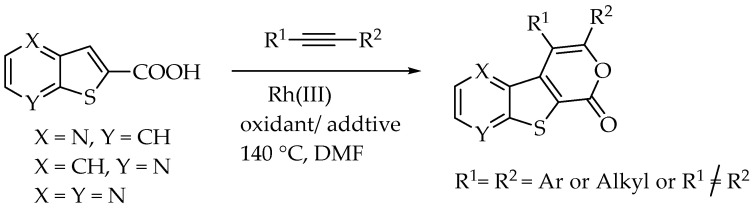
Synthesis of tricyclic lactones derivatives of thienopyridines and thieno[2,3-*b*]pyrazine [12].

More recently, we have focused on synthesizing tricyclic lactones that are disubstituted in the pyranone ring. This process involves a Rh(III)-catalyzed formal [4+2] cycloaddition between thienopyridine or thieno[2,3-*b*]pyrazine carboxylic acids and internal alkynes, initiated by C-H activation (Scheme 2) [12].

Herein, we report the synthesis of fused 2*H*-pyran-2-ones with thieno[2,3-*b*]pyrazine (tricyclic lactones) or thieno[2,3-*b*]quinoline (tetracyclic lactones) through the thiophene ring, using different methodologies. The resulting lactones could possess potential biological properties. The work initially describes the synthesis of Sonogashira coupling products from methyl 7-bromothieno[2,3-*b*]pyrazine-6-carboxylate and terminal (hetero)alkynes, as well as the formation of tricyclic lactones as minor products through 6-*endo-dig* lactonization of the coupling products. The Sonogashira ester products were prepared for cyclization into (halo)tricyclic lactones. The tricyclic lactones were also obtained in better yields in a tandem one-pot reaction of Sonogashira coupling followed by 6-*endo-dig* lactonization, by reaction of 7-bromothieno[2,3-*b*]pyrazine-6-carboxylic acid with terminal alkynes.

Furthermore, disubstituted tetracyclic lactones were prepared by Rh(III)-catalyzed formal [4+2] cycloaddition between thieno[2,3-*b*]quinoline-2-carboxylic acid and internal alkynes, triggered by C-H activation.

The synthesized compounds are new and were fully characterized. Furthermore, they were evaluated for their potential antitumor and antiparasitic activities.

## 2. Results and Discussion

### 2.1. Synthesis of Thieno[2,3-b]pyrazine Derivatives by Pd/Cu-Catalyzed Sonogashira Cross-Coupling Followed by Intramolecular Cyclization to Tricyclic Lactones

Sonogashira coupling products are usually precursors of molecules used as pharmaceuticals and/or organic materials [13,14]. Additionally, they are commonly employed in the synthesis of heterocycles through intramolecular cyclization [15].

#### 2.1.1. Pd/Cu-Catalyzed Sonogashira Cross-Coupling

The Sonogashira reaction for the coupling of methyl 7-bromothieno[2,3-*b*]pyrazine-6-carboxylate (**1a**) [16] with phenylacetylene to yield **2a** was performed using Pd/Cu and Et_3_N as a base in DMF, conditions previously used by us to reduce the formation of the alkyne dimer [11]. We compared these conditions with heterogeneous catalysis using Pd/C without copper, 2-Dicyclohexylphosphino-2′,4′,6′-triisopropylbiphenyl (XPhos) as a ligand [17], K_2_CO_3_ as a base, and eucalyptol as a solvent [18], which is a more sustainable approach. Under these conditions, compound **2a** was obtained in only a moderate yield (30%), and a major byproduct that was not identified was also formed (Table 1).

The best conditions (Table 1, entry 1) for the synthesis of the Sonogashira product **2a** were used for the coupling of compound **1a** with several terminal alkynes, to afford the corresponding Sonogashira coupling products **2a**–**h**, in good to high yields (50–95%), and the tricyclic lactones **3a**–**c**,**e**–**g** as minor products (5–12%), by regioselective 6-*endo-dig* cyclization [19], as depicted in Table 2. The tricyclic lactones may be formed via an electrophile-promoted nucleophilic cyclization (EPNC) with Et_3_N^+^H as source of H^+^ [20].

The highest yield was obtained for compound **2h** (95%, entry 8, Table 2) followed by compounds **2d** and **2g** (75%, entries 4 and 7, Table 2). Compound **2h** bears an electron-withdrawing group (EWG), while compound **2d** bears an electron-donating group (EDG) on the phenyl ring and compound **2g** contains a thiophene ring (electron rich). Compound **2a** was obtained in 65% (entry 1, Table 2), and compound **2b** bearing an amino group (EDG) was obtained in 60%, (entry 2, Table 2). The lowest yield (50%, entry 6, Table 2) was observed for compound **2f**, containing a pyridine ring (electron deficient), followed by compounds **2c** and **2e** (55%), bearing a F (weakly EWG) or a methyl group (EDG by inductive effect) (entries 3 and 5, Table 2). Overall, it is not possible to depict a definitive conclusion about the electronic nature of the substituents that enhance the reaction yield. However, among the heterocyclic rings, the electron-rich thiophene ring appears to favor the reaction more than the electron-poor pyridine ring.

For alkynes, Baldwin’s rules help in determining whether a five-membered (5-*exo-dig*) or six-membered (6-*endo-dig*) ring closure will be favored when an oxygen nucleophile attacks the alkyne, typically under EPNC conditions. These principles are essential in the design of efficient cyclization strategies in organic synthesis, as both 5-*exo-dig* and 6-*endo-dig* products can be obtained with regioselectivity, depending on the nature of the substituents and the nucleophile involved. Moreover, fused systems consisting of a six-membered ring following a five-membered ring, or vice versa, tend to be more stable [21]. Thus, the corresponding 6-*endo-dig* tricyclic lactones **3a**–**c**, **e**–**g** were formed regioselectively, as minor products.

To synthesize tricyclic lactones **3** via 6-*endo-dig* cyclization of the Sonogashira ester coupling products, compound **2a** was treated with TFA [11]. However, the cyclization did not proceed, possibly due to protonation of the nitrogens of the thienopyrazine. The strategy of using 7-bromothieno[2,3-*b*]pyrazine-6-carboxylic acid (**1b**) instead of **1a** as the starting material was used to obtain tricyclic lactones **3** in higher yields.

#### 2.1.2. Tandem One-Pot Reaction: Pd/Cu-Catalyzed Sonogashira Cross-Coupling Followed by 6-*Endo-dig* Cyclization to Tricyclic Lactones

Compound **1b** was reacted with various (hetero)arylalkynes via a tandem one-pot reaction, that involves a Sonogashira coupling followed by an electrophile-promoted nucleophilic cyclization [14,20]. The 6-*endo-dig* tricyclic lactones **3a**–**g** were isolated as the exclusive cyclization product in good yields (50–63%). Additionally, 7-[(hetero)arylethynyl]thieno[2,3-*b*]pyrazines (**4a**, **c**–**e**, **g**) were obtained as minor products in very low yields (3–12%) due to decarboxylation of the non-isolable intermediate Sonogashira products (Table 3).

**Scheme 3:**
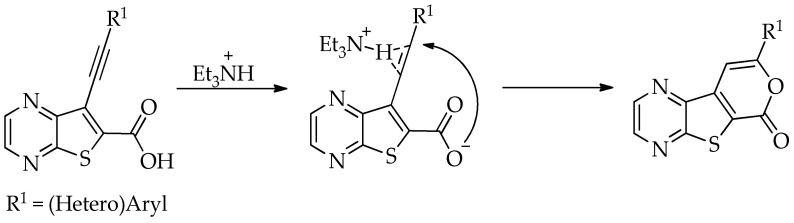
Plausible mechanism for electrophile-promoted nucleophilic lactonization using weak bases, adapted from Uchiyama et al. [20].

The highest yield was achieved for compound **3b** (63%, entry 2) derivative of a terminal alkyne bearing an amine on the phenyl ring. This was followed by compound **3a** (59%, entry 1, Table 3) derived from phenylacetylene, and compounds **3g** (54%, entry 7, Table 3) and **3d** (53%, entry 4, Table 3), using 3-ethynylthiophene and 4-ethynylanisole as terminal alkynes, respectively. The lowest yields were observed for compounds **3c**, **3e**, **3f** (51%-50%, entries 3, 5, 6 Table 3), using 1-ethynyl-4-fluorobenzene, 4-ethynyltoluene and 2-ethynylpyridine. The reaction of compound **1b** with 4-ethynylbenzonitrile gave a complex mixture of spots on TLC after 1 h of reaction. The ^1^H NMR spectrum of the crude mixture showed numerous signals that were difficult to assign to the expected 6-*endo-dig* lactone, so we did not proceed with purification. The presence of an EWG in the phenyl ring may reduce the availability of the triple bond for polarization, in contrast to the presence of EDGs, especially the amino group, which may enhance that polarization, as suggested by the following proposed mechanism. The EPNC may occur after deprotonation of the carboxylic group of the intermediate Sonogashira product by Et_3_N. The Et_3_N^+^H generated can coordinate with the π-bond, allowing for subsequent nucleophilic attack by an internal nucleophile, as shown in Scheme 3 and suggested by Uchiyama et al. in intramolecular cyclization studies of alkynes with adjacent carboxylic acids promoted by weak bases [20].

Compounds **3a**–**g** exhibited a ^1^H NMR signal for the 9-H proton between 7.57 and 8.13 ppm when using DMSO-*d_6_* as the solvent, as shown in Table 4. The shifts (δ) are in the aromatic zone, indicating that 6-*endo-dig* lactones were formed.

In some experiments, the decarboxylated Sonogashira product intermediates **4a**, **4c**–**e**, and **4g** were also isolated. The ^13^C NMR spectra of these compounds revealed carbon signals corresponding to their structures, including those for the triple bond carbons (~82 ppm and ~93 ppm, in DMSO-*d*_6_). The HRMS (ESI) data for all compounds **4** also confirmed their structures (see Section 3.1.1).

#### 2.1.3. Halocyclization to 9-Halo Tricyclic Lactones

Efforts to synthesize 9-iodotricyclic lactones from the Sonogashira ester coupling product **2a** using I_2_ and ICl have so far been unsuccessful. Therefore, the 9-halo-8-phenyl-6*H*-pyrano[4′,3′:4,5]thieno[2,3-*b*]pyrazin-6-ones **5a** and **5b** were successfully synthesized in good yields (50–60%) (Table 5) through halocyclization, using CuX_2_ and NXS (X = Cl, entry 1; Br, entry 2) [10].

**Scheme 4:**
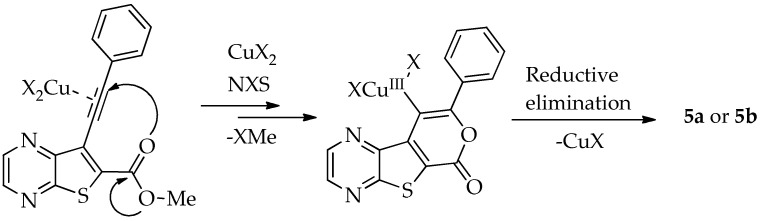
Adapted mechanism for halocyclization (X = Cl or Br) of compound **2a** from Miyata et al. [10].

In 2010, Miyata et al. proposed a reaction pathway for this type of chlorocyclization using *ο*-alkynyl amides, although the precise mechanism has not been fully elucidated. They proposed that CuCl_2_ activates the alkyne, facilitating nucleophilic addition of the carbonyl oxygen, which leads to the formation of a Cu(II) intermediate through a 6-*endo-dig* cyclization. Subsequent chlorination at the copper center by NCS generates a Cu(III) intermediate, which undergoes reductive elimination of CuCl to yield the chlorinated product [10]. An adapted mechanism for the halocyclization of compound **2a**, leading to the formation of compounds **5a** or **5b**, is presented in Scheme 4.

### 2.2. Synthesis of Tetracyclic Lactones Derived from Thieno[2,3-b]quinoline by Rh(III)-Catalyzed Formal [4+2] Cycloaddition, Triggered by C-H Activation

Synthetic methodologies based on C–H activation processes are among the most versatile and impactful approaches in contemporary organic chemistry. These techniques enable a rapid and atom-economical enhancement of molecular complexity starting from simple, unfunctionalized substrates rather than pre-functionalized precursors such as halogenated compounds [22,23]. However, given the abundance of various C–H bonds in organic molecules, achieving regioselective C–H activation and subsequent functionalization remains a formidable challenge. A highly promising approach to achieving high selectivity is utilizing existing functional groups within organic molecules as directing groups (DGs) [24,25]. For instance, functional groups like carboxylic acids can participate in subsequent cycloaddition reactions, acting as dynamic DGs [26,27]. Cycloaddition reactions are highly attractive due to their ability to simultaneously form two bonds and a ring in a single step. Extensive research has demonstrated that metal catalysts can effectively facilitate the cycloannulation of substrates that would otherwise remain unreactive [28].

Rh(III)-Catalyzed formal [4+2] cycloadditions between thieno[2,3-*b*]quinoline-2-carboxylic acid (**6**) and various internal alkynes, either symmetrical (aryl-aryl or alkyl-alkyl) or unsymmetrical (aryl-alkyl), were performed to synthesize 3,4-disubstituted-1*H*-pyrano[4′,3′:4,5]thieno[2,3-*b*]quinolin-1-ones (**7a**–**f**), in good to high yields (50–85%). The formation of these tetracyclic lactones was achieved using [RhCp*Cl_2_]_2_ as catalyst, Ag_2_CO_3_ as a base/oxidant and AgSbF_6_ as an additive, in dry DMF at 100 °C (Table 6). These conditions were selected based on our previous application of the same method to the thieno[2,3-*b*]pyridine system, where nitrogen and sulfur occupy the same relative positions [12]. Unlike the earlier experiments conducted at 140 °C, these reactions were carried out at 100 °C in dry DMF due to the high level of decarboxylation observed in the thieno[2,3-*b*]quinoline system (**6**) at 120 and 140 °C.

The highest yield was obtained for compound **7b** (85%, entry 2, Table 6), using 5-decyne as the internal alkyne. This was followed by the dibrominated compound **7c** (75%, entry 3, Table 6) and compound **7f** (70%, entry 6, Table 6), the latter obtained regioselectively from the unsymmetrical 1-phenyl-1-propyne. The regioisomer **7f** was assigned using nOe showing a 10% enhancement of the 5-H signal at 9.49 ppm upon saturation of the methyl group signal at 2.71 ppm (see Supplementary Materials). The lowest yield was observed for compound **7e** (50%, entry 5), which was synthesized using 1,2-bis(4-(trifluoromethyl)phenyl)ethyne. This reduced yield is likely attributed to the EWGs on the phenyl rings. Meanwhile, using 1,2-bis(4-methoxyphenyl)ethyne as the internal alkyne resulted in the formation of compound **7d** in 61% yield (entry 4), despite the presence of EDGs on the phenyl rings.

Table 7 presents the chemical shifts (δ) of the 5-H signal and 5-CH in ^1^H NMR and ^13^C NMR spectra, respectively, for all compounds **7**, recorded in DMSO-*d_6_* at 80 °C or 100 °C. The assignments were made based on HSQC ^1^H-^13^C 2D correlations, which enabled unambiguous identification of the 5-H and 5-CH signals (see Supplementary Materials). The lower δ values for the 5-H signal in compounds bearing aryl substituents can be attributed to the shielding cone generated by the aromatic ring. In contrast, compounds **7b** and **7f**, which feature alkyl substituents, exhibit the 5-H signal at higher δ values, indicating a more deshielded environment due to the absence of aromatic ring anisotropy—particularly in **7f**, where the spatial proximity of a methyl group likely contributes to this effect. A similar trend is observed for the 5-CH carbon signals. The structures of all compounds **7** were further corroborated by HRMS (ESI) data (see Section 3.1.2).

The use of eucalyptol instead of DMF, using diphenylacetylene or 5-decyne in the same conditions gave only traces of the corresponding tetracyclic lactones **7a** and **7b**.

**Scheme 5:**
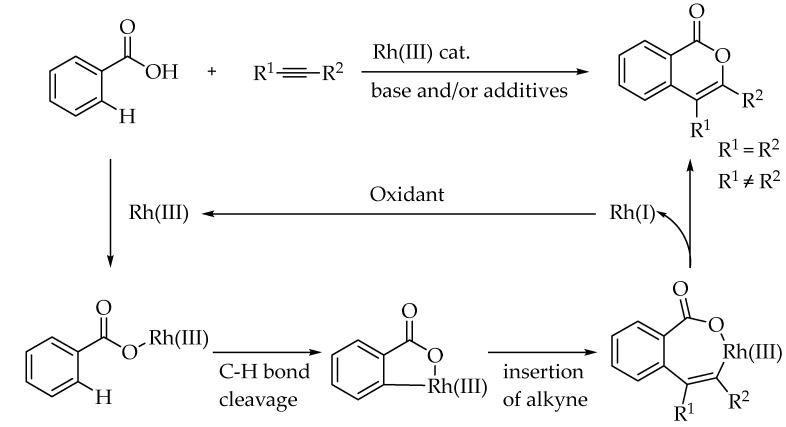
General mechanism for rhodium (III)-catalyzed formal [4+2] cycloaddition for benzoic acid adapted from You et al. [29].

The reaction mechanism begins with the C-H activation, facilitated by the proximity of the bond to the metal center, typically guided by a directing group. Concurrently, a base coordinated to the metal center promotes deprotonation of the C-H bond in a concerted process, resulting in the formation of the C-metal bond. This step is followed by the insertion of the internal alkyne. Subsequently, reductive elimination of the metal takes place, yielding the cycloannulation product. An external oxidant regenerates the active Rh(III) catalyst from Rh(I), enabling the catalytic cycle to continue. Scheme 5 illustrates the general mechanism proposed by You et al. for Rh(III)-catalyzed C-H activation and cycloaddition, using a carboxylic acid as a transient directing group and internal alkynes [29].

This reaction enabled the synthesis of tetracyclic lactone derivatives of thieno[2,3-*b*]quinolines through an atom-economical C-H activation methodology, facilitated by a carboxylic group that participates in the subsequent reaction. Using Rh(III), a formal [4+2] cycloaddition took place.

An attempt to carry out this reaction under the same conditions, but using the terminal alkyne phenylacetylene, resulted only in the formation of the alkyne dimer, rather than the corresponding monosubstituted tetracyclic lactone.

### 2.3. Cell Growth Inhibitory Effect of Compounds **2**, **3**, **5** and **7** on CaCo-2, MCF-7, AGS, HeLa, NCI-H460 Cell Lines and on a Non-Tumor Cell Line (PLP2)

The antitumor potential of the Sonogashira coupling products **2**, tricyclic lactones **3** and **5**, and tetracyclic lactones **7** was evaluated using the sulforhodamine B (SRB) assay [16,30] against five human tumor cell lines: colorectal adenocarcinoma (CaCo-2), breast adenocarcinoma (MCF-7), gastric adenocarcinoma (AGS), cervical carcinoma (HeLa), and non-small cell lung cancer (NCI-H460). A primary pig liver cell culture (PLP2) was used to assess the toxicity of compounds. Doxorubicin served as the positive control and the results are expressed as GI_50_ values (µM), as shown in Table 8.

All the compounds tested against these human tumor cell lines are not very promising, as their GI_50_ values are very much higher than 10 μM. Compound **2f**, as highlighted in Table 8, is the only promising antitumor agent within all series, showing activity against all tested human cell lines (GI_50_ < 10 μM) without significant toxicity in PLP2 at their GI_50_ concentrations. Notably, it exhibited higher activity against the Caco-2 and MCF-7 cell lines (GI_50_ < 5 μM) and warrants further investigation.

### 2.4. Cell Growth Inhibitory Effect of Compounds **2c**–**g**, **3a**, **5a** and **7** on T. brucei and L. infantum Promastigotes and THP-1-Derived Macrophage Cell Line as a Toxicity Model

The antiparasitic activity of compounds **2c**–**g**, **3a**, **5a** and **7** was evaluated at a single concentration of 20 μM, against *Trypanosoma brucei* (*T. brucei*) and *Leishmania infantum (L. infantum)* promastigotes. The results are shown in Table 9 together with the cytotoxicity data (CC_50_) in THP-1–derived macrophages cells [31]. Compounds exhibiting more than 50% inhibition undergo further testing, where dose-response curves were conducted to determine their IC_50_ values.

For *T. brucei*, two compounds, **2f** and **5a**, exhibited IC_50_ at low μM range, however, both displayed some level of toxicity as highlighted in Table 9. None of the compounds tested demonstrated activity against *L. infantum* promastigotes below 10 μM, which is conventionally used to define anti-Leishmanial hits.

## 3. Materials and Methods

### 3.1. Chemistry

The reactions were monitored by thin layer chromatography (TLC). The melting points were determined on a Stuart MP3 melting point apparatus and are uncorrected. Ether refers to diethyl ether and petroleum ether refers to petroleum ether 40–60 °C. ^1^H and ^13^C NMR were measured on a Bruker Avance III 400 (Bruker, Bremen, Germany), 400 MHz for ^1^H and 100.6 MHz for ^13^C, and using TopSpin 2.1 software. DEPT θ 135° was used to differentiate the type of carbons and ^1^H-^13^C bidimensional correlations, HSQC and HMBC, to identify some signals. The ^19^F NMR spectra were performed in a Bruker Avance III 300 (Bruker, Bremen, Germany) at 282.85 MHz. HRMS (ESI) results were obtained at the CACTI University of Vigo service, using a mass spectrometer Bruker FTMS SolariX XR. HPLC to determine the purity of compounds **3b**–**e** was performed using reverse-phase chromatography on a Jasco (Tokyo, Japan) PU-980/UV-975 system with a LiChrospher 100 RP-18 (5 µm) column. The mobile phase for these compounds consisted of acetonitrile (MeCN)/H_2_O in a 60:40 (*v*/*v*) ratio, with 0.1% trifluoroacetic acid (TFA) as an additive. For compound **7e**, the mobile phase was MeCN/H_2_O in a 90:10 (*v*/*v*) ratio. Detection was carried out at a λ = 350 nm.

#### 3.1.1. Synthesis of Thieno[2,3-*b*]pyrazine Derivatives by Pd/Cu-Catalyzed Sonogashira Cross-Coupling and Intramolecular Cyclization to Tricyclic Lactones

*7-bromothieno[2,3-b]pyrazine-6-carboxylic acid* (**1b**): Compound **1a** (0.617 g, 2.26 mmol) was dissolved in THF/MeOH/H_2_O (6:1:1, 60 mL) and LiOH (0.203 g, 8.47 mmol) was added and the mixture was stirred for 3 h at rt, monitored by TLC. After partial removal of the solvents, the mixture was acidified using HCl_conc_ till pH = 5 and a precipitate came out. This was filtered under vacuum, washed with H_2_O and dried in the oven at 50 °C for several hours. Compound **1b** was obtained as a white solid (0.530 g, 90%). ^1^H NMR (400 MHz, DMSO-*d_6_*): δ = 8.85 (1 H, d, *J* = 2.0 Hz, HetAr-H), 8.95 (1 H, d, *J* = 2.0 Hz, HetAr-H) ppm. HRMS (ESI) [M + H]^+^, *m*/*z* Calculated for C_7_H_4_^79^BrN_2_O_2_S: 258.9172, found 258.9169. Calculated for C_7_H_4_^81^BrN_2_O_2_S: 260.9151, found 260.9146.

##### General Procedure for the Synthesis of Sonogashira Cross-Coupling Products (**2a**–**g**) 

In a vacuum-dried Schlenk tube with dry DMF (2 mL), compound **1a** (1.0 equiv.) and PdCl_2_(PPh_3_)_2_ (5 mol%) were added and stirred, under Ar. In a small Schlenk flask with dry DMF (2 mL), the alkyne (1.1 equiv.), Et_3_N (3.0 equiv.) and CuI (5 mol%) were added and stirred, under Ar. This mixture was added to the one in the Schlenk tube, and it was left stirring for 5 min at rt. After this, the final mixture was heated at 100 °C with stirring under Ar for 3–4 h, monitoring by TLC. After cooling, water (5 mL) was added and a precipitate came out. This was filtered under vacuum and submitted to column chromatography using solvent gradients of ether/petroleum ether increasing 10% each time, to give compounds **2** and **3** as minor products, unless stated otherwise.

*Methyl 7-(phenylethynyl)thieno[2,3-b]pyrazine-6-carboxylate* (**2a**) *and 8-phenyl-6H-pyrano[4′,3′:4,5]thieno[2,3-b]pyrazin-6-one* (**3a**): Compound **1a** (0.100 g, 0.366 mmol), phenylacetylene (0.0460 g, 0.0490 mL, 0.403 mmol) heating for 3.5 h. Column chromatography using a solvent gradient from 30 to 50% ether/petroleum ether gave compound **2a** as a light yellow solid (0.0700 g, 65%), m.p. 145–147 °C. ^1^H NMR (400 MHz, DMSO-*d_6_*): δ = 3.98 (3 H, s, OCH_3_), 7.48–7.52 (3 H, m, Ar-H), 7.63–7.65 (2 H, m, Ar-H), 8.86 (1 H, d, *J* = 2.4 Hz, HetAr-H), 8.97 (1 H, d, *J* = 2.4 Hz, HetAr-H) ppm. ^13^C NMR (100.6 MHz, DMSO-d_6_): δ = 53.3 (OCH_3_), 81.8 (C), 99.6 (C), 121.5 (C), 121.7 (C), 129.0 (2 × ArCH), 129.8 (4′-CH), 131.7 (2 × Ar-CH), 136.3 (C), 144.3 (HetAr-CH), 144.7 (HetAr-CH), 147.8 (C), 154.3 (C), 161.1 (C=O) ppm. HRMS (ESI) [M + H]^+^, *m*/*z* Calculated for C_16_H_11_N_2_O_2_S: 295.0536; found: 295.0540.

A more polar compound, **3a**, was also isolated as light orange solid (0.0120 g, 12%). ^1^H NMR (400 MHz, DMSO-d_6_): δ = 7.53–7.58 (3 H, m, Ar-H), 7.93 (1 H, s, 9-H), 8.04–8.06 (2 H, m, Ar-H), 8.94 (1 H, d, *J* = 2.4 Hz, HetAr-H), 9.03 (1 H, d, *J* = 2.4 Hz, HetAr-H) ppm. This compound was fully characterized when obtained as a major product from the carboxylic acid **1b**.

*Methyl 7-[(4-aminophenyl)ethynyl]thieno[2,3-b]pyrazine-6-carboxylate* (**2b**) *and 8-(4-aminophenyl)-6H-pyrano[4′,3′:4,5]thieno[2,3-b]pyrazin-6-one* (**3b**): Compound **1a** (0.100 g, 0.366 mmol), 4-ethynylaniline (0.0490 g, 0.403 mmol) heating for 3 h. Column chromatography using a solvent gradient from 20 to 60% ethyl acetate/petroleum ether gave compound **2b** as an orange solid (0.0683 g, 60%), m.p. 203–204 °C. ^1^H NMR (400 MHz, DMSO-*d_6_*): δ = 3.97 (3 H, s, OCH_3_), 5.80 (2 H, s, NH_2_), 6.61 (2 H, d, *J* = 8.8 Hz, 3′ and 5′-H), 7.31 (2 H, d, *J* = 8.8 Hz, 2′ and 6′-H), 8.84 (1 H, d, *J* = 2.4 Hz, HetAr-H), 8.95 (1 H, d, *J* = 2.4 Hz, HetAr-H) ppm. ^13^C NMR (100.6 MHz, DMSO-*d_6_*): δ = 53.0 (OCH_3_), 80.4 (C), 103.3 (C), 107.1 (C), 113.7 (3′ and 5′-CH), 122.9 (C), 133.2 (C), 133.4 (2′ and 6′-CH), 144.0 (HetAr-CH), 144.6 (HetAr-CH), 147.8 (C), 150.6 (C), 154.4 (C), 161.4 (C=O) ppm. HRMS (ESI) [M + H]^+^, *m*/*z* Calculated for C_16_H_12_N_3_O_2_S: 310.0645; found: 310.0645.

A more polar compound, **3b**, was also isolated as a dark orange solid (0.00500 g, 5%). ^1^H NMR (400 MHz, DMSO-*d_6_*): δ = 5.89 (2 H, s, NH_2_), 6.66 (2 H, d, *J* = 8.8 Hz, 3′ and 5′-H), 7.57 (1 H, s, 9-H), 7.72 (2 H, d, *J* = 8.8 Hz, 2′ and 6′-H), 8.91 (1 H, d, *J* = 2.4 Hz, HetAr-H), 8.99 (1 H, d, *J* = 2.4 Hz, HetAr-H) ppm. This compound was fully characterized when obtained as a major product from the carboxylic acid **1b**.

*Methyl 7-[(4-fluorophenyl)ethynyl]-thieno[2,3-b]pyrazine-6-carboxylate* (**2c**) *and 8-(4-fluorophenyl)-6H-pyrano[4′,3′:4,5]thieno[2,3-b]pyrazin-6-one* (**3c**): Compound **1a** (0.0760 g, 0.278 mmol), 1-ethynyl-4-fluorobenzene (0.0370 g, 0.306 mmol) heating for 4 h. Column chromatography using a solvent gradient from 30 to 50% ether/petroleum ether gave compound **2c** as a white solid (0.0473 g, 55%), m.p. 175–176 °C. ^1^H NMR (400 MHz, DMSO-*d_6_*): δ = 3.99 (3 H, s, OCH_3_), 7.32–7.38 (2 H, m, 3′ and 5′-H), 7.68–7.73 (2 H, m, 2′ and 6′-H), 8.88 (1 H, d, *J* = 2.0 Hz, HetAr-H), 8.98 (1 H, d, *J* = 2.0 Hz, HetAr-H) ppm. ^13^C NMR (100.6 MHz, DMSO-d_6_): δ = 53.3 (OCH_3_), 81.6 (C), 98.5 (C), 116.4 (d, *J* = 22.1 Hz, 3′ and 5′-CH), 118.1 (d, *J* = 3.0 Hz, 1′-C), 121.3 (C), 134.2 (d, *J* = 9.1 Hz, 2′ and 6′-CH), 136.4 (C), 144.3 (HetAr-CH), 144.7 (HetAr-CH), 147.7 (C), 154.3 (C), 161.0 (C=O), 162.6 (d, *J* = 248.5 Hz, CF) ppm. ^19^F NMR (282.85 MHz, DMSO-*d_6_*) δ = −104.9 ppm. HRMS (ESI) [M + H]^+^, *m*/*z* Calculated for C_16_H_10_FN_2_O_2_S: 313.0441; found: 313.0441.

A more polar compound, **3c**, was also isolated as beige solid (0.00900 g, 12%). ^1^H NMR (400 MHz, DMSO-*d_6_*): δ = 7.37–7.42 (2 H, m, 3′ and 5′-H), 7.96 (1 H, s, 9-H), 8.12-8.15 (2 H, m, 2′ and 6′-H), 8.95 (1 H, d, *J* = 2.4 Hz, HetAr-H), 9.04 (1 H, d, *J* = 2.4 Hz, HetAr-H) ppm. This compound was fully characterized when obtained as a major product from the carboxylic acid **1b**.

*Methyl 7-[(4-methoxyphenyl)ethynyl]thieno[2,3-b]pyrazine-6-carboxylate* (**2d**): Compound **1a** (0.0800 g, 0.290 mmol), 4-ethynylanisole (0.0420 g, 0.0410 mL, 0.320 mmol) heating for 4 h. Column chromatography using a solvent gradient from 10 to 40% ether/petroleum ether gave compound **2d** as a yellow solid (0.0710 g, 75%), m.p. 150–152 °C. ^1^H NMR (400 MHz, DMSO-*d_6_*): δ = 3.82 (3 H, s, 4′-OCH_3_), 3.98 (3 H, s, OCH_3_), 7.04–7.07 (2 H, d, *J* = 8.8 Hz, 3′ and 5′-H), 7.58–7.60 (2 H, d, *J* = 8.8 Hz, 2′ and 6′-H), 8.86–8.87 (1 H, d, *J* = 2.4 Hz, HetAr-H), 8.97 (1 H, d, *J* = 2.4 Hz, HetAr-H) ppm. ^13^C NMR (100.6 MHz, DMSO-*d_6_*): δ = 53.2 (OCH_3_), 55.4 (4′-OCH_3_), 80.9 (C), 100.3 (C), 113.5 (C), 114.7 (3′ and 5′-CH), 122.0 (C), 133.5 (2′ and 6′-CH), 135.2 (C), 144.2 (HetAr-CH), 144.7 (HetAr-CH), 147.8 (C), 154.3 (C), 160.4 (C), 161.1 (C=O) ppm. HRMS (ESI) [M + H]^+^, *m*/*z* Calculated for C_17_H_13_N_2_O_3_S: 325.0641; found: 325.0642.

*Methyl 7-(p-tolylethynyl)thieno[2,3-b]pyrazine-6-carboxylate* (**2e**) *and 8-(p-Tolyl)-6H-pyrano[4′,3′:4,5]thieno[2,3-b]pyrazin-6-one* (**3e**): Compound **1a** (0.100 g, 0.366 mmol), 4-ethynyltoluene (0.0470 g, 0.403 mmol) heating for 4 h. Column chromatography using a solvent gradient from 10 to 20% ether/petroleum ether gave compound **2e** as a yellow solid (0.0616 g, 55%) m.p. 146–147 °C. ^1^H NMR (400 MHz, DMSO-*d_6_*): δ = 2.37 (3 H, s, CH_3_), 3.99 (3 H, s, OCH_3_), 7.31 (2 H, d, *J* = 8.0 Hz, 3 ’and 5′-H), 7.53 (2 H, d, *J* = 8.0 Hz, 2′ and 6′-H), 8.87 (1 H, d, *J* = 2.0 Hz, HetAr-H), 8.98 (1 H, d, *J* = 2.0 Hz, HetAr-H) ppm. ^13^C NMR (100.6 MHz, DMSO-*d_6_*): δ = 21.2 (CH_3_), 53.2 (OCH_3_), 81.4 (C), 100.0 (C), 118.6 (C), 121.7 (C), 129.6 (3′ and 5′-CH), 131.7 (2′ and 6′-CH), 135.9 (C), 139.8 (C), 144.2 (HetAr-CH), 144.7 (HetAr-CH), 147.8 (C), 154.3 (C), 161.1 (C=O) ppm. HRMS (ESI) [M + H]^+^, *m*/*z* Calculated for C_17_H_13_N_2_O_2_S: 309.0692; found: 309.0696.

A more polar compound, **3e**, was also isolated as yellow solid (0.0110 g, 10%). ^1^H NMR (400 MHz, DMSO-*d_6_*): δ = 2.39 (3 H, s, CH_3_), 7.36 (2 H, d, *J* = 8.0 Hz, 3′ and 5′-H), 7.78 (1 H, s, 9-H), 7.90 (2 H, d, *J* = 8.0 Hz, 2′ and 6′-H), 8.90 (1 H, d, *J* = 2.4 Hz, HetAr-H), 8.99 (1H, d, *J* = 2.4 Hz, HetAr-H) ppm. This compound was fully characterized when obtained as a major product from the carboxylic acid **1b**.

*Methyl 7-(pyridin-2-ylethynyl)thieno[2,3-b]pyrazine-6-carboxylate* (**2f**) *and 8-(Pyridin-2-yl)-6H-pyrano[4′,3′:4,5]thieno[2,3-b]pyrazin-6-one* (**3f**): Compound **1a** (0.0800 g, 0.290 mmol), 2-ethynylpyridine (0.0340 g, 0.0330 mL, 0.320 mmol) heating for 5 h. Column chromatography using a solvent gradient from 10 to 60% ethyl acetate/petroleum ether gave compound **2f** as a white solid (0.0420g, 50%), m.p. 183–185°C. ^1^H NMR (400 MHz, DMSO-*d_6_*): δ = 3.99 (3 H, s, OCH_3_), 7.48–7.51 (1 H, ddd, *J* = 7.6, 4.8 and 1.2 Hz, 5′-H), 7.72–7.74 (1 H, dt, *J* = 7.6, 1.2 and 0.8 Hz, 3′-H), 7.90–7.94 (1 H, app. td, J= 7.6 and 1.6 Hz, 4′-H), 8.67–8.69 (1 H, dq, *J* = 4.8, 1.6 and 0.8 Hz, 6′-H), 8.89 (1 H, d, *J* = 2.4 Hz, HetAr-H), 8.99 (1 H, d, *J* = 2.4 Hz, HetAr-H) ppm. ^13^C NMR (100.6 MHz, DMSO-*d_6_*): δ = 53.3 (OCH_3_), 80.4 (C), 98.4 (C), 120.6 (C), 124.3 (5′-CH), 128.0 (3′-CH), 137.0 (4′-CH), 137.7 (C), 141.7 (C), 144.4 (HetAr-CH), 144.8 (HetAr-CH), 147.9 (C), 150.5 (6′-CH), 154.2 (C), 160.9 (C=O) ppm. HRMS (ESI) [M + H]^+^, *m*/*z* Calculated for C_15_H_10_N_3_O_2_S: 296.0488; found: 296.0488.

A more apolar compound, **3f**, was also isolated as white solid (0.00800 g, 10%). ^1^H NMR (400 MHz, DMSO-*d_6_*): δ = 7.53–7.56 (1 H, m, 5′-H), 8.02–8.03 (2 H, m, 3′ and 4′-H), 8.13 (1 H, s, 9-H), 8.74–8.76 (1 H, m, 6′-H), 8.95 (1 H, d, *J* = 2.4 Hz, HetAr-H), 9.05 (1H, d, *J* = 2.4 Hz, HetAr-H) ppm. This compound was fully characterized when obtained as a major product from the carboxylic acid **1b**.

*Methyl 7-(thiophen-3-ylethynyl)thieno[2,3-b]pyrazine-6-carboxylate* (**2g**) *and 8-(thiophen-3-yl)-6H-pyrano[4′,3′:4,5]thieno[2,3-b]pyrazin-6-one* (**3g**): Compound **1a** (0.100 g, 0.366 mmol), 3-ethynylthiophene (0.0450 g, 0.0410 mL, 0.403 mmol) heating for 3.5 h. Column chromatography using a solvent gradient from 20 to 60% ether/petroleum ether gave compound **2g** as a dark orange solid (0.0840 g, 75%), m.p. 158–160 °C. ^1^H NMR (400 MHz, DMSO-*d_6_*): δ = 3.98 (3 H, s, OCH_3_), 7.33 (1 H, dd, *J* = 5.2 and 1.2 Hz, 4′-H), 7.72 (1 H, dd, *J* = 5.2 and 2.8 Hz, 5′-H), 8.05 (1 H, dd, *J* = 2.8 and 1.2 Hz, 2′-H), 8.87 (1 H, d, *J* = 2.4 Hz, HetAr-H), 8.96 (1 H, d, *J* = 2.4 Hz, HetAr-H) ppm. ^13^C NMR (100.6 MHz, DMSO-*d_6_*): δ = 53.2 (OCH_3_), 81.2 (C), 95.2 (C), 120.5 (C), 121.6 (C), 127.5 (5′-CH), 129.6 (4′-CH), 131.7 (2′-CH), 135.9 (C), 144.2 (HetAr-CH), 144.7 (HetAr-CH), 147.8 (C), 154.2 (C), 161.0 (C=O) ppm. HRMS (ESI) [M + H]^+^, *m*/*z* Calculated for C_14_H_9_N_2_O_2_S_2_: 301.0100; found: 301.0096.

A more polar compound, **3g**, was also isolated as a light yellow solid (0.00700 g, 7%). ^1^H NMR (400 MHz, DMSO-*d_6_*): δ = 7.73–7.75 (1 H, dd, *J* = 5.2 and 2.8 Hz, 5′-H), 7.81 (1 H, dd, *J* = 5.2 and 1.2 Hz, 4′-H), 7.84 (1 H, s, 9-H), 8.27 (1 H, dd, *J* = 2.8 and 1.2 Hz, 2′-H), 8.93 (1 H, d, *J* = 2.4 Hz, HetAr-H), 9.02 (1 H, d, *J* = 2.4 Hz, HetAr-H) ppm. This compound was fully characterized when obtained as a major product from the carboxylic acid **1b**.

*Methyl 7-[(4-cyanophenyl)ethynyl]thieno[2,3-b]pyrazine-6-carboxylate* (**2h**): Compound **1a** (0.050 g, 0.183 mmol), 4-ethynylbenzonitrile (0.0256 g, 0.201 mmol) heating for 4 h. After cooling, H_2_O was added and a precipitate came out. This was filtered under vacuum, dried in the oven overnight at 50 °C giving a light brown solid, that after some washes with dry ether gave compound **2h** as a beige solid (0.0555 g, 95%), m.p. 281–282 °C. ^1^H NMR (400 MHz, DMSO-*d_6_*, 60 °C): δ = 4.00 (3 H, s, OCH_3_), 7.80 (2 H, d, *J* = 8.0 Hz, 2′ and 6’-H), 7.92 (2 H, d, *J* = 8.0 Hz, 3′ and 5’-H), 8.86 (1 H, d, *J* = 2.0 Hz, HetAr-H), 8.96 (1 H, d, *J* = 2.0 Hz, HetAr-H) ppm. ^13^C NMR (100.6 MHz, DMSO-*d_6_*, 60 °C): δ = 52.9 (OCH_3_), 84.9 (C), 97.2 (C), 111.6 (C), 117.8 (C), 120.3 (C), 126.2 (C), 132.1 (2′ and 6’-CH), 132.4 (3′ and 5’-CH), 137.4 (C), 144.0 (HetAr-CH), 144.4 (HetAr-CH), 147.4 (C), 154.0 (C), 161.5 (C=O) ppm. HRMS (ESI) [M + H]^+^, *m*/*z* Calculated for C_17_H_10_N_3_O_2_S: 320.0489; found: 320.0492.

##### General Procedure for Tricyclic Lactones (**3a**–**g**) by One Pot Tandem Sonogashira Coupling and Intramolecular Cyclization 

In a vacuum-dried Schlenk tube with dry DMF/Et_3_N (2:1, 3 mL), compound **2b** (1.0 equiv.), PdCl_2_(PPh_3_)_2_ (10 mol%), CuI (20 mol%) and the alkyne (1.1 equiv.) were added and stirred, under Ar. After this, the mixture was heated at 100 °C with stirring under Ar for 1–2 h, monitoring by TLC. After cooling, water was added (10 mL) and the mixture was extracted with ethyl acetate (3 × 10 mL). The organic phase was washed successively with water (3 × 10 mL) and brine (2 × 10 mL), dried (MgSO_4_), filtered, and concentrated under reduced pressure. The resulting residue was submitted to column chromatography to give compounds **3** and **4** as minor products.

*8-Phenyl-6H-pyrano[4′,3′:4,5]thieno[2,3-b]pyrazin-6-one* (**3a**) *and 7-(phenylethynyl)thieno[2,3-b]pyrazine* (**4a**): Compound **1b** (0.100 g, 0.386 mmol), phenylacetylene (0.0430 g, 0.0466 mL, 0.425 mmol) and heating for 1 h. Column chromatography using a solvent gradient from 10 to 20% ether/petroleum ether gave compound **3a** as a yellow solid (0.0630 g, 59%), m.p. 202–204 °C. ^1^H NMR (400 MHz, DMSO-d_6_): δ = 7.53–7.58 (3 H, m, Ar-H), 7.93 (1 H, s, 9-H), 8.04–8.06 (2 H, m, Ar-H), 8.94 (1 H, d, *J* = 2.4 Hz, HetAr-H), 9.03 (1 H, d, *J* = 2.4 Hz, HetAr-H) ppm. ^13^C NMR (100.6 MHz, DMSO-d_6_): δ = 96.6 (9-CH), 122.9 (C), 125.5 (2 × Ar-CH), 129.2 (2 × Ar-CH), 130.8 (4′-CH), 130.9 (C), 141.5 (C), 143.8 (C), 143.9 (HetAr-CH), 145.6 (HetAr-CH), 157.5 (C), 157.6 (C), 158.1 (C) ppm. HRMS (ESI) [M + H]^+^, *m*/*z* Calculated for C_15_H_9_N_2_O_2_S: 281.0379; found: 281.0379.

A more apolar compound, **4a**, was also isolated as white solid (0.0110 g, 12%). ^1^H NMR (400 MHz, DMSO-d_6_): δ = 7.45–7.46 (3 H, m, Ar-H), 7.59–7.62 (2 H, m, Ar-H), 8.66 (1 H, s, 6-CH), 8.72 (1 H, d, *J* = 2.4 Hz, HetAr-H), 8.86 (1 H, d, *J* = 2.4 Hz, HetAr-H) ppm. ^13^C NMR (100.6 MHz, DMSO-d_6_): δ = 82.0 (C), 92.6 (C), 116.3 (C), 121.8 (C), 128.9 (2 × Ar-CH), 129.2 (4′-CH), 131.5 (Ar-CH), 136.4 (6-CH), 141.8 (HetAr-CH), 142.8 (HetAr-CH), 147.7 (C), 153.9 (C) ppm. HRMS (ESI) [M + H]^+^, *m*/*z* Calculated for C_14_H_9_N_2_S: 237.0481, found 237.0478.

*8-(4-Aminophenyl)-6H-pyrano[4′,3′:4,5]thieno[2,3-b]pyrazin-6-one* (**3b**): Compound **1b** (0.100 g, 0.386 mmol), 4-ethynylaniline (0.0410 g, 0.340 mmol) and heating for 2 h. Column chromatography using a solvent gradient from 20 to 50% ethyl acetate/petroleum ether gave compound **3b** as a dark orange solid (0.0570 g, 63%), m.p. 286–287 °C. ^1^H NMR (400 MHz, DMSO-*d_6_*): δ = 5.89 (2 H, s, NH_2_), 6.66 (2 H, d, *J* = 8.8 Hz, 3′ and 5′-H), 7.57 (1 H, s, 9-H), 7.72 (2 H, d, *J* = 8.8 Hz, 2′ and 6′-H), 8.91 (1 H, d, *J* = 2.4 Hz, HetAr-H), 8.99 (1 H, d, *J* = 2.4 Hz, HetAr-H) ppm. ^13^C NMR (100.6 MHz, DMSO-*d_6_*): δ = 92.6 (9-CH), 113.7 (3′ and 5′-CH), 117.5 (C), 119.3 (C), 127.1 (2′ and 6′-CH), 142.5 (C), 143.7 (HetAr-CH), 143.9 (C), 145.5 (HetAr-CH), 151.7 (C), 157.9 (C), 158.3 (C), 159.7 (C) ppm. Calculated for C_15_H_10_N_3_O_2_S: 296.0488; found: 296.0488. HPLC retention time = 4.02 min, **3b** purity is 100%.

*8-(4-Fluorophenyl)-6H-pyrano[4′,3′:4,5]thieno[2,3-b]pyrazin-6-one* (**3c**) *and 7-[(4-Fluorophenyl)ethynyl]thieno[2,3-b]pyrazine* (**4c**): Compound **1b** (0.100 g, 0.386 mmol), 1-ethynyl-4-fluorobenzene (0.0520 g, 0.425 mmol) and heating for 2 h. Column chromatography using a solvent gradient from 10 to 50% ether/petroleum ether gave compound **3c** as a yellow solid (0.0580 g, 35%), m.p. 269–271 °C. ^1^H NMR (400 MHz, DMSO-*d_6_*): δ = 7.37–7.42 (2 H, m, 3′ and 5′-H), 7.96 (1 H, s, 9-H), 8.12-8.15 (2 H, m, 2′ and 6′-H), 8.95 (1 H, d, *J* = 2.4 Hz, HetAr-H), 9.04 (1 H, d, *J* = 2.4 Hz, HetAr-H) ppm. ^13^C NMR (100.6 MHz, DMSO-*d_6_*): δ = 96.7 (9-CH), 116.4 (d, *J* = 22.1 Hz, 3′ and 5′-CH), 122.9 (C), 127.7 (d, *J* =3.0 Hz, 1′-C), 128.2 (d, *J* = 9.1 Hz, 2′ and 6′-CH), 141.6 (C), 143.9 (C), 144.1 (HetAr-CH), 145.8 (HetAr-CH), 156.9 (C), 157.6 (C), 158.3 (C), 163.6 (d, *J* = 250.5 Hz, CF) ppm. ^19^F NMR (282.85 MHz, DMSO-*d_6_*) δ = −105.5 ppm. HRMS (ESI) [M + H]^+^, *m*/*z* Calculated for C_15_H_8_FN_2_O_2_S: 299.0285; found: 299.0280. HPLC retention time = 12.31 min, **3c** purity is 96%.

A more apolar compound, **4c**, was also isolated as yellow solid (0.0110g, 11%). ^1^H NMR (400 MHz, DMSO-*d_6_*): δ= 7.26–7.31 (2 H, m, 3′ and 5′-CH), 7.63–7.68 (2 H, m, 2′ and 6′-CH), 8.63 (1 H, s, 6-CH), 8.70 (1 H, d, *J* = 2.4 Hz, HetAr-H), 8.84 (1 H, d, *J* = 2.4 Hz, HetAr-H) ppm. ^13^C NMR (100.6 MHz, DMSO-d_6_): δ = 81.9 (C), 91.7 (C), 116.3 (C), 116.34 (d, *J* = 20.1Hz, 3′ and 5′-CH), 118.4 (d, *J* = 3.0 Hz, 1′-C), 134.1 (d, *J* = 8.0 Hz, 2′ and 6′-CH), 136.6 (6-CH), 141.9 (HetAr-CH), 142.9 (HetAr-CH), 147.8 (C), 154.0 (C), 162.4 (d, *J* = 248.5 Hz, CF) ppm. ^19^F NMR (282.85 MHz, DMSO-d_6_) δ = −105.9 ppm. HRMS (ESI) [M + H]^+^, *m*/*z* Calculated for C_14_H_8_FN_2_S: 255.0387, found 255.0389.

*8-(4-Methoxyphenyl)-6H-pyrano[4′,3′:4,5]thieno[2,3-b]pyrazin-6-one* (**3d**) *and 7-[(4-methoxyphenyl)ethynyl]thieno[2,3-b]pyrazine* (**4d**): Compound **1b** (0.0900 g, 0.347 mmol), 4-ethynylanisole (0.0520 g, 0.382 mmol) and heating for 1 h. Column chromatography using a solvent gradient from 10 to 40% ether/petroleum ether gave compound **3d** as a yellow solid (0.0570 g, 53%), m.p. 222–224 °C. ^1^H NMR (400 MHz, DMSO-*d_6_*): δ = 3.83 (3 H, s, OCH_3_), 7.09 (2 H, d, *J* = 8.8 Hz, 3′ and 5′-H), 7.80 (1 H, s, 9-H), 7.99 (2 H, d, *J* = 8.8 Hz, 2′ and 6-H), 8.96 (1 H, d, *J* = 2.4 Hz, HetAr-H), 9.05 (1 H, d, *J* = 2.4 Hz, HetAr-H) ppm. ^13^C NMR (100.6 MHz, DMSO-*d_6_*): δ = 55.6 (OCH_3_), 95.2 (9-CH), 114.8 (3′ and 5′-CH), 121.7 (C), 123.5 (C), 127.4 (2′ and 6′-CH), 142.1 (C), 143.96 (C), 143.98 (HetAr-CH), 145.8 (HetAr-CH), 157.8 (C), 158.1 (C), 158.4 (C), 161.5 (C) ppm. HRMS (ESI) [M + H]^+^, *m*/*z* Calculated for C_16_H_11_N_2_O_3_S: 311.0485; found: 311.0486. HPLC retention time = 12.68 min, **3d** purity is 97%.

A more apolar compound, **4d**, was also isolated as yellow solid (0.00900 g, 9%). ^1^H NMR (400 MHz, DMSO-d_6_): δ = 3.80 (3 H, s, OCH_3_), 7.00 (2 H, d, *J* = 8.8, Ar-H), 7.53 (2 H, d, *J* = 8.8, Ar-H), 8.59 (1 H, s, 6-CH), 8.71 (1 H, d, *J* = 2.4 Hz, HetAr-H), 8.85 (1 H, d, *J* = 2.4 Hz, HetAr-H) ppm. ^13^C NMR (100.6 MHz, DMSO-d_6_): δ = 55.4 (OCH_3_), 80.7 (C), 92.8 (C), 113.8 (C), 114.6 (2 × Ar-CH), 116.7 (C), 133.2 (2 × Ar-CH), 135.6 (6-CH), 141.7 (HetAr-CH), 142.8 (HetAr-CH), 147.8 (C), 153.9 (C), 159.9 (C) ppm. HRMS (ESI) [M + H]^+^, *m*/*z* Calculated for C_15_H_11_N_2_OS: 266.0514; found: 266.0510.

*8-(p-Tolyl)-6H-pyrano[4′,3′:4,5]thieno[2,3-b]pyrazin-6-one* (**3e**) *and 7-(p-tolylethynyl)thieno[2,3-b]pyrazine* (**4e**): Compound **1b** (0.100 g, 0.386 mmol), 4-ethynyltoluene (0.0490 g, 0.425 mmol) and heating for 2 h. Column chromatography using a solvent gradient from 5 to 40% ether/hexane gave compound **3e** as a yellow solid (0.0560 g, 50%), m.p. 241–243 °C. ^1^H NMR (400 MHz, DMSO-*d_6_*): δ = 2.39 (3 H, s, CH_3_), 7.36 (2 H, d, *J* = 8.0 Hz, 3′ and 5′-H), 7.78 (1 H, s, 9-H), 7.90 (2 H, d, *J* = 8.0 Hz, 2′ and 6′-H), 8.90 (1 H, d, *J* = 2.4 Hz, HetAr-H), 8.99 (1 H, d, *J* = 2.4 Hz, HetAr-H) ppm. ^13^C NMR (100.6 MHz, DMSO-*d_6_*): δ = 20.5 (CH_3_), 95.6 (9-CH), 122.1 (C), 125.1 (2′ and 6′-CH), 128.1 (C), 129.4 (3′ and 5′-CH), 140.5 (C), 141.3 (C), 143.45 (HetAr-CH), 143.5 (C), 145.1 (HetAr-CH), 157.1 (C), 157.9 (C), 158.0 (C) ppm. HRMS (ESI) [M + H]^+^, *m*/*z* Calculated for C_16_H_11_N_2_O_2_S: 295.0536; found: 295.0534. HPLC retention time = 18.5 min, **3e** purity is 99%.

A more apolar compound, **4e**, was also isolated as yellow solid (0.00400 g, 4%). ^1^H NMR (400 MHz, DMSO-*d_6_*): δ = 2.34 (3 H, s, CH_3_), 7.27 (2 H, d, *J* = 8.0 Hz, Ar-H), 7.49 (2 H, dd, *J* = 8.0 Hz, Ar-H), 8.64 (1 H, s, 6-H), 8.72 (1 H, d, *J* = 2.4 Hz, HetAr-H), 8.86 (1 H, d, *J* = 2.4 Hz, HetAr-H) ppm. HRMS (ESI) [M + H]^+^, *m*/*z* Calculated for C_15_H_11_N_2_S: 251.0637, found 251.0634.

*8-(Pyridin-2-yl)-6H-pyrano[4′,3′:4,5]thieno[2,3-b]pyrazin-6-one* (**3f**): Compound **1b** (0.100 g, 0.386 mmol), 2-ethynylpyridine (0.0450 g, 0.0440 mL, 0.425 mmol) and heating for 1 h. Column chromatography using a solvent gradient from 5 to 30% ethyl acetate/petroleum ether gave compound **3f** as a yellow solid (0.040 g, 50%), m.p. 238–239 °C. ^1^H NMR (400 MHz, DMSO-*d_6_*): δ = 7.53–7.56 (1 H, m, 5′-H), 8.02–8.03 (2 H, m, 3′ and 4′-H), 8.13 (1 H, s, 9-H), 8.74–8.76 (1 H, m, 6′-H), 8.95 (1 H, d, *J* = 2.4 Hz, HetAr-H), 9.05 (1 H, d, *J* = 2.4 Hz, HetAr-H) ppm. ^13^C NMR (100.6 MHz, DMSO-*d_6_*): δ = 97.6 (9-CH), 120.1 (3′ or 4′-CH), 124.6 (C), 125.4 (5′-CH), 138.0 (3′ or 4′-CH), 141.0 (C), 143.9 (C), 144.3 (HetAr-CH), 145.8 (HetAr-CH), 148.1 (C), 150.3 (6′-CH), 156.5 (C), 157.2 (C), 158.2 (C) ppm. HRMS (ESI) [M + H]^+^, *m*/*z* Calculated for C_14_H_8_N_3_O_2_S: 282.0332; found: 282.0333.

*8-(Thiophen-3-yl)-6H-pyrano[4′,3′:4,5]thieno[2,3-b]pyrazin-6-one* (**3g**) *and 7-(thiophen-3-ylethynyl)thieno[2,3-b]pyrazine* (**4g**): Compound **1b** (0.0900 g, 0.347 mmol), 3-ethynylthiophene (0.0430 g, 0.0390 mL, 0.382 mmol) and heating for 1 h. Column chromatography using a solvent gradient from 20 to 40% ethyl ether/petroleum ether gave compound **3g** as a yellow solid (0.0600 g, 54%), m.p. 247–249 °C. ^1^H NMR (400 MHz, DMSO-*d_6_*): δ = 7.73–7.75 (1 H, dd, *J* = 5.2 and 2.8 Hz, 5′-H), 7.81 (1 H, dd, *J* = 5.2 and 1.2 Hz, 4′-H), 7.84 (1 H, s, 9-H), 8.27 (1 H, dd, *J* = 2.8 and 1.2 Hz, 2′-H), 8.93 (1 H, d, *J* = 2.4 Hz, HetAr-H), 9.02 (1H, d, *J* = 2.4 Hz, HetAr-H) ppm. ^13^C NMR (100.6 MHz, DMSO-*d_6_*): δ = 96.3 (9-CH), 122.2 (C), 125.2 (4′-CH), 126.1 (2′-CH), 128.4 (5′-CH), 133.4 (C), 141.8 (C), 143.88 (C), 143.91 (HetAr-CH), 145.7 (HetAr-CH), 154.6 (C), 157.5 (C), 158.2 (C) ppm. HRMS (ESI) [M + H]^+^, *m*/*z* Calculated for C_14_H_9_N_2_O_2_S_2_: 301.0100; found: 301.0096.

A more apolar compound, **4g**, was also isolated as yellow solid (0.00200 g, 3%), ^1^H NMR (400 MHz, DMSO-*d_6_*): δ = 7.31 (1H, dd, *J* = 5.2 and 1.2 Hz, 4′-H), 7.67–7.69 (1 H, dd, *J* = 5.2 and 2.8 Hz, 5′-H), 7.97–7.98 (1 H, dd, *J* = 2.8 and 1.2 Hz, 2′-H), 8.64 (1 H, s, 6-H), 8.72 (1 H, d, *J* = 2.4 Hz, HetAr-H), 8.85 (1 H, d, *J* = 2.4 Hz, HetAr-H) ppm. HRMS (ESI) [M + H]^+^, *m*/*z* Calculated for C_13_H_7_N_2_O_2_S_2_: 286.9943; found: 286.9944.

##### General Procedure for the 9-HaloTricyclic Lactones (**5a** and **5b**)

In a vacuum-dried Schlenk tube with dry acetonitrile (3 mL), compound **2a** (1.0 equiv.), NXS (2.0 equiv.) and CuX_2_ (2.5 equiv.) were added and the mixture was heated and stirred at 100 °C, under Ar. After 1 h a precipitate came out, the mixture was cooled and filtered under vacuum to give compounds **5a** or 5**b**.

*9-Chloro-8-phenyl-6H-pyrano[4′,3′:4,5]thieno[2,3-b]pyrazin-6-one* (**5a**): Compound **2a** (0.0500 g, 0.170 mmol), NCS (0.0450 g, 0.340 mmol) and CuCl_2_ (0.0580 g, 0.425 mmol), gave compound **5a** as a yellow solid (0.0324 g, 60%), m.p. 313–314 °C. ^1^H NMR (400 MHz, DMSO-*d_6_*): δ = 7.57–7.59 (3 H, m, Ar-H), 7.82–7.84 (2 H, m, Ar-H), 8.98 (1 H, broad s, HetAr-H), 9.08 (1 H, broad s, HetAr-H) ppm. ^13^C NMR (100.6 MHz, DMSO-*d_6_*): δ = 108.2 (C), 125.2 (C), 128.3 (2 × Ar-CH), 129.0 (2 × Ar-CH), 130.2 (C), 130.4 (4′-CH), 136.6 (C), 143.6 (HetAr-CH), 143.8 (C), 144.8 (HetAr-CH), 153.5 (C), 156.5 (C), 157.5 (C) ppm. HRMS (ESI) [M + H]^+^, *m*/*z* Calculated for C_15_H_8_^35^ClN_2_O_2_S: 314.9989; found: 314.9989.

*9-Bromo-8-phenyl-6H-pyrano[4′,3′:4,5]thieno[2,3-b]pyrazin-6-one* (**5b**): Compound **2a** (0.0560 g, 0.190 mmol), NBS (0.0680 g, 0.381 mmol) and CuBr_2_ (0.107 g, 0.478 mmol), gave compound **5b** as a yellow solid (0.0340 g, 50%), m.p. 313–315 °C. ^1^H NMR (400 MHz, DMSO-d_6_): δ = 7.56 (3 H, m, Ar-H), 7.77 (2 H, m, Ar-H), 8.95 (1 H, broad s, HetAr-H), 9.06 (1 H, broad s, HetAr-H) ppm. ^13^C NMR (100.6 MHz, DMSO-d_6_): δ = 94.4 (C), 125.3 (C), 127.9 (2 × Ar-CH), 129.2 (2 × Ar-CH), 130.1 (4′-CH), 131.5 (C), 136.9 (C), 142.9 (HetAr-CH), 143.8 (C), 144.6 (HetAr-CH), 154.5 (C), 156.6 (C), 157.5 (C) ppm. HRMS (ESI) [M + H]^+^, *m*/*z* Calculated for C_15_H_8_^79^BrN_2_O_2_S: 358.9484; found: 358.9484. *m*/*z* Calculated for C_15_H_8_^81^BrN_2_O_2_S: 360.9464; found: 360.9464.

#### 3.1.2. Synthesis of Tetracyclic Lactones Derived from Thieno[2,3-*b*]quinoline by Rh(III)-Catalyzed Formal [4+2] Cycloaddition, Triggered by C-H Activation

##### General Procedure for the C-H Activation/Cycloaddition (**7a**–**f**)

In a Schlenk tube with dry DMF (0.8 mL), compound **6** (1.0 equiv.), [RhCp*Cl_2_]_2_ (2.5 mol%), Ag_2_CO_3_ (2.0 equiv.), AgSbF_6_ (10 mol%) and the internal alkyne (1.0 equiv.) were added. The reaction was left stirring at 100 °C, overnight, monitoring by TLC. After cooling, ethyl acetate (10 mL) was added, and the solvents were removed under vacuum. The resulting residue was then submitted to column chromatography to give compounds **7**.

*3,4-Diphenyl-1H-pyrano[4′,3′:4,5]thieno[2,3-b]quinolin-1-one* (**7a**): Compound **6** (0.0500 g, 0.218 mmol) and diphenylacetylene (0.0400 g, 0.218 mmol). Column chromatography using a solvent gradient from 5 to 40% ether/petroleum ether gave compound **7a** as a yellow solid (0.0500 g, 56%), m.p. 304–305 °C. ^1^H NMR (400 MHz, DMSO-d_6_, 80 °C): δ = 7.25 (1 H, s, 5-H), 7.30–7.35 (3 H, m, Ar-H), 7.44–7.47 (2 H, m, Ar-H), 7.50–7.66 (7 H, m, Ar-H), 7.87–7.91 (1 H, m, Ar-H), 8.07–8.10 (1 H, broad d, *J* = 8.4 Hz, Ar-H) ppm. ^13^C NMR (100.6 MHz, DMSO-*d_6_*, 80 °C): δ = 115.8 (C) 122.0 (C), 124.0 (C), 126.2 (CH), 127.0 (C), 127.3 (CH), 127.7 (2 × CH), 128.6 (2 × CH), 128.7 (2 × CH), 129.0 (2 × CH), 129.1 (CH), 130.6 (2 × CH), 131.7 (CH), 131.8 (C), 133.0 (C), 134.2 (5-CH), 141.2 (C), 147.3 (C), 154.3 (C), 157.1 (C), 162.2 (C) ppm. HRMS (ESI) [M + H]^+^, *m*/*z* Calculated for C_26_H_16_NO_2_S: 406.0896; found: 406.0897.

*3,4-Dibutyl-1H-pyrano[4′,3′:4,5]thieno[2,3-b]quinolin-1-one* (**7b**): Compound **6** (0.0500 g, 0.218 mmol) and 5-decyne (0.0300 g, 0.0390 mL, 0.218 mmol). Column chromatography using a solvent gradient from 5 to 20% ether/petroleum ether afforded compound **7b** as a yellow solid (0.0680 g, 85%), m.p.172–173 °C. ^1^H NMR (400 MHz, DMSO-*d_6_*, 80 °C): δ = 0.92–0.99 (6 H, m, 2 × CH_3_), 1.41–1.43 (2 H, m, CH_2_), 1.59–1.67 (6 H, m, 3 × CH_2_), 2.72–2.76 (2 H, t, *J* = 7.6 Hz, CH_2_), 3.05–3.09 (2 H, m, CH_2_), 7.71–7.75 (1 H, m, Ar-H), 7.94–7.99 (1 H, m, Ar-H), 8.11–8.13 (1H, broad d, *J* = 8.4 Hz, Ar-H), 8.31–8.33 (1 H, broad d, *J* = 8.4 Hz, Ar-H), 9.26 (1 H, s, 5-H) ppm. ^13^C NMR (100.6 MHz, DMSO-*d_6_*, 80 °C): δ = 13.7 (CH_3_), 13.9 (CH_3_), 21.7 (CH_2_), 21.8 (CH_2_), 25.8 (CH_2_), 29.5 (CH_2_) 29.8 (CH_2_), 31.5 (CH_2_), 113.6 (C), 121.8 (C), 125.1 (C), 126.5 (CH), 127.0 (C), 127.5 (CH), 130.0 (CH), 132.2 (CH), 135.7 (5-CH), 141.6 (C), 147.4 (C), 158.0 (C), 158.4 (C), 162.3 (C) ppm. HRMS (ESI) [M + H]^+^, *m*/*z* Calculated for C_22_H_24_NO_2_S: 366.1522; found: 366.1526.

*3,4-Bis(4-bromophenyl)-1H-pyrano[4′,3′:4,5]thieno[2,3-b]quinolin-1-one* (**7c**): Compound **6** (0.0500 g, 0.218 mmol) and bis(4-bromophenyl)acetylene (0.0730 g, 0.218 mmol). Column chromatography using a solvent gradient from 5 to 20% ethyl acetate/petroleum ether gave compound **7c** as a yellow solid (0.0920 g, 75%), m.p. 307–308 °C. ^1^H NMR (400 MHz, DMSO-d_6_, 80 °C): δ = 7.36 (2 H, d, *J* = 8.4 Hz, Ar-H), 7.40 (1 H, s, 5-H), 7.51 (2 H, d, *J* = 8.4 Hz, Ar-H), 7.56 (2 H, d, *J* = 8.4 Hz, Ar-H), 7.61–7.62 (2 H, m, Ar-H), 7.79 (2 H, d, *J* = 8.4 Hz, Ar-H), 7.88–7.92 (1 H, m, Ar-H), 8.08 (1 H, broad d, *J* = 8.8 Hz, Ar-H) ppm. ^13^C NMR (100.6 MHz, DMSO-*d_6_*, 80 °C): δ = 115.0 (C), 122.4 (C), 122.9 (C), 123.9 (C), 126.3 (CH), 126.7 (C), 127.2 (CH), 128.9 (CH), 130.7 (2 × CH), 130.8 (C), 130.9 (2 × CH), 131.8 (CH), 132.0 (C), 132.1 (2 × CH), 132.7 (2 × CH), 134.0 (5-CH), 140.5 (C), 147.3 (C), 153.4 (C), 156.8 (C), 162.0 (C) ppm. HRMS (ESI) [M + H]^+^, *m*/*z* Calculated for C_26_H_14_^79^Br_2_NO_2_S: 561.9106; found: 561.9105; *m*/*z* Calculated for C_26_H_14_^79^Br^81^BrNO_2_S: 563.9086; found: 563.9085; *m*/*z* Calculated for C_26_H_14_^81^Br_2_NO_2_S: 565.9066; found: 565.9064.

*3,4-Bis(4-methoxyphenyl)-1H-pyrano[4′,3′:4,5]thieno[2,3-b]quinolin-1-one* (**7d**): Compound **6** (0.0500 g, 0.218 mmol) and 1,2-bis(4-methoxyphenyl)ethyne (0.0520 g, 0.218 mmol). Column chromatography using a solvent gradient from 5 to 15% ethyl acetate/petroleum ether gave compound **7d** as a yellow solid (0.0620 g, 61%), m.p. 304–306 °C.^1^H NMR (400 MHz, DMSO-d_6_, 100 °C): δ = 3.77 (3 H, s, OCH_3_), 3.93 (3 H, s, OCH_3_), 6.88 (2 H, d, *J* = 8.8 Hz, Ar-H), 7.16 (2 H, d, *J* = 8.8 Hz, Ar-H), 7.38–7.45 (5 H, m, 5-H and 4 × Ar-H), 7.57–7.62 (2 H, m, Ar-H), 7.86–7.90 (1 H, m, Ar-H), 8.08 (1 H, broad d, *J* = 8.6 Hz, Ar-H) ppm. ^13^C NMR (100.6 MHz, DMSO-d_6_, 100 °C): δ = 54.8 (OCH_3_), 55.1 (OCH_3_), 113.3 (2 × CH), 114.6 (C), 114.7 (2 × CH), 121.0 (C), 123.9 (C), 124.2 (C), 125.2 (C), 126.0 (CH), 127.1 (C), 127.14 (CH), 128.7 (CH), 130.0 (2 × CH), 131.4 (CH), 131.7 (2 × CH), 134.2 (5-CH), 141.8 (C), 147.2 (C), 154.4 (C), 157.0 (C), 159.7 (C), 159.8 (C), 162.2 (C) ppm. HRMS (ESI) [M + H]^+^, *m*/*z* Calculated for C_28_H_20_NO_4_S: 466.1107; found: 466.1107.

*3,4-Bis(4-(trifluoromethyl)phenyl)-1H-pyrano[4’,3’:4,5]thieno[2,3-b]quinolin-1-one* (**7e**): Compound **6** (0.0400 g, 0.175 mmol) and 1,2-bis(4-(trifluoromethyl)phenyl)ethyne (0.0550 g, 0.175 mmol). Column chromatography using a solvent gradient from 5 to 30% ether/petroleum ether gave compound **7e** as a yellow solid (0.0470 g, 50%), m.p. 276–278 °C. ^1^H NMR (400 MHz, DMSO-*d_6_*, 80 °C): δ = 7.23 (1 H, s, 5-H), 7.50 (1 H, broad d, *J* = 8.4 Hz, Ar-H), 7.59–7.71 (5 H, m, Ar-H), 7.81 (2 H, d, *J* = 8.0 Hz, Ar-H), 7.83–7.93 (1 H, m, Ar-H), 9.94 (2 H, d, *J* = 8.0 Hz, Ar-H) 8.09 (1 H, broad d, *J* = 8.4 Hz, Ar-H) ppm. ^13^C NMR (100.6 MHz, DMSO-*d_6_*, 80 °C): δ = 115.6 (C), 122.3 (C), 123.1 (C) 123.4 (q, *J* = 271.2 Hz, CF_3_), 123.7 (q, *J* = 271.2 Hz, CF_3_), 124.6 (q, *J* = 3.0 Hz, 2 × CH), 125.8 (q, *J* = 3.0Hz, 2 × CH), 126.3 (CH), 126.6 (C), 127.3 (CH), 128.6 (CH), 129.6 (q, *J* = 32.2 Hz, CCF_3_), 129.7 (2 × CH), 129.9 (q, *J* = 32.2 Hz, CCF_3_), 131.8 (2 × CH), 131.9 (CH), 133.9 (5-CH), 135.4 (C), 137.0 (C), 140.1 (C), 147.4 (C) 152.9 (C), 156.7 (C), 162.0 (C) ppm. ^19^F NMR (282.85 MHz, DMSO-d_6_) δ = -57.4 (s), −57.0 (s) ppm. HRMS (ESI) [M + H]^+^, *m*/*z* Calculated for C_28_H_14_F_6_NO_2_S: 542.0644; found: 542.0643. HPLC retention time = 7.9 min, **7e** purity is 96%.

*4-Methyl-3-phenyl-1H-pyrano[4’,3’:4,5]thieno[2,3-b]quinolin-1-one* (**7f**): Compound **6** (0.0500 g, 0.218 mmol) and 1-phenyl-1-propyne (0.0250 g, 0.0270 mL, 0.218 mmol). Column chromatography using a solvent gradient from 5 to 10% ethyl acetate/petroleum ether gave compound **7f** as a yellow solid (0.0520 g, 70%), m.p. 301–302 °C. ^1^H NMR (400 MHz, DMSO-*d_6_*, 80 °C): δ = 2.72 (3 H, s, CH_3_), 7.55–7.61 (3 H, m, Ar-H), 7.66–7.73 (3 H, m, Ar-H), 7.93–7.97 (1 H, m, Ar-H), 8.12 (1 H, broad d, *J* = 8.8 Hz, Ar-H), 8.32–8.35 (1 H, broad, *J* = 8.8 Hz, Ar-H), 9.52 (1 H, s, 5-H) ppm. The regioisomer **7f** was assigned using nOe. NMR (100.6 MHz, DMSO-*d_6_*, 80 °C): δ = 15.0 (CH_3_), 110.1 (C), 122.2 (C), 124.9 (C), 126.0 (CH), 127.2 (CH), 127.6 (C), 128.2 (2 × CH), 129.1 (2 × CH); 129.5 (CH), 129.6 (CH), 131.7 (CH), 131.9 (C), 135.7 (5-CH), 142.2 (C), 147.3 (C), 154.3 (C), 157.3 (C), 162.2 (C) ppm. HRMS (ESI) [M + H]^+^, *m*/*z* Calculated for C_21_H_14_NO_2_S: 344.0740; found: 344.0740.

### 3.2. Biological Activities

#### 3.2.1. In Vitro Antitumor Evaluation (SRB Assay)

The human tumor cell lines used were CaCo-2 (colorectal adenocarcinoma), MCF-7 (breast adenocarcinoma), AGS (gastric adenocarcinoma), HeLa (cervical carcinoma), NCI-H460 (lung carcinoma). Non-tumor cell lines were also tested: PLP2 (primary pig liver culture) [32]. All the cell lines were maintained in RPMI-1640 medium supplemented with 10% fetal bovine serum, glutamine (2 mM), penicillin (100 U/mL), and streptomycin (100 mg/mL), with the exception of PLP2, which was maintained in DMEM medium supplemented with fetal bovine serum (10%), glutamine and antibiotics. The culture flasks were incubated at 37 °C and with 5% CO_2_, under a humid atmosphere. The cells were used only when they had 70 to 80% confluence. A known mass of each compound was dissolved in DMSO/H_2_O (1:1, 1 mL), in order to obtain the stock solutions with a concentration of 1 mM. From the latter, successive dilutions were made to obtain the concentrations to be tested (0.16–10 μM). Each compound concentration (10 μL) was incubated with the cell suspension (190 μL) of the cell lines tested in 96-well microplates for 72 h. The microplates were incubated at 37 °C and with 5% CO_2_ in a humid atmosphere after checking the adherence of the cells. All cell lines were tested at a concentration of 10,000 cells/well. After the incubation period, the cells were treated: TCA (10% *w*/*v*; 100 μL) was previously cooled, and plates were incubated for 1 h at 4 °C, washed with water, and, after drying, a SRB solution (0.057%, *m*/*v*; 100 μL) was added and left to stand at room temperature for 30 min. To remove non-adhered SRB, plates were washed three times with a solution of acetic acid (1% *v*/*v*) and placed to dry. Finally, an adhered SRB was solubilized with Tris (10 mM, 200 μL) and the absorbance at a wavelength of 540 nm was read in the Biotek ELX800 microplate reader. The results obtained by the SRB assay [16] were expressed regarding the concentration compounds that can inhibit cell growth by 50%—GI_50_. Doxorubicin was used as a positive control.

#### 3.2.2. In Vitro Anti-Parasitic Evaluation

##### Parasite Cultures

*T. brucei* Lister 427 bloodstream forms were grown in a humidified incubator at 37 °C, 5% CO_2_ in complete HMI-9 medium 1 supplemented with 10% heat-inactivated Fetal Bovine Serum (FBS) and 100 UI/mL penicillin/streptomycin. Parasites maintenance was performed in T25 ventilated flasks by subpassage at a concentration of 1 × 10^4^/mL every 2 days on T25 ventilated flasks.

*L. infantum* MHOM/MA/67/ITMAP-263 promastigotes forms were grown at 27 °C, in complete RPMI-1640 medium supplemented with 10% heat-inactivated Fetal Bovine Serum (FBS), 2 mM L-glutamine, 100 UI/mL penicillin/streptomycin, 20 mM HEPES. Parasites maintenance was performed in T25 non-ventilated flasks by subpassage at a concentration of 1 × 10^6^/mL every 5 days on T25 non-ventilated flasks [33].

##### Cell Cultures

Human leukemia cell line, THP-1 (ATCC^®^ TIB-202™, Manassas, VA, USA) was cultured in RPMI-1640 medium supplemented with 10% heat-inactivated Fetal Bovine Serum (FBS), 2 mM L-glutamine, 100 UI/mL penicillin/streptomycin, 20 mM HEPES. The cell line was maintained in a humidified incubator at 37 °C and 5% CO_2_ by subculture every three days in 20 mL of media at a concentration of 1 × 10^5^/mL in a T75 flask. All cell culture reagents were purchased from Lonza-Bioscience (Morrisville, NC, USA).

##### In Vitro Evaluation of Anti-*T. brucei* Activity

The efficacy of compounds against *T. brucei* bloodstream forms was evaluated using a modified resazurin-based assay previously described in the literature [34]. Parasites were added to 100 µL of serial dilutions of compounds in supplemented complete medium at a cell density of 5 × 10^3^/mL for. As a quality control, a dose–response curve for the antitrypanosomal pentamidine was included in all the assays. The final volume of the assay is 200 µL/well. Each condition was carried out in duplicate. Following 72 h incubation at the specific conditions for *T. brucei*, 20 µL of a 0.5 mM resazurin solution was added and plates were incubated for a further 4 h under the same conditions. Fluorescence was measured at 544 nm and 590 nm excitation and emission wavelength, respectively, using a Synergy 2 Multi-Mode Reader (Biotek, Winooski, VT, USA). Results were shown as % of parasite growth inhibition compared to control (untreated parasites) and represent the average of at least three independent experiments. The effect was evaluated by the determination of the IC_50_ value (concentration required to inhibit growth in 50%) and calculated by non-linear regression curves using GraphPad Prism version 8.1.1 for Windows (GraphPad Software, San Diego CA, USA).

##### Evaluation of Activity Against *L. infantum* MHOM/MA/67/ITMAP-263 Promastigotes

The compounds’ efficacy against *L. infantum* promastigotes was evaluated using a resazurin-based assay [35]. Parasites were added to 100 µL of serial dilutions of compounds in supplemented complete medium at a cell density of 5 × 10^5^/mL. As a quality control, a dose–response curve to the antileishmanial drug miltefosine was included in all the assays. The final volume of the assay was 200 µL/well. Each condition was carried out in duplicate. Following 72 h of incubation at the specific conditions for *L. infantum*, 20 µL of a 0.5 mM resazurin solution was added, and the plates were incubated for a further 4 h under the same conditions. The methodology to obtain the results was the same described in Section 3.2.2. for *T. brucei*.

##### Cytotoxicity in THP-1 Cells

The cytotoxicity effect of compounds on THP-1-derived macrophages was assessed by the colorimetric MTT assay (3-(4,5-dimethylthiazol-2-yl)-2,5-diphenyl tetrazolium bromide) [35]. Briefly, THP-1 cells were suspended in RPMI complete medium at a density of 1 × 10^6^ cells/mL and 100 μL/well were seeded in a 96-well plate and were differentiated into macrophages by addition of 40 ng/mL of phorbol-myristate 13-acetate (PMA, Sigma, Saint Louis, MI, USA) for 24 h followed by replacement with fresh medium for more 24 h. Subsequently, cells were incubated with 100 μL of compounds ranging from 100 to 12.5 μM after dilution in the RPMI complete medium. Each condition was carried out in quadruplicate. After 72 h of incubation at 37 °C 5% CO_2_, the medium was removed and 200 μL of 0.5 mg/mL MTT solution diluted in RPMI was added. Plates were incubated for an additional 4 h. Then 160 μL of media was removed and the same volume of 2-propanol was added. Absorbance was read at 570 nm using a Synergy 2 Multi-Mode Reader (Biotek, Winooski, VT, USA). Cytotoxicity was evaluated by the determination of the CC_50_ value (drug concentration that reduced the percentage of viable cells in 50%) and calculated by non-linear regression analysis using GraphPad Prism version 8.1.1 for Windows (GraphPad Software, San Diego, CA, USA). The results represent the average of at least three independent experiments. For each compound, the Selectivity Index (SI) was calculated as the ratio between cytotoxicity in THP-1 (CC_50_, 72 h) and activity against parasites (IC_50_, 72 h).

## 4. Conclusions

We successfully synthesized tricyclic and tetracyclic lactone derivatives of thieno[2,3-*b*]pyrazine or thieno[2,3-*b*]quinoline, and 2*H*-pyrones. First Pd/Cu-catalyzed Sonogashira coupling, using Et_3_N as a base, of methyl 7-bromothieno[2,3-*b*]pyrazine-6-carboxylate with (het)arylalkynes, which were either unsubstituted or substituted with EDGs or EWGs on the phenyl ring, and with either electron-rich or electron-deficient heteroaryl rings, were performed to obtain the Sonogashira ester products **2**, being the corresponding tricyclic lactones **3** isolated as minor products in some cases. An attempt was made to conduct the Sonogashira coupling using heterogeneous catalysis (Pd/C), excluding Cu, with a ligand and eucalyptol as the solvent for the synthesis of **2a**. However, **2a** was only obtained in moderate yield, along with a major unidentified product. Further studies are required to optimize and apply these more environmentally friendly conditions.

It was not possible to cyclize the Sonogashira product **2a** with TFA to lactone **3a**. The tricyclic lactones **3a**–**g** were obtained in good yields by a tandem one-pot Sonogashira coupling and 6-*endo-dig* lactonization of 7-bromothieno[2,3-*b*]pyrazine-6-carboxylic acid with (het)arylalkynes. Additionally, halogenated tricyclic lactones were synthesized from Sonogashira ester product **2a** by halocyclization using CuX_2_ and NXS, enabling further functionalization. With I_2_ or ICl the halocyclization did not occur.

Tetracyclic lactones **7** were synthesized through a Rh(III)-catalyzed formal [4+2] cycloaddition of thieno[2,3-*b*]quinoline-2-carboxylic acid with symmetrical or unsymmetrical internal alkyl or arylalkynes. The reaction was driven by C-H activation, with the carboxylic acid serving as a dynamic directing group to facilitate the cycloaddition. This methodology is atom-efficient and enables late-stage functionalization.

The biological activity of the Sonogashira ester products and lactones was assessed for both antitumor and antiparasitic potential. Antitumor activity was evaluated against five human tumor cell lines using the SRB assay. The most active compound was a Sonogashira ester product bearing a pyridine ring **3f**, exhibiting a GI_50_ value below 10 µM across all tested cell lines, with low toxicity, at their GI_50_ values, in non-cancerous PLP2 cells. The antiparasitic activity was assessed against *T. brucei* and *L. infantum*. Two compounds, **3f** and the 9-chloro tricyclic lactone **5a**, displayed notable activity against *T. brucei*, with IC_50_ values below 11 µM, though some degree of cytotoxicity was observed in THP-1-derived macrophages. These findings highlight the potential of the most promising compounds as lead candidates for the further development of antitumor and antiparasitic drugs, including investigations into their mechanisms of action. Additionally, all the compounds may be subjected to further biological activity assays to explore their full therapeutic potential.

## Figures and Tables

**Table 1:** Comparison of the Sonogashira conditions previously used by us with heterogeneous catalysis, excluding copper, using a more environmentally friendly solvent, in the synthesis of **2a**.

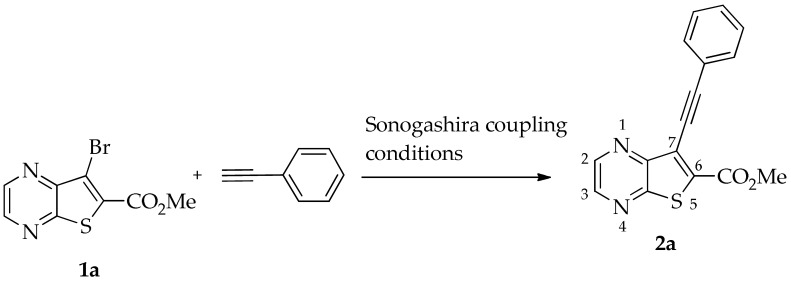							
Entry	Alkyne	Catalysts	Ligand	Base	Solvent	T/Time	2a Yield (%)
1	1.1 equiv	PdCl_2_(PPh_3_)_2_ 5 mol% CuI 5 mol%	_	Et_3_N (3 equiv.)	DMF	100 °C/3.5 h, under Ar	65% + tricyclic lactone **3a** (12%)
2	2.0 equiv	Pd/C 5 mol%	XPhos	K_2_CO_3_ (3 equiv.)	Eucalyptol	100 °C/5 h	30% + 60% of a compound ESI [M + H]^+^ *m*/*z* 397

**Table 2:** Synthesis of Sonogashira products **2** and tricyclic lactones **3** as minor products.

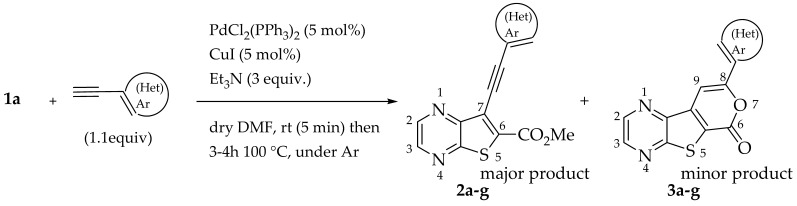		
Entry	Sonogashira Products	Tricyclic Lactones
1	**2a**, 65%	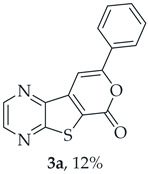
2	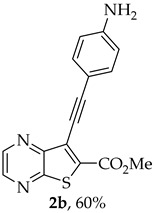	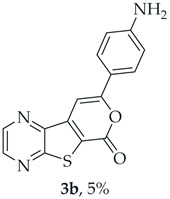
3	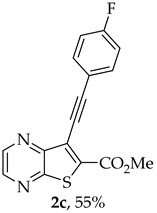	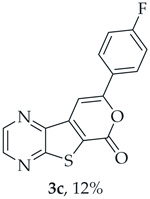
4	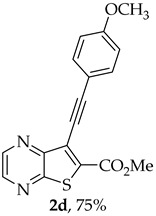	N.O.
5	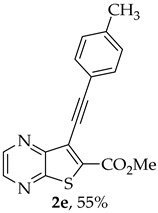	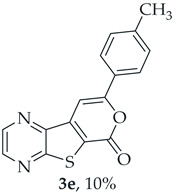
6	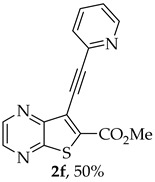	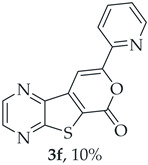
7	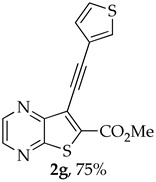	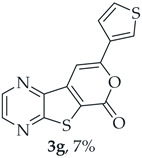
8	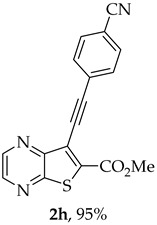	N.O.

N.O.—Not Observed.

**Table 3:** Synthesis of *6-endo-dig* tricyclic lactones **3** and decarboxylation products **4** of the non-isolable Sonogashira intermediate, as minor products.

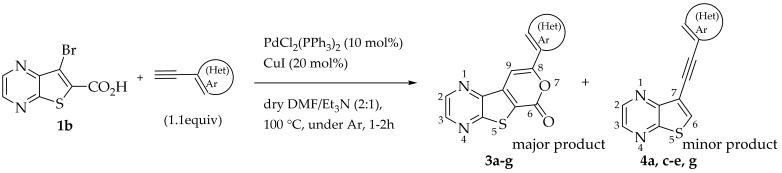		
Entry	Tricyclic Lactones	Decarboxylation Product
1	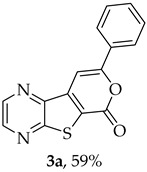	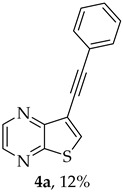
2	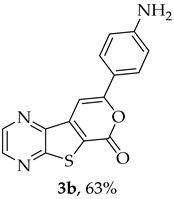	N.O.
3	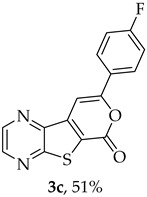	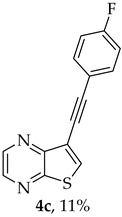
4	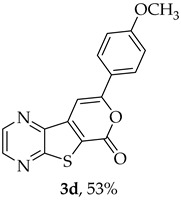	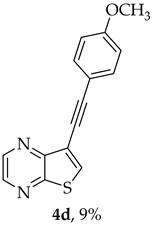
5	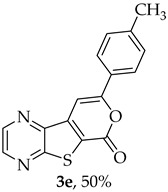	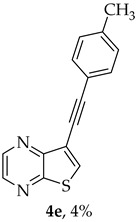
6	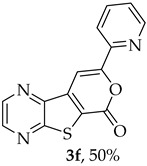	N.O.
7	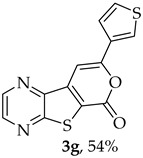	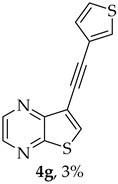

N.O.—Not Observed.

**Table 4:** Chemical shifts (δ) of 9-H in ^1^H NMR spectra (DMSO-*d_6_*) of compounds **3a**–**3g**.

Compounds 3	δ of 9-H Signal (ppm)
**3a**	7.93
**3b**	7.57
**3c**	7.96
**3d**	7.80
**3e**	7.78
**3f**	8.13
**3g**	7.84

**Table 5:** Synthesis of halogenated tricyclic lactones **5**.

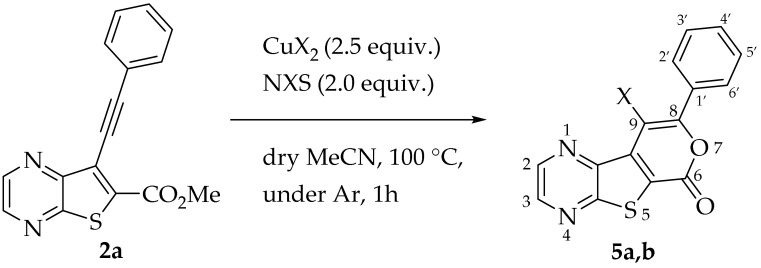			
Entry	Halogenated Tricyclic Lactones	Entry	Halogenated Tricyclic Lactones
1	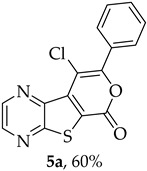	2	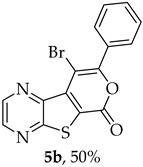

**Table 6:** Synthesis of tetracyclic lactones **7**.

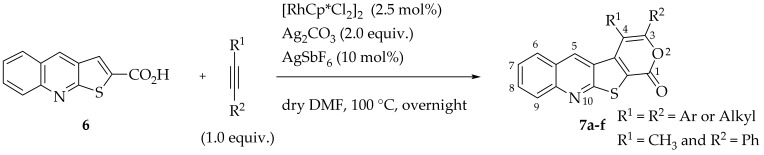			
Entry	Tetracyclic Lactones	Entry	Tetracyclic Lactones
1	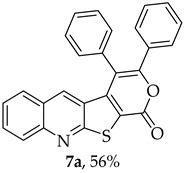	4	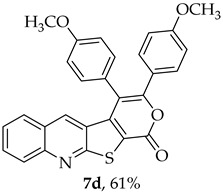
2	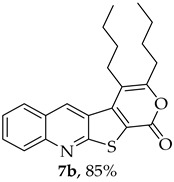	5	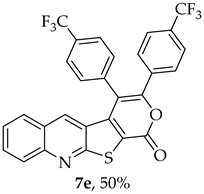
3	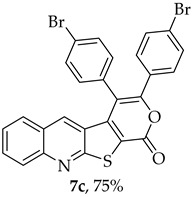	6	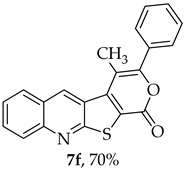

**Table 7:** Chemical shifts (δ) of 5-H in ^1^H NMR and the corresponding CH in the ^13^C NMR spectra (DMSO-*d*_6_ at 80 °C or 100 °C) of compounds **7a**–**7f**.

Tetracyclic Lactones	5-H Signal (ppm)	5-CH Signal (ppm)
**7a**	7.25	134.2
**7b**	9.26	135.7
**7c**	7.50	134.0
**7d**	7.40	134.2
**7e**	7.23	133.9
**7f**	9.52	135.7

**Table 8:** GI_50_ concentrations of compounds **2**, **3**, **5**, and **7** were evaluated against five human tumor cell lines and a non-tumor cell line (PLP2).

GI_50_ (µM) ^1^						
	Caco-2	MCF-7	AGS	HeLa	NCI-H460	PLP2
**2a**	70.03 ± 3.78	94.82 ± 6.43	86.78 ± 4.99	68.67 ± 3.05	91.45 ± 9.12	>125
**2b**	59.24 ± 1.76	49.73 ± 2.41	51.35 ± 2.87	47.92 ± 3.58	48.56 ± 4.31	>125
**2c**	52.11 ± 0.89	51.76 ± 1.07	70.73 ± 4.72	48.32 ± 1.66	67.21 ± 3.05	>125
**2d**	79.52 ± 3.58	67.89 ± 2.47	59.26 ± 1.97	62.33 ± 2.98	82.47 ± 5.35	>125
**2e**	92.97 ± 7.23	95.8 ± 8.08	89.17 ± 5.33	76.9 ± 6.55	101.67 ± 8.13	>125
**2f**	**3.72 ± 2.42**	**4.08 ± 0.27**	**9.25 ± 0.61**	**5.44 ± 4.31**	**9.33 ± 4.21**	**57.87 ± 4.31**
**2g**	30.73 ± 2.35	59.96 ± 3.19	46.05 ± 1.77	36.38 ± 2.85	62.13 ± 3.05	90.48 ± 6.47
**3a**	79.93 ± 3.56	77.52 ± 1.23	56.39 ± 3.76	49.73 ± 2.69	36.52 ± 0.69	>125
**3b**	67.49 ± 4.19	75.69 ± 6.48	74.28 ± 5.96	58.07 ± 4.29	82.74 ± 7.41	>125
**3c**	44.05 ± 0.93	56.1 ± 3.56	51.54 ± 3.29	48.38 ± 3.19	72.4 ± 6.24	>125
**3d**	90.48 ± 6.47	58.6 ± 1.86	56.2 ± 3.02	50.28 ± 4.79	45.33 ± 3.27	>125
**3e**	28.81 ± 1.85	47.55 ± 4.5	43.79 ± 1.76	38.21 ± 3.29	29.27 ± 1.47	90.48 ± 6.47
**3f**	58.76 ± 2.44	59.27 ± 2.45	42.65 ± 3.96	58.76 ± 2.44	61.25 ± 4.17	>125
**3g**	52.54 ± 1.31	48.63 ± 2.15	45.33 ± 0.89	38.98 ± 1.78	44.01 ± 3.28	>125
**5a**	41.71 ± 1.02	52.59 ± 3.18	36.34 ± 1.47	35.78 ± 2.08	51.54 ± 4.18	66.81 ± 3.58
**5b**	18.33 ± 0.92	45.14 ± 1.74	32.78 ± 1.56	29.06 ± 2.81	48.34 ± 1.08	89.76 ± 1.79
**7a**	75.04 ± 4.49	97.87 ± 6.31	93.49 ± 5.37	78.92 ± 6.06	93.88 ± 3.18	>125
**7b**	91.08 ± 2.46	100.78 ± 7.33	99.76 ± 4.94	82.79 ± 8.02	109.28 ± 9.16	>125
**7c**	87.76 ± 8.01	90.34 ± 4.16	103.6 ± 9.12	77.13 ± 1.12	108.41 ± 5.44	>125
**7d**	90.01 ± 3.29	91.23 ± 8.71	88.57 ± 5.08	75.18 ± 6.72	97.81 ± 6.14	>125
**7f**	87.02 ± 3.51	76.44 ± 2.58	83.44 ± 6.27	71.52 ± 2.36	92.08 ± 3.89	>125

^1^ GI_50_ values correspond to the compound concentration that causes 50% of cell growth inhibition. The results are reported as mean values ± standard deviation. GI_50_ (μM) for doxorubicin (positive control): CaCo-2: 0.04 ± 0.001; MCF-7: 0.05 ± 0.002; AGS: 0.03 ± 0.001; HeLa: 0.05 ± 0.002; NCI-H460: 0.07 ± 0.001; PLP2: 0.10 ± 0.008.

**Table 9:** Antiparasitic activity against *T. brucei* and *L. infantum* promastigotes and cytotoxity in THP-1—derived macrophages cells for compounds **2c–g**, **3a**, **5a** and **7**.

	*T.* *brucei*		*L. infantum*		CC_50_ Interval (μM) in THP-1-Derived Macrophages
	% of Activity ± ST.DEV (Single Dose 20 μM)	IC_50_ (μM)	% of Activity ± ST.DEV (Single Dose 20 μM)	IC_50_ (μM)	
**2c**	no activity *	—	no activity *	--	>100
**2d**	no activity *	--	no activity *	--	>100
**2e**	26 ± 7	--	no activity *	--	>100
**2f**	**98 ± 5**	**6.43**	57 ± 6	16	**100 > CC_50_ > 25**
**2g**	no activity *	--	no activity *	--	>100
**3a**	33 ± 5	--	30 ± 9	--	100 > CC_50_ > 50
**5a**	**74 ± 11**	**10.80**	32 ± 8	--	**100 > CC_50_ > 50**
**7a**	31 ± 7	--	no activity *	--	<12.5
**7b**	no activity *	--	51 ± 9	19	50 > CC_50_ > 12.5
**7c**	28 ± 7	--	41 ± 6	--	<12.5
**7d**	no activity *	--	no activity *	--	<12.5
**7e**	no activity *	--	no activity *	--	50 > CC_50_ > 25
**7f**	27 ± 3	--	34 ± 8	--	50 > CC_50_ > 12.5

* no significant antiparasitic activity at 20 μM, less than 20%. IC_50_ (μM) for Milefosine (positive control): *T. brucei:* 2.9 ± 0.5 μM; *L. infantum*: no activity.; THP-1 derived macrophages: 15.9 ± 1.2 μM. IC_50_ (μM) for Pentamidine (positive control): *L. infantum:* 2.94 ± 0.74 μM; *T. brucei:* no activity; THP-1: 47.8 ± 3.3 μM.

## Data Availability

The data presented in this study are available in the article and in the Supplementary Materials.

## Supplementary Materials

The following supporting information can be downloaded at: https://www.mdpi.com/article/10.3390/molecules30091999/s1, and contains ^1^H and ^13^C NMR spectra of the compounds **1b**, **2a**–**2g**, **3a**–**3g**, **4a**, **4c**–**4e**, **4g**, **5a**–**5b** and **7a**–**7f**, a nOe experiment on **7f**, ^1^H-^13^C 2D correlations HSQC spectra of compounds **7**, ^19^F NMR spectra of compounds **2c**, **3c**, **4c** and **7e** and HPLC chromatograms of compounds **3b**, **3c**, **3d**, **3e** and **7e** are available online.
