# Supplementary material for: Synthesis of Tricyclic and Tetracyclic Lactone Derivatives of Thieno[2,3-b]pyrazine or Thieno[2,3-b]quinoline: Preliminary Antitumor and Antiparasitic Activity Evaluation
Source: Molecules. 2025 Apr 30;30(9):1999. doi: 10.3390/molecules30091999 (PMC12073635; doi:10.3390/molecules30091999)

Supporting Information

Synthesis of Tricyclic and Tetracyclic Lactone Derivatives of Thieno[2,3-b]pyrazine or Thieno[2,3-b]quinoline: Preliminary Antitumor and Antiparasitic Activity Evaluation

Maria F. Martins [1], Francisco Ribeiro [1], Ana Borges [2,3], Ricardo C. Calhelha [2,3], Nuno Santarém [4], Anabela Cordeiro-da-Silva [4,5] and Maria-João R. P. Queiroz [1,*]

1 Centro de Química, Universidade do Minho (CQ-UM), Campus de Gualtar, 4710-057 Braga, Portugal; mariamartinst19@gmail.com (M.F.M.); franciscoribeiroo1113@gmail.com (F.R.)

2 Centro de Investigação de Montanha (CIMO), Instituto Politécnico de Bragança, Campus de Santa Apolónia 5300-253 Bragança, Portugal; ana.borges@ipb.pt (A.B.); calhelha@ipb.pt (R.C.C.)

3 Laboratório Associado para a Sustentabilidade e Tecnologia em Regiões de Montanha (SusTEC), Instituto Politécnico de Bragança, Campus de Santa Apolónia, 5300-253 Bragança, Portugal

4 Host-Parasite Interactions, IBMC/I3S, Rua Alfredo Allen, 208, 4200-135 Porto, Portugal; santarem@ibmc.up.pt (N.S.); cordeiro@i3s.up.pt (A.C.-d.-S.)

5 Laboratório de Microbiologia, Departamento de Ciências Biológicas, Faculdade de Farmácia, Universidade do Porto, 4050-313 Porto, Portugal

* Correspondence: mjrpq@quimica.uminho.pt

Supporting Information

1. NMR spectra of compound 1b **5**

^{1}H NMR (DMSO-d_6, 400 MHz) of compound **1b** 5

^{13}C NMR (DMSO-d_6, 100.6 MHz) of compound **1b** 5

2. NMR spectra of compound 2a **6**

^{1}H NMR (DMSO-d_6, 400 MHz) of compound **2a** 6

^{13}C NMR (DMSO-d_6, 100.6 MHz) of compound **2a** 6

3. NMR spectra of compound 2b **7**

^{1}H NMR (DMSO-d_6, 400 MHz) of compound **2b** 7

^{13}C NMR (DMSO-d_6, 100.6 MHz) of compound **2b** 7

4. NMR spectra of compound 2c **8**

^{1}H NMR (DMSO-d_6, 400 MHz) of compound **2c** 8

^{13}C NMR (DMSO-d_6, 100.6 MHz) of compound **2c** 8

^{19}F NMR (DMSO-d_6, 282.85 MHz) of compound **2c** 9

5. NMR spectra of compound 2d **10**

^{1}H NMR (DMSO-d_6, 400 MHz) of compound **2d** 10

^{13}C NMR (DMSO-d_6, 100.6 MHz) of compound **2d** 10

6. NMR spectra of compound 2e **11**

^{1}H NMR (DMSO-d_6, 400 MHz) of compound **2e** 11

^{13}C NMR (DMSO-d_6, 100.6 MHz) of compound **2e** 11

7. NMR spectra of compound 2f **12**

^{1}H NMR (DMSO-d_6, 400 MHz) of compound **2f** 12

^{13}C NMR (DMSO-d_6, 100.6 MHz) of compound **2f** 12

8. NMR spectra of compound 2g **13**

^{1}H NMR (DMSO-d_6, 400 MHz) of compound **2g** 13

^{13}C NMR (DMSO-d_6, 100.6 MHz) of compound **2g** 13

9. NMR spectra of compound 2h **14**

^{1}H NMR (DMSO-d_6, 400 MHz, 60 °C) of compound **2h** 14

^{13}C NMR (DMSO-d_6, 100.6 MHz, 60 °C) of compound **2h** 14

10. NMR spectra of compound 3a **15**

^{1}H NMR (DMSO-d_6, 400 MHz) of compound **3a** 15

^{13}C NMR (DMSO-d_6, 100.6 MHz) of compound **3a** 15

11. NMR spectra of compound 3b **16**

^{1}H NMR (DMSO-d_6, 400 MHz) of compound **3b** 16

^{13}C NMR (DMSO-d_6, 100.6 MHz) of compound **3b** 16

HPLC chromatogram of compound **3b**. 17

12. NMR spectra of compound 3c **18**

^{1}H NMR (DMSO-d_6, 400 MHz) of compound **3c** 18
^{13}C NMR (DMSO-d_6, 100.6 MHz) of compound **3c** 18
^{19}F NMR (DMSO-d_6, 282.85 MHz) of compound **3c** 19
HPLC chromatogram of compound **3c**. 19
13. NMR spectra of compound 3d 20
^{1}H NMR (DMSO-d_6, 400 MHz) of compound **3d** 20
^{13}C NMR (DMSO-d_6, 100.6 MHz) of compound **3d** 20
HPLC chromatogram of compound **3d**. 21
14. NMR spectra of compound 3e 22
^{1}H NMR (DMSO-d_6, 400 MHz) of compound **3e** 22
^{13}C NMR (DMSO-d_6, 100.6 MHz) of compound **3e** 22
HPLC chromatogram of compound **3e**. 23
15. NMR spectra of compound 3f 24
^{1}H NMR (DMSO-d_6, 400 MHz) of compound **3f** 24
^{13}C NMR (DMSO-d_6, 100.6 MHz) of compound **3f** 24
16. NMR spectra of compound 3g 25
^{1}H NMR (DMSO-d_6, 400 MHz) of compound **3g** 25
^{13}C NMR (DMSO-d_6, 100.6 MHz) of compound **3g** 25
17. NMR spectra of compound 4a 26
^{1}H NMR (DMSO-d_6, 400 MHz) of compound **4a** 26
^{13}C NMR (DMSO-d_6, 100.6 MHz) of compound **4a** 26
18. NMR spectra of compound 4c 27
^{1}H NMR (DMSO-d_6, 400 MHz) of compound **4c** 27
^{13}C NMR (DMSO-d_6, 100.6 MHz) of compound **4c** 27
^{19}F NMR (DMSO-d_6, 282.85 MHz) of compound **4c** 28
19. NMR spectra of compound 4d 29
^{1}H NMR (DMSO-d_6, 400 MHz) of compound **4d** 29
^{13}C NMR (DMSO-d_6, 100.6 MHz) of compound **4d** 29
20. NMR spectra of compound 4e 30
^{1}H NMR (DMSO-d_6, 400 MHz) of compound **4e** 30
21. NMR spectra of compound 4g 31
^{1}H NMR (DMSO-d_6, 400 MHz) of compound **4g** 31
22. NMR spectra of compound 5a 32
^{1}H NMR (DMSO-d_6, 400 MHz) of compound **5a** 32
^{13}C NMR (DMSO-d_6, 100.6 MHz) of compound **5a** 32
23. NMR spectra of compound 5b 33
^{1}H NMR (DMSO-d_6, 400 MHz) of compound **5b** 33

^{13}C NMR (DMSO-d_6, 100.6 MHz) of compound **5b** 33
24. NMR spectra of compound 7a **34**
^{1}H NMR (DMSO-d_6, 400 MHz, 80 °C) of compound **7a** 34
^{13}C NMR (DMSO-d_6, 100.6 MHz, 80 °C) of compound **7a** 34
Aromatic expansion of ^{1}H-^{13}C bidimensional correlations - HSQC spectrum of compound **7a** 35
25. NMR spectra of compound 7b **36**
^{1}H NMR (DMSO-d_6, 400 MHz, 80 °C) of compound **7b** 36
^{13}C NMR (DMSO-d_6, 100.6 MHz, 80 °C) of compound **7b** 36
Aromatic expansion of ^{1}H-^{13}C bidimensional correlations - HSQC spectrum of compound **7b** 37
26. NMR spectra of compound 7c **38**
^{1}H NMR (DMSO-d_6, 400 MHz, 80 °C) of compound **7c** 38
^{13}C NMR (DMSO-d_6, 100.6 MHz, 80 °C) of compound **7c** 38
Aromatic expansion of ^{1}H-^{13}C bidimensional correlations - HSQC spectrum of compound **7c** 39
27. NMR spectra of compound 7d **40**
^{1}H NMR (DMSO-d_6, 400 MHz, 100 °C) of compound **7d** 40
^{13}C NMR (DMSO-d_6, 100.6 MHz, 100 °C) of compound **7d** 40
Aromatic expansion of ^{1}H-^{13}C bidimensional correlations - HSQC spectrum of compound **7d** 41
28. NMR spectra of compound 7e **42**
^{1}H NMR (DMSO-d_6, 400 MHz, 80 °C) of compound **7e** 42
^{13}C NMR (DMSO-d_6, 100.6 MHz, 80 °C) of compound **7e** 42
Aromatic expansion of ^{1}H-^{13}C bidimensional correlations - HSQC spectrum of compound **7e** 43
^{19}F NMR (DMSO-d_6, 282.85 MHz) of compound **7e** 43
HPLC chromatogram of compound **7e** 44
29. NMR spectra of compound 7f **45**
^{1}H NMR (DMSO-d_6, 400 MHz, 80 °C) of compound **7f** 45
^{13}C NMR (DMSO-d_6, 100.6 MHz, 80 °C) of compound **7f** 45
Aromatic expansion of ^{1}H-^{13}C bidimensional correlations - HSQC spectrum of compound **7f** 46
nOe between the methyl group and the 5-H of compound **7f** 46

1. NMR spectra of compound 1b

^{1}H NMR (DMSO-*d*$_6$, 400 MHz) of compound **1b**

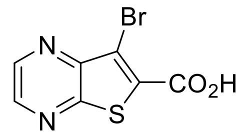

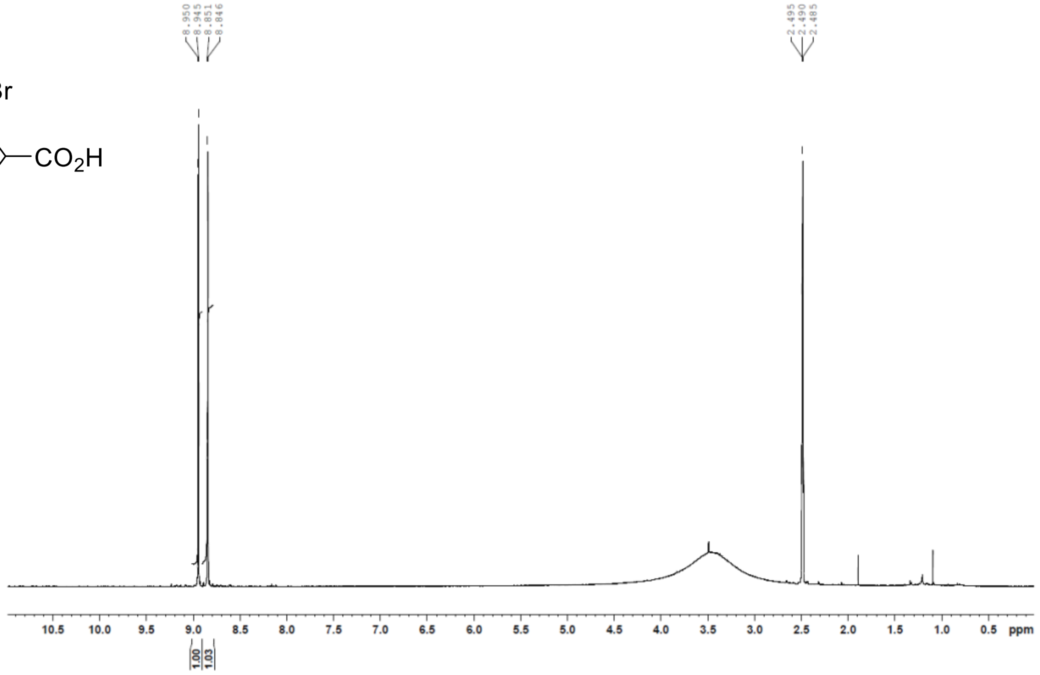

^{13}C NMR (DMSO-*d*$_6$, 100.6 MHz) of compound **1b**

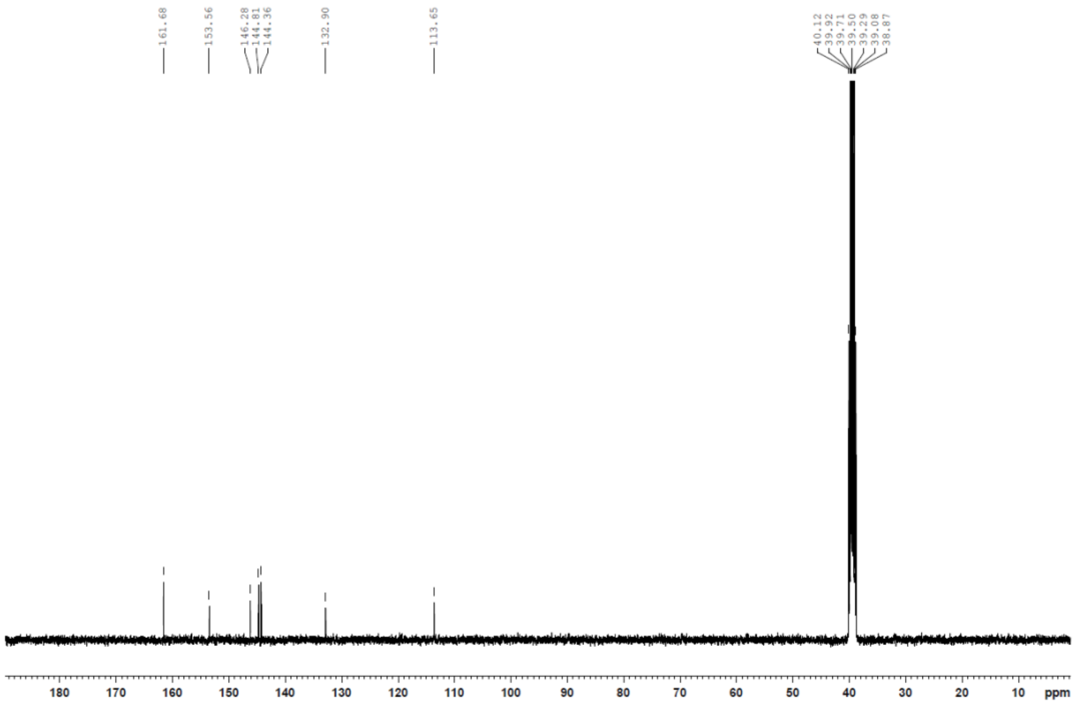

2. NMR spectra of compound 2a

^{1}H NMR (DMSO-d_6, 400 MHz) of compound **2a**

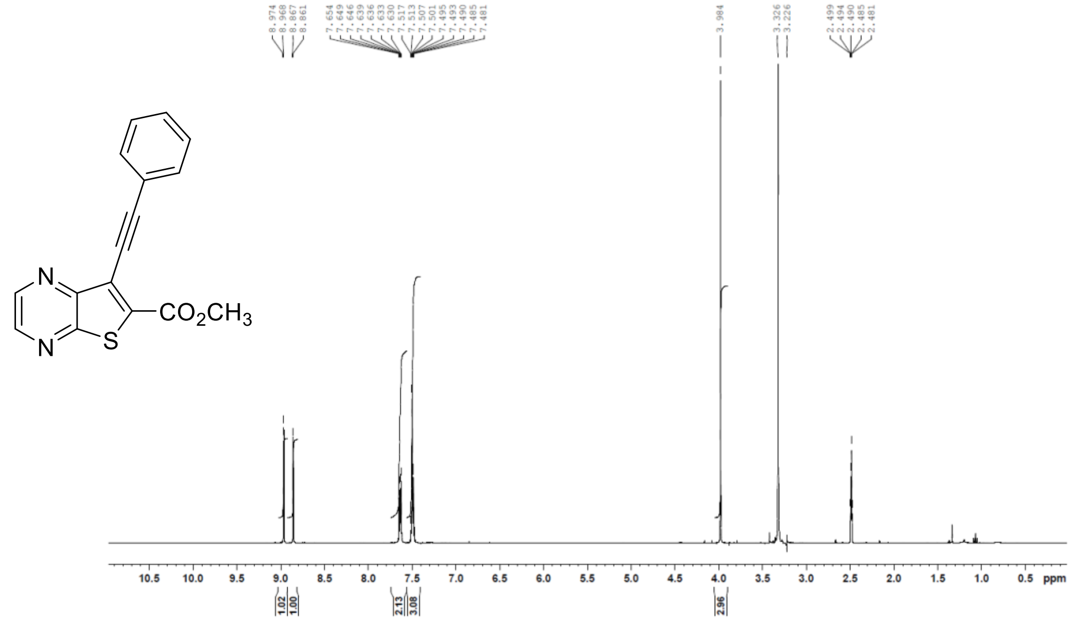

^{13}C NMR (DMSO-d_6, 100.6 MHz) of compound **2a**

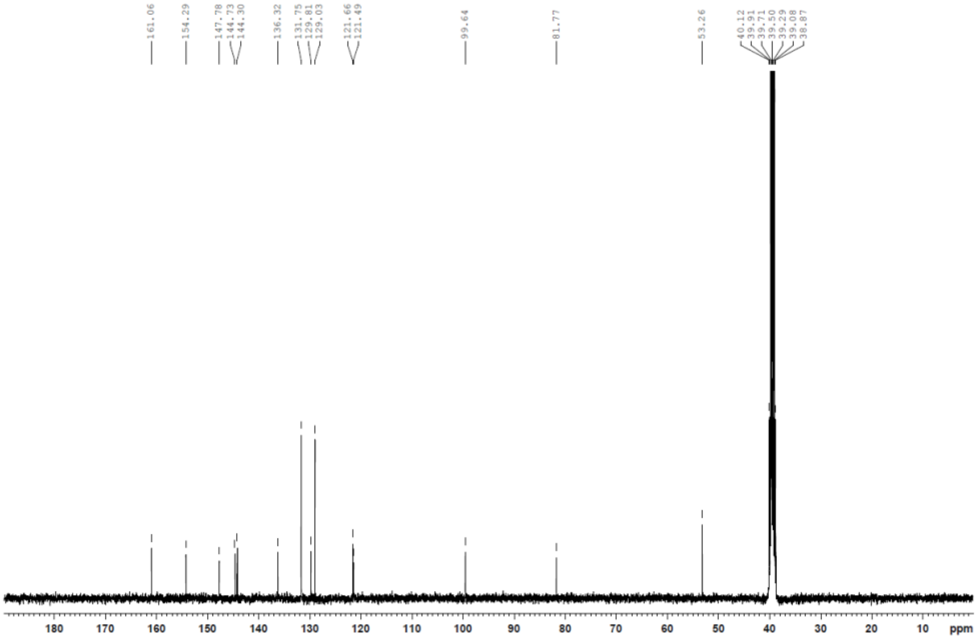

3. NMR spectra of compound 2b

^{1}H NMR (DMSO-d_6, 400 MHz) of compound **2b**

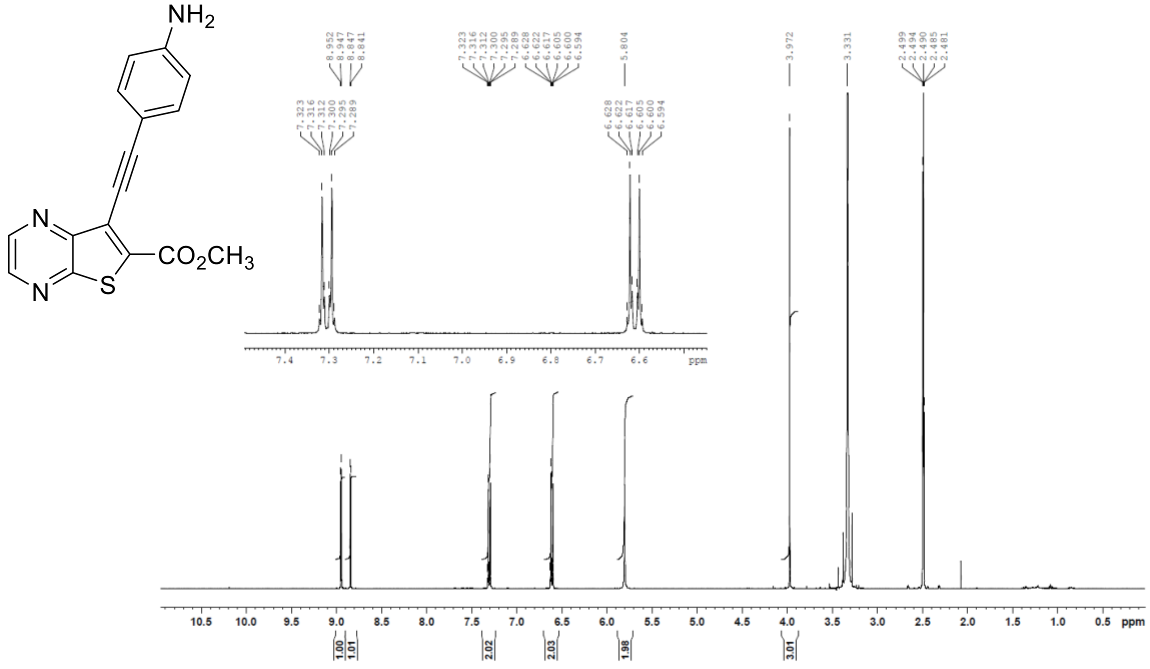

^{13}C NMR (DMSO-d_6, 100.6 MHz) of compound **2b**

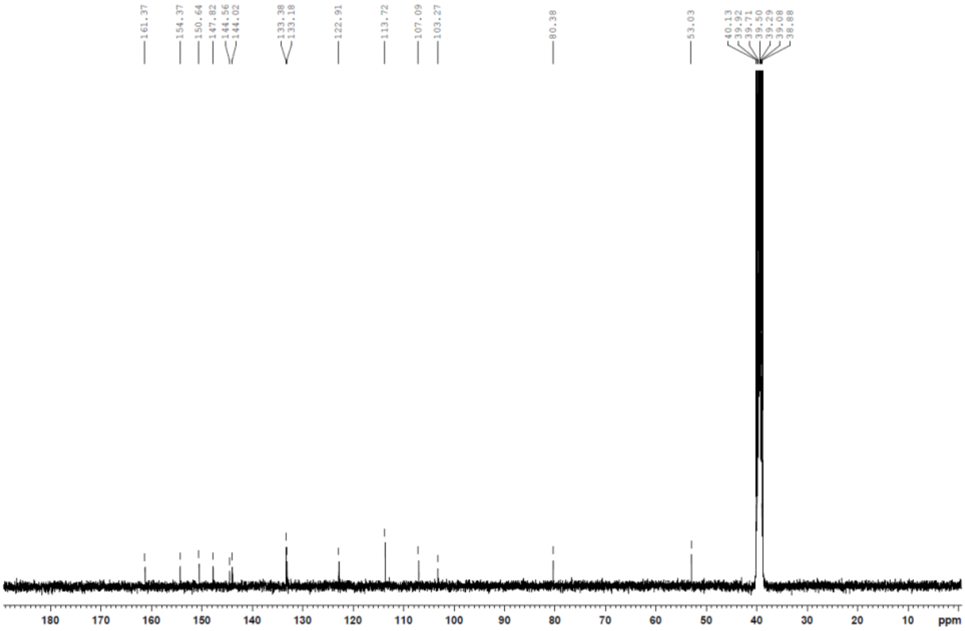

4. NMR spectra of compound 2c

^{1}H NMR (DMSO-d_6, 400 MHz) of compound **2c**

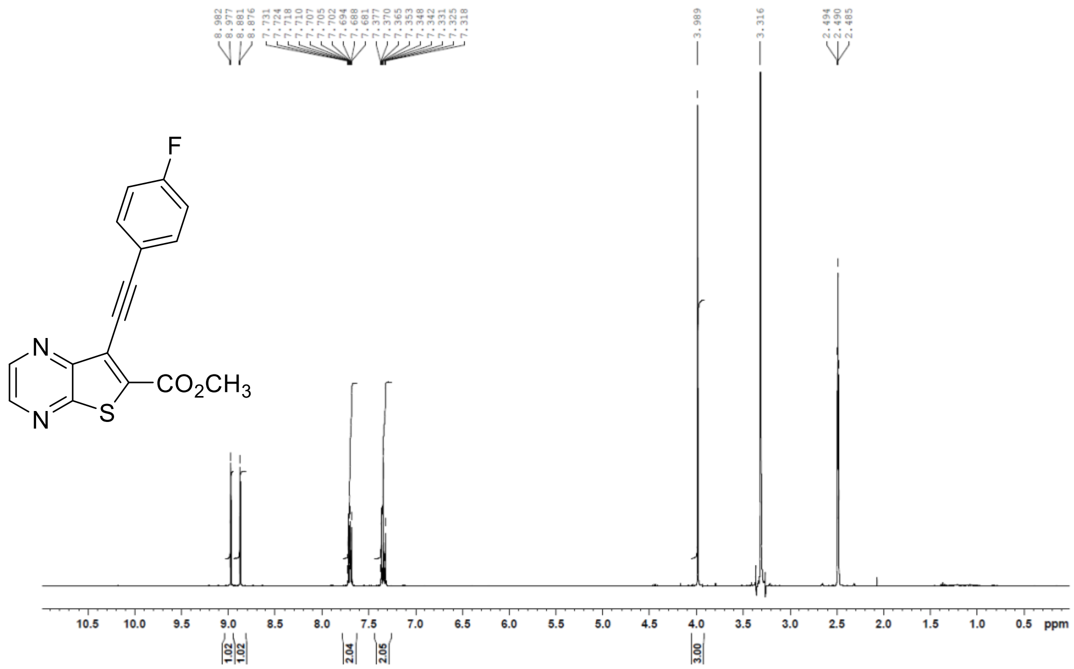

^{13}C NMR (DMSO-d_6, 100.6 MHz) of compound **2c**

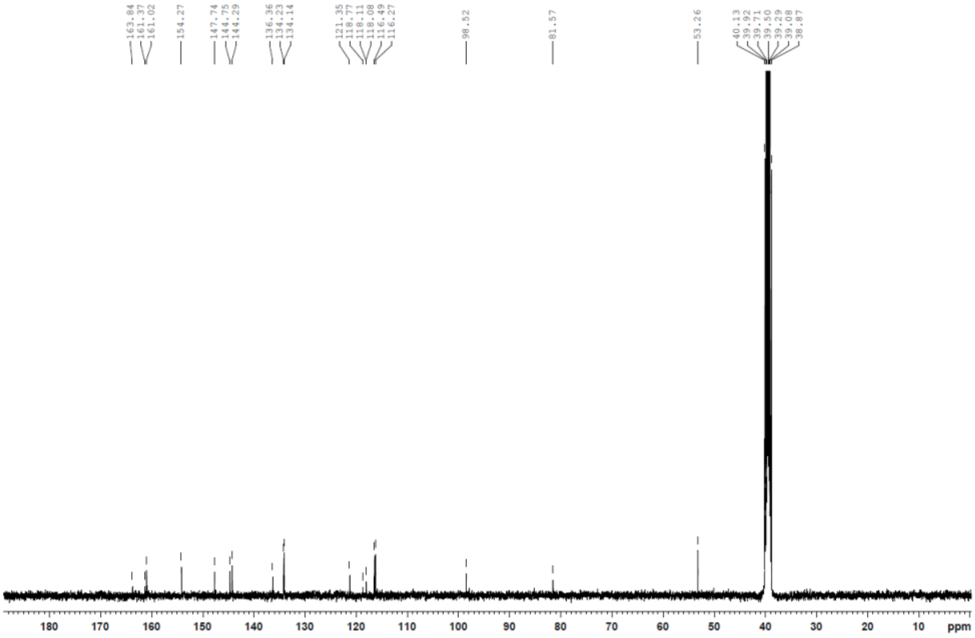

^{19}F NMR (DMSO-d_6, 282.85 MHz) of compound **2c**

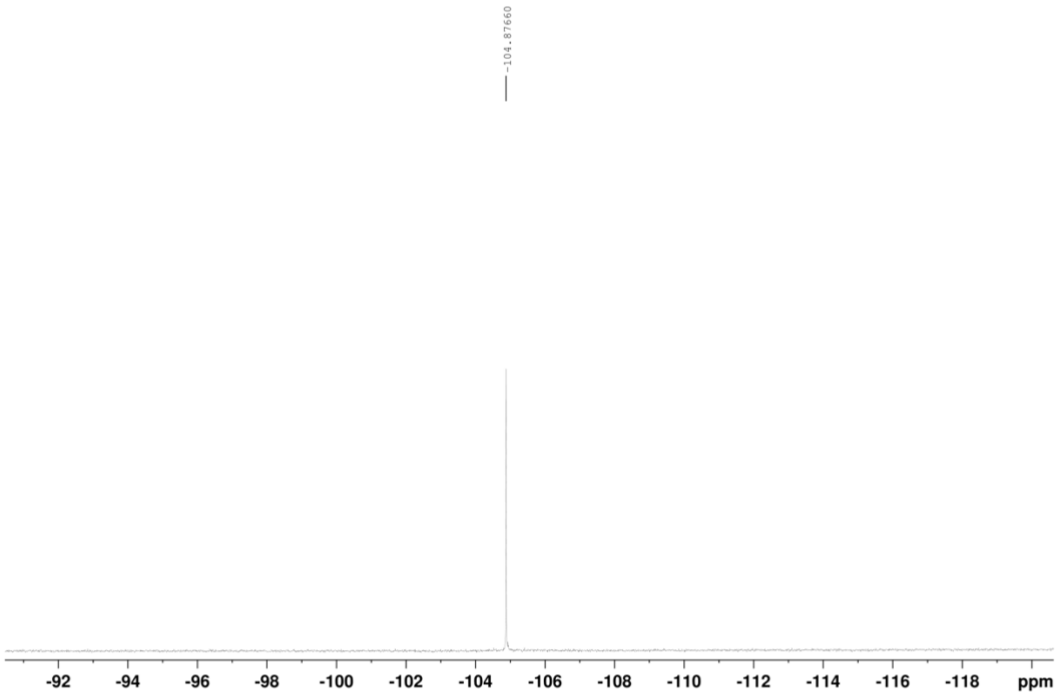

5. NMR spectra of compound 2d

^1H NMR (DMSO-d_6, 400 MHz) of compound **2d**

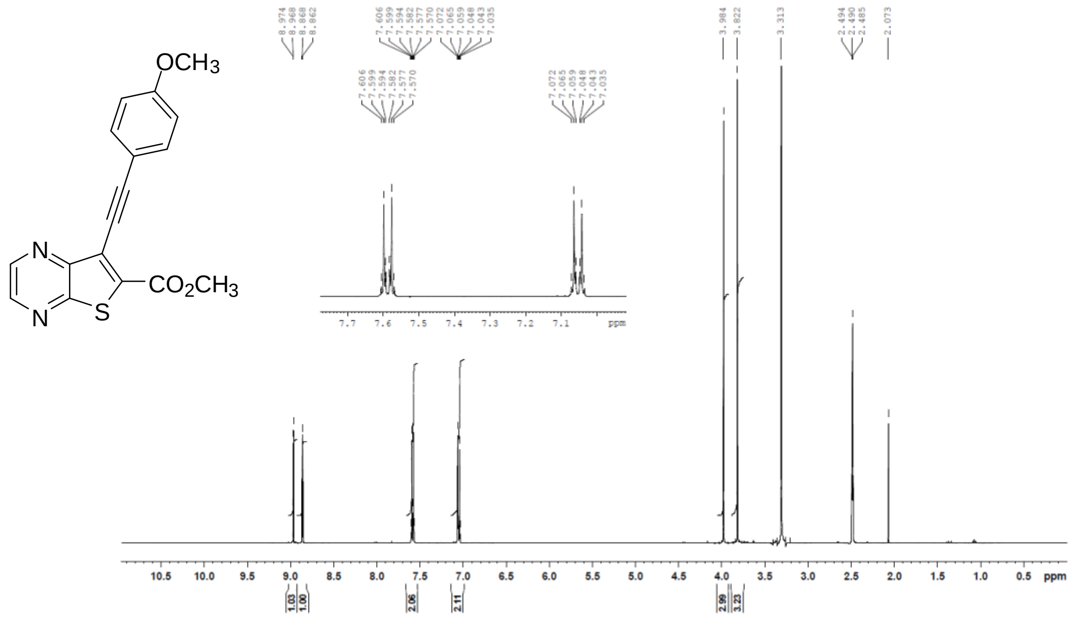

^{13}C NMR (DMSO-d_6, 100.6 MHz) of compound **2d**

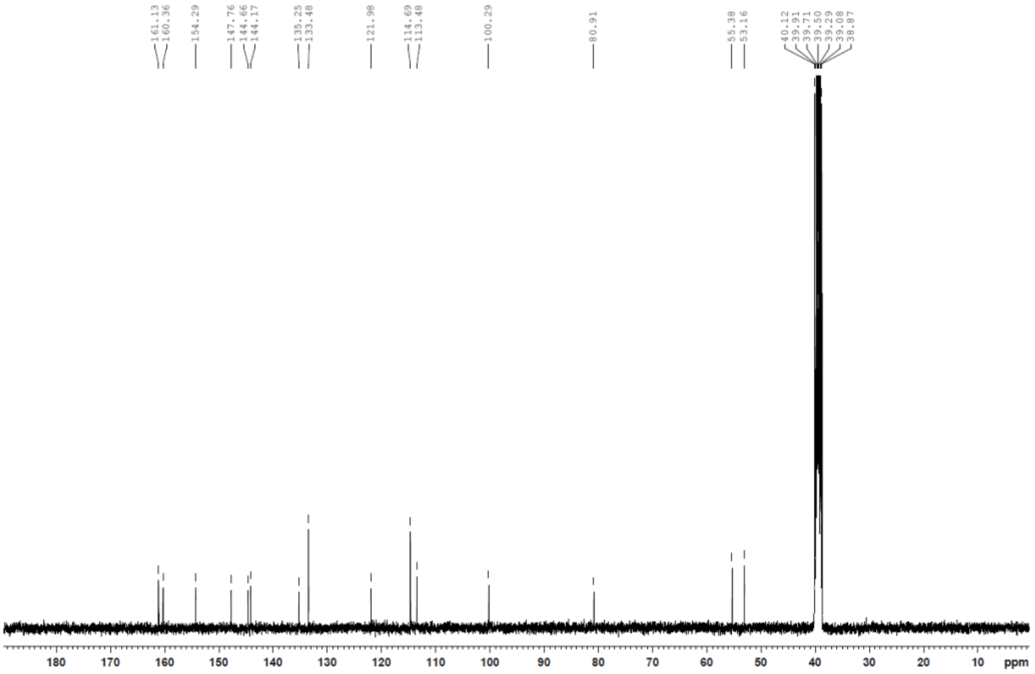

6. NMR spectra of compound 2e

^{1}H NMR (DMSO-d_6, 400 MHz) of compound **2e**

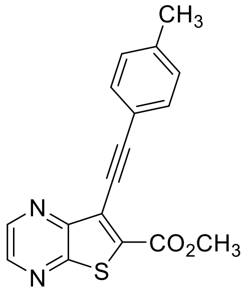

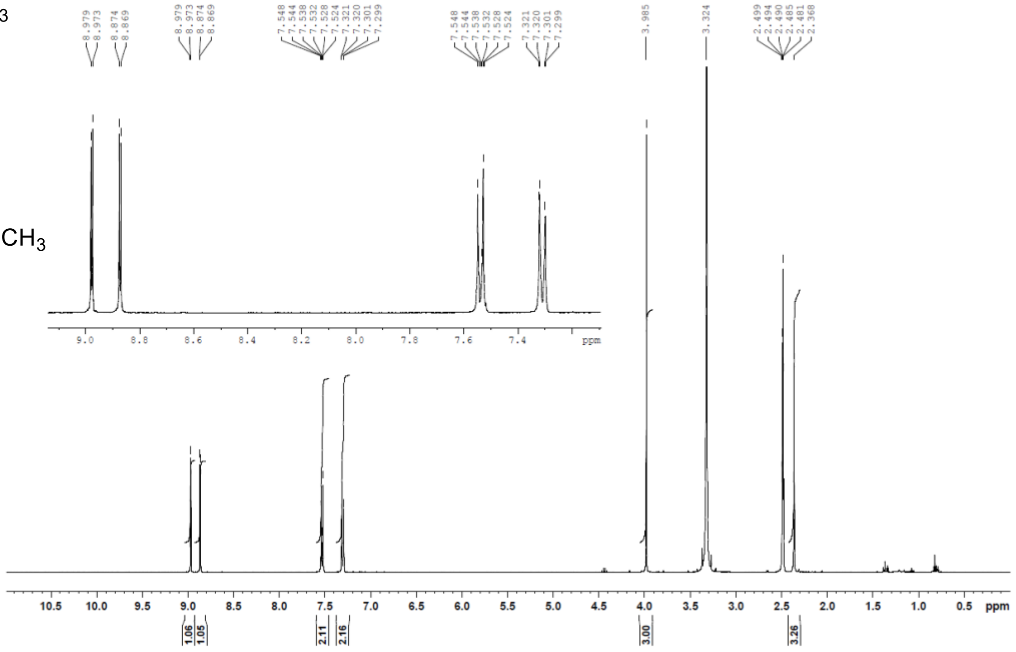

^{13}C NMR (DMSO-d_6, 100.6 MHz) of compound **2e**

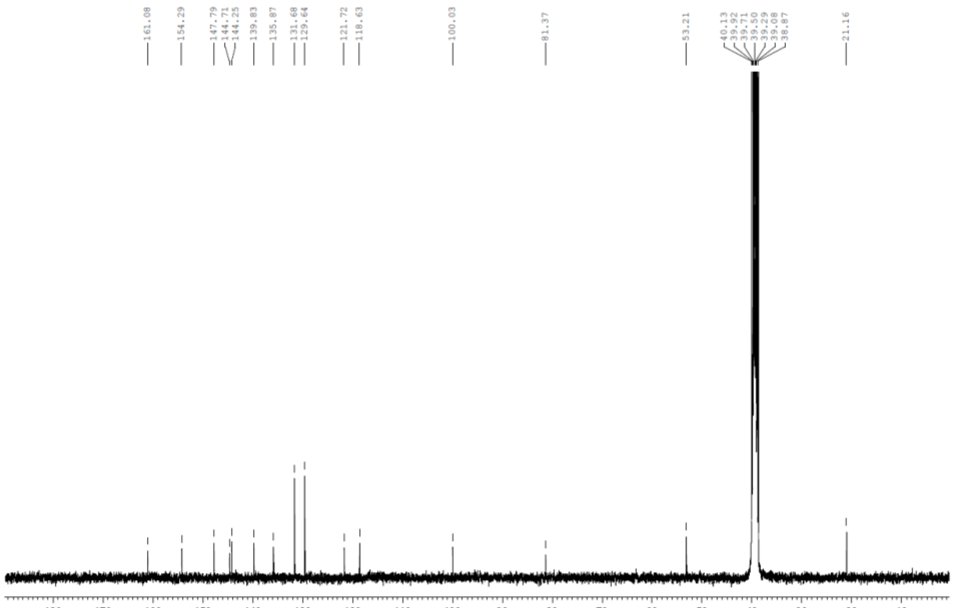

7. NMR spectra of compound 2f

^{1}H NMR (DMSO-d_6, 400 MHz) of compound **2f**

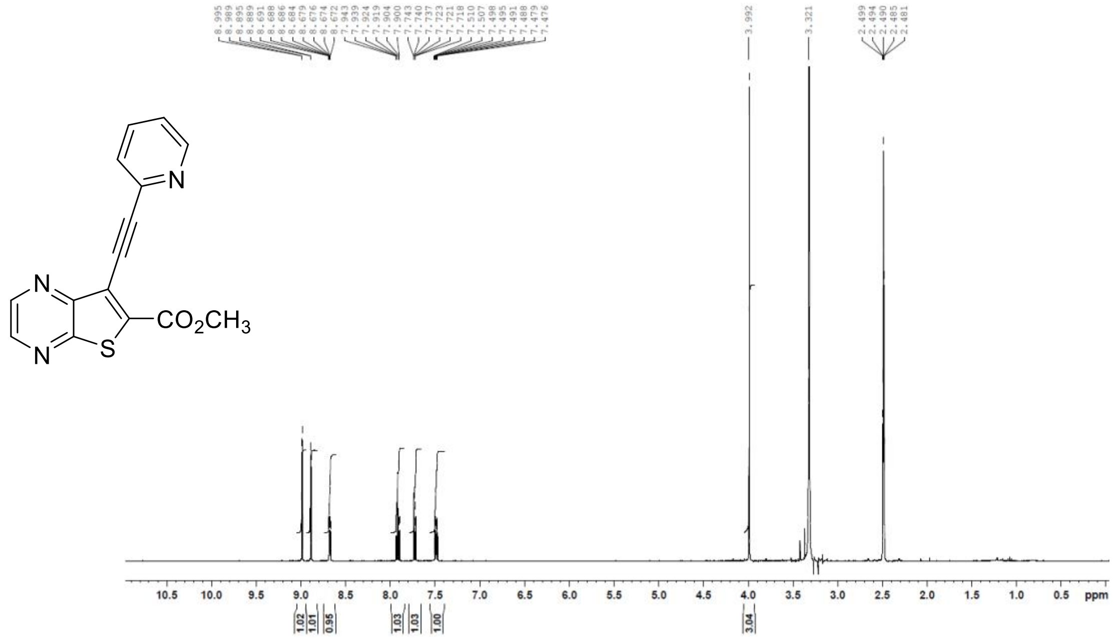

^{13}C NMR (DMSO-d_6, 100.6 MHz) of compound **2f**

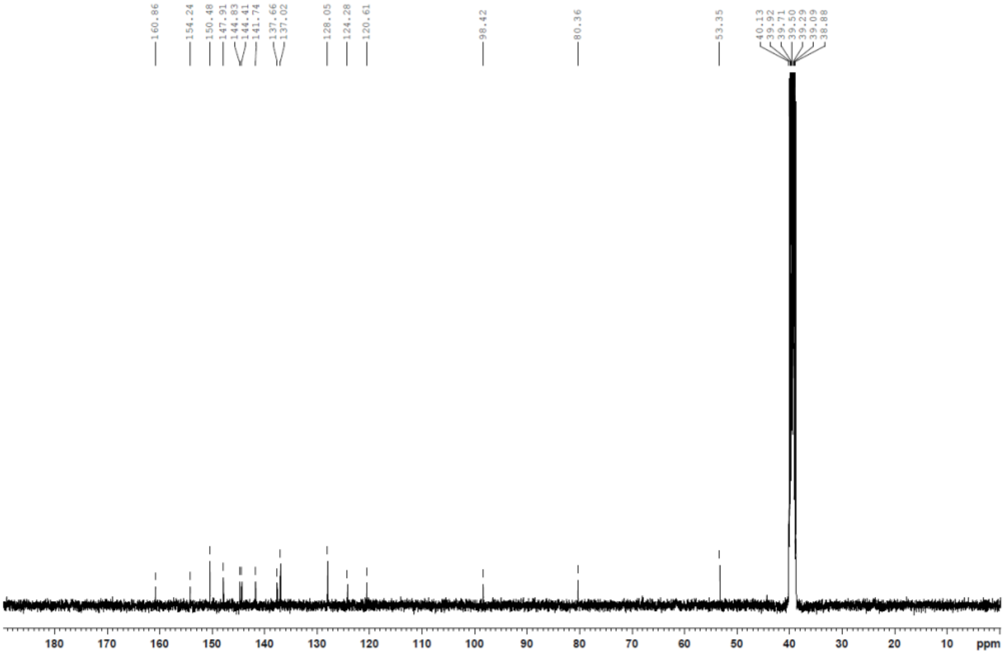

8. NMR spectra of compound 2g

^{1}H NMR (DMSO-d_6, 400 MHz) of compound **2g**

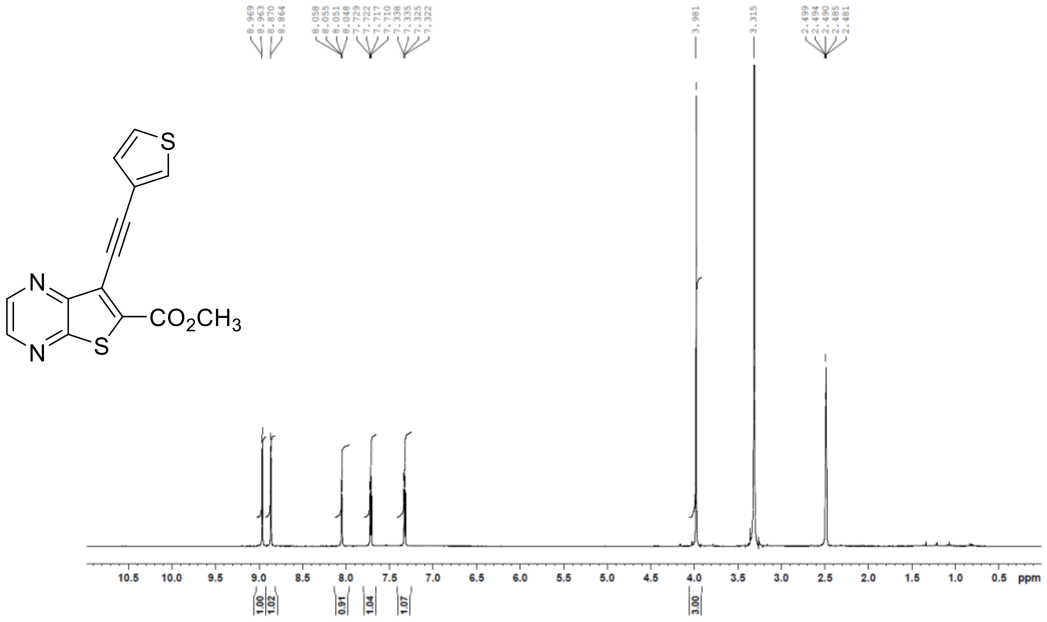

^{13}C NMR (DMSO-d_6, 100.6 MHz) of compound **2g**

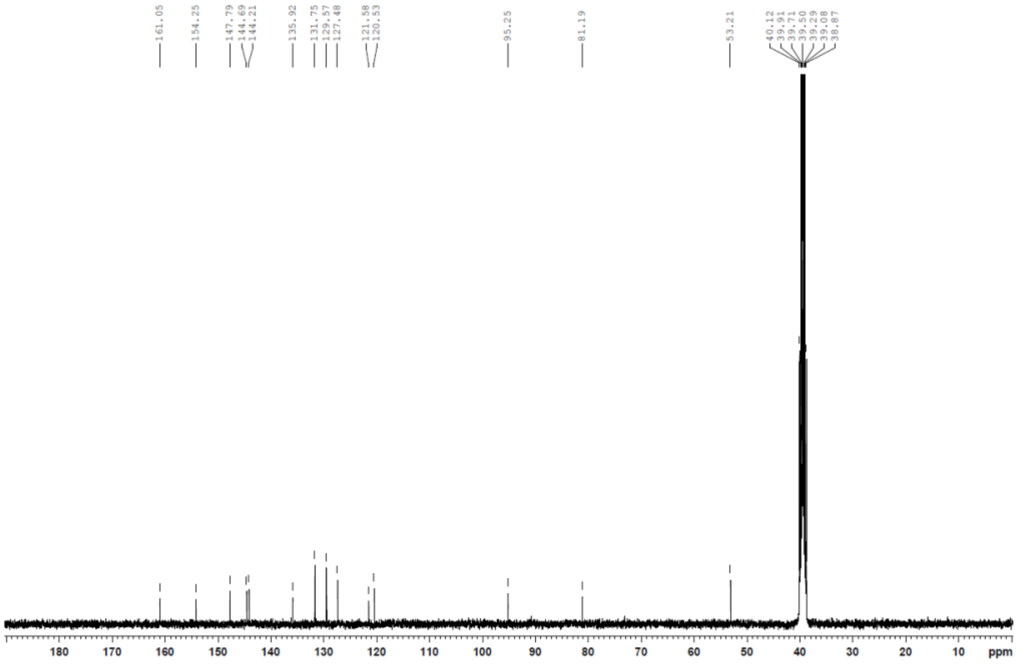

9. NMR spectra of compound 2h

^{1}H NMR (DMSO-*d*$_6$, 400 MHz, 60 °C) of compound **2h**

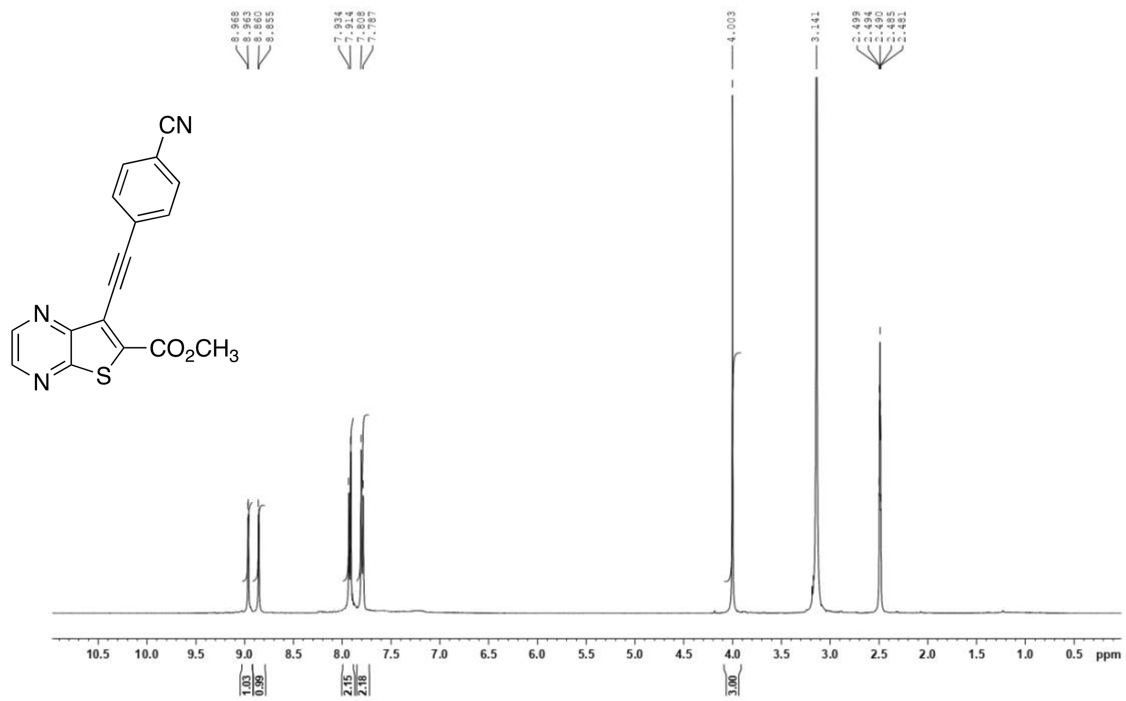

^{13}C NMR (DMSO-*d*$_6$, 100.6 MHz, 60 °C) of compound **2h**

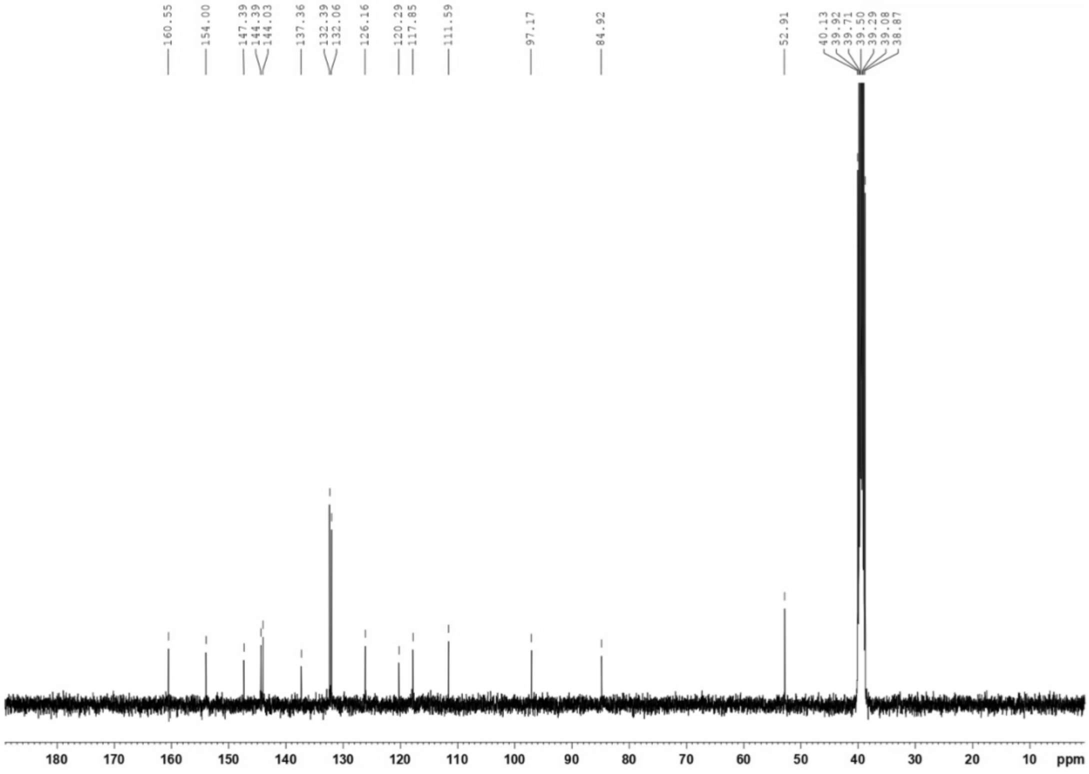

10. NMR spectra of compound 3a

^{1}H NMR (DMSO-d_6, 400 MHz) of compound **3a**

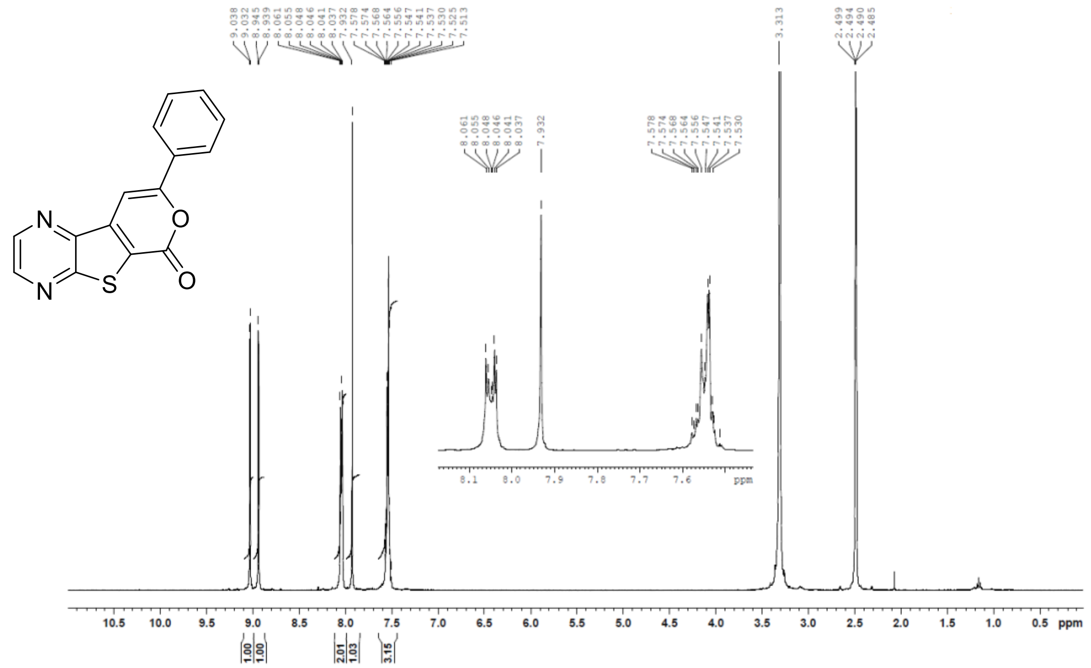

^{13}C NMR (DMSO-d_6, 100.6 MHz) of compound **3a**

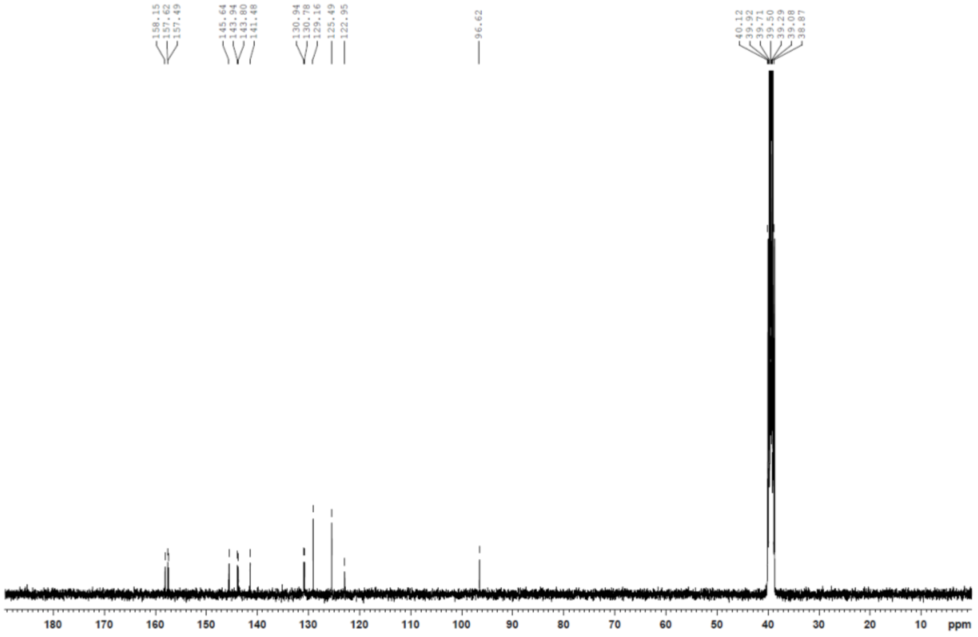

11. NMR spectra of compound 3b

^{1}H NMR (DMSO-d_6, 400 MHz) of compound **3b**

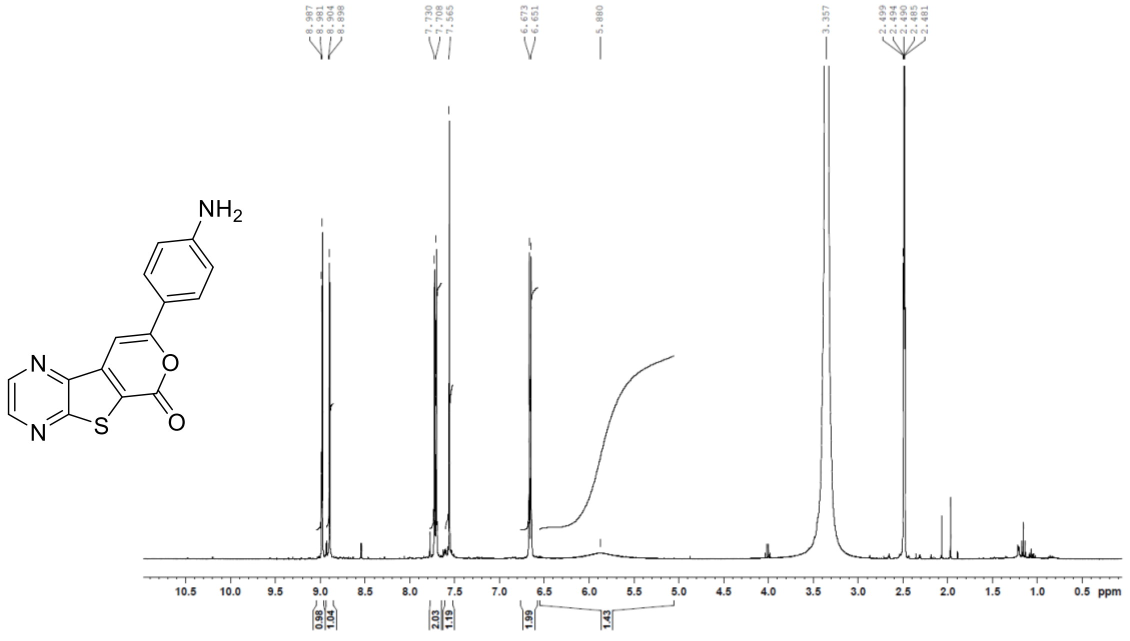

^{13}C NMR (DMSO-d_6, 100.6 MHz) of compound **3b**

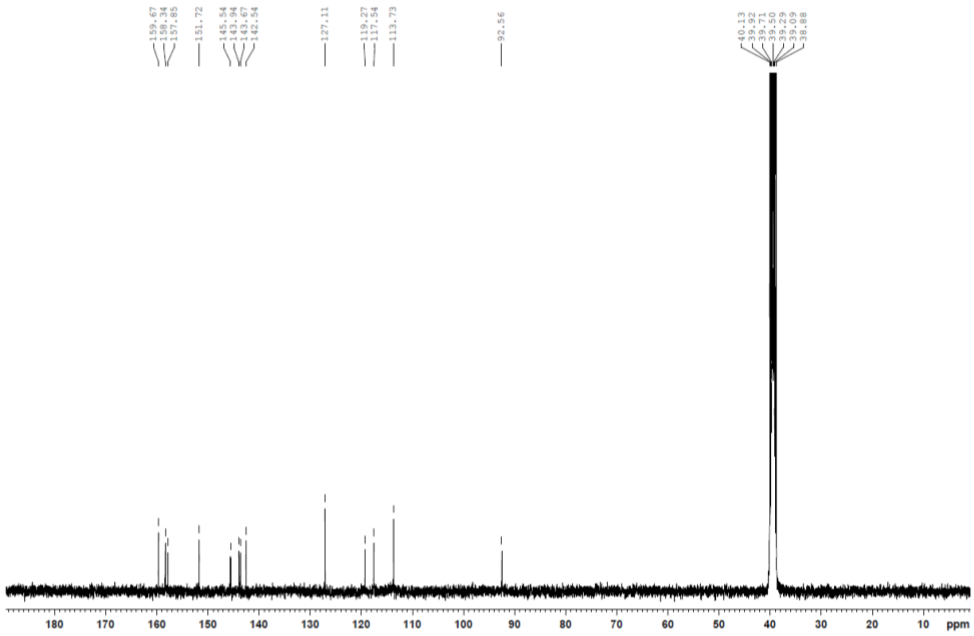

HPLC chromatogram of compound **3b**.

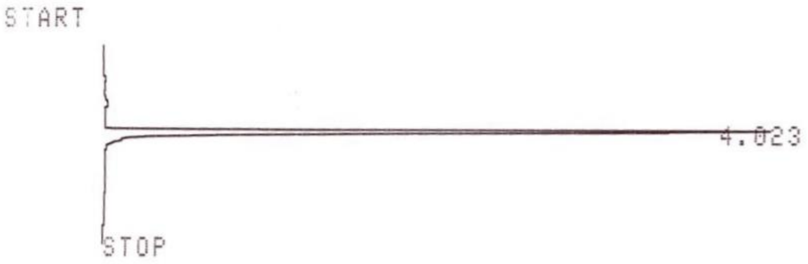

CHROMATOPAC C-R6A FILE 0
SAMPLE NO 0 METHOD 41
REPORT NO 2515

PKNO	TIME	AREA	MK	IDNO	CONC	NAME
1	4.023	62644			100	
	TOTAL	62644			100	

12. NMR spectra of compound 3c

^{1}H NMR (DMSO-*d*6, 400 MHz) of compound **3c**

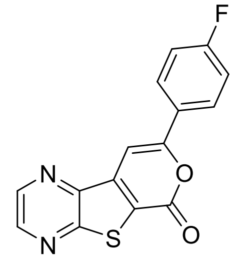

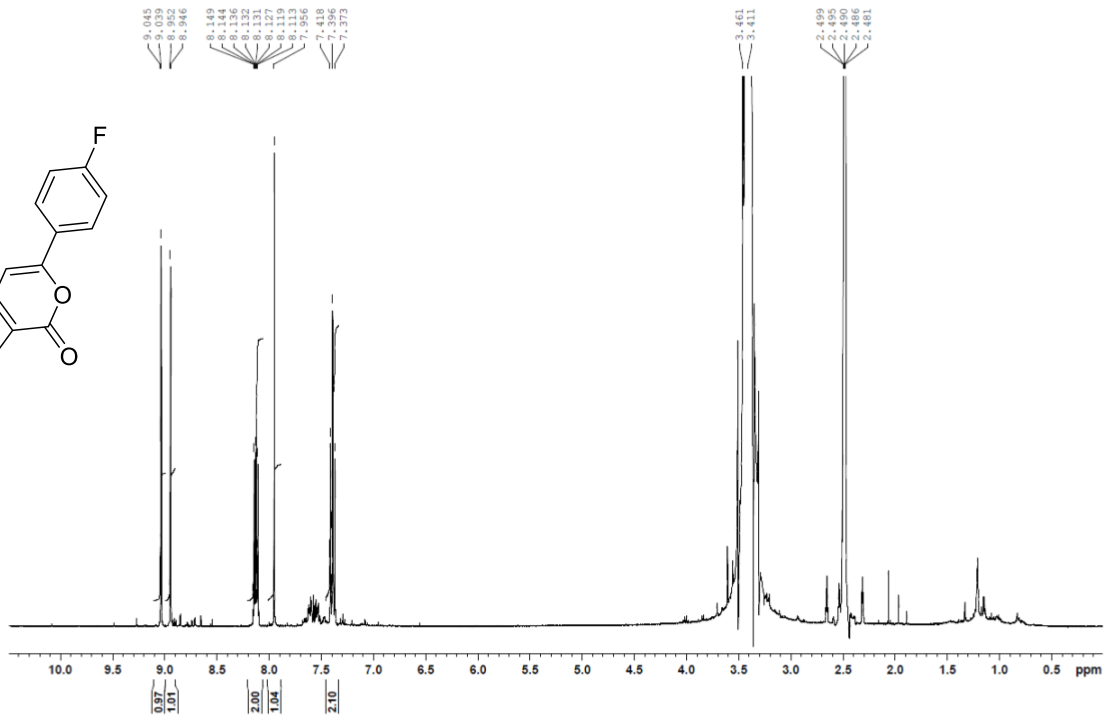

^{13}C NMR (DMSO-*d*6, 100.6 MHz) of compound **3c**

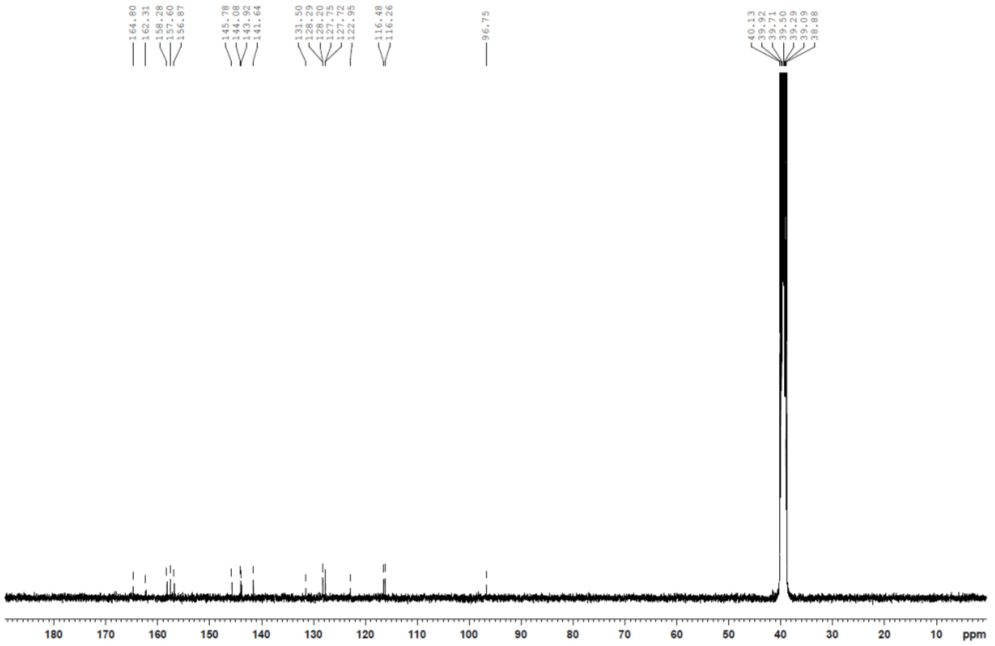

^{19}F NMR (DMSO-d_6, 282.85 MHz) of compound **3c**

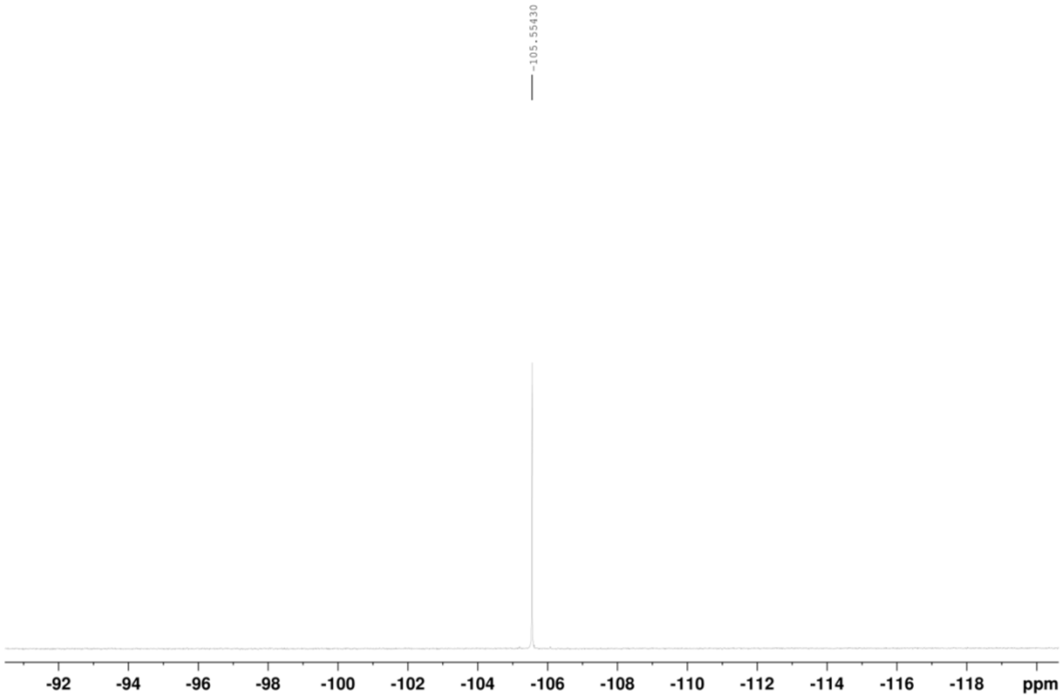

HPLC chromatogram of compound **3c**.

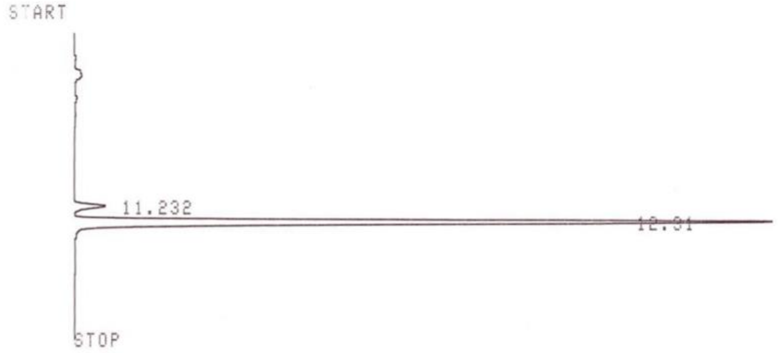

CHROMATOPAC C-R6A FILE 0
SAMPLE NO 0 METHOD 41
REPORT NO 2511

PKNO	TIME	AREA	MK	IDNO	CONC	NAME
1	11.232	7015			4.0906	
2	12.31	164465			95.9094	
	TOTAL	171480			100	

13. NMR spectra of compound 3d

^{1}H NMR (DMSO-d_6, 400 MHz) of compound **3d**

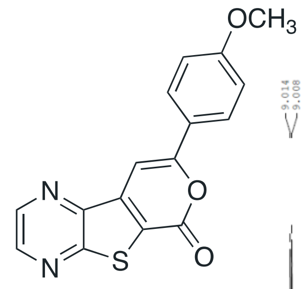

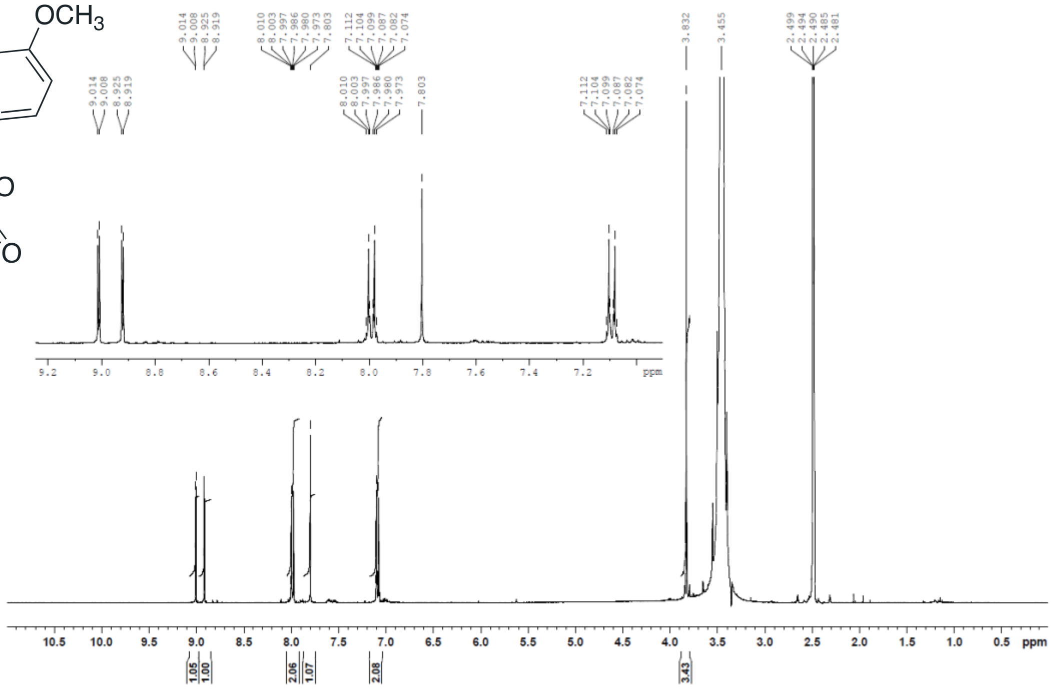

^{13}C NMR (DMSO-d_6, 100.6 MHz) of compound **3d**

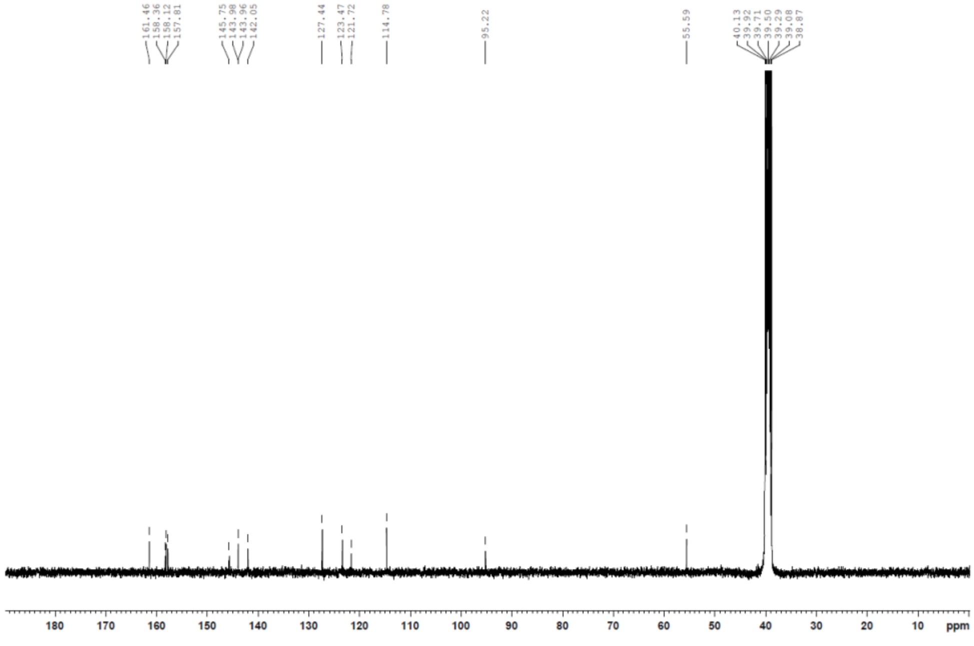

HPLC chromatogram of compound **3d**.

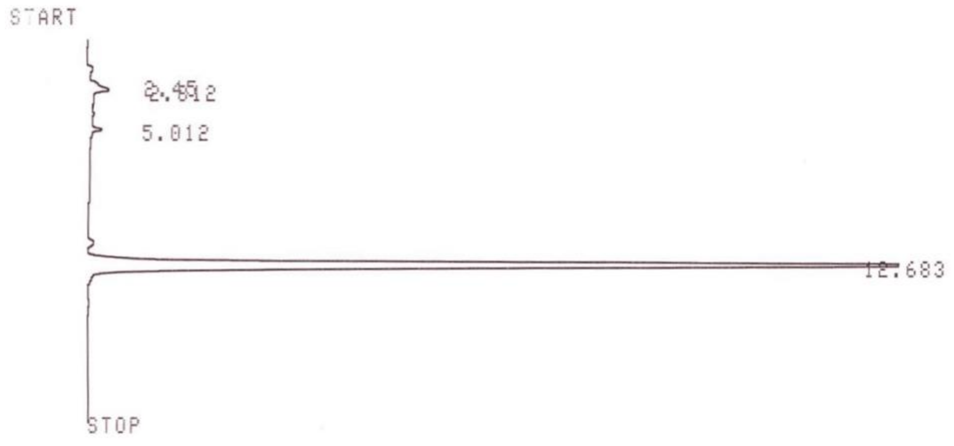

CHROMATOPAC C-R6A FILE 0
SAMPLE NO 0 METHOD 41
REPORT NO 2518

PKNO	TIME	AREA	MK	IDNO	CONC	NAME
1	2.45	1097			0.5185	
2	2.812	3107	V		1.4688	
3	5.012	1054			0.4982	
4	12.683	206284			97.5145	
	TOTAL	211542			100	

14. NMR spectra of compound 3e

^{1}H NMR (DMSO-*d*$_6$, 400 MHz) of compound **3e**

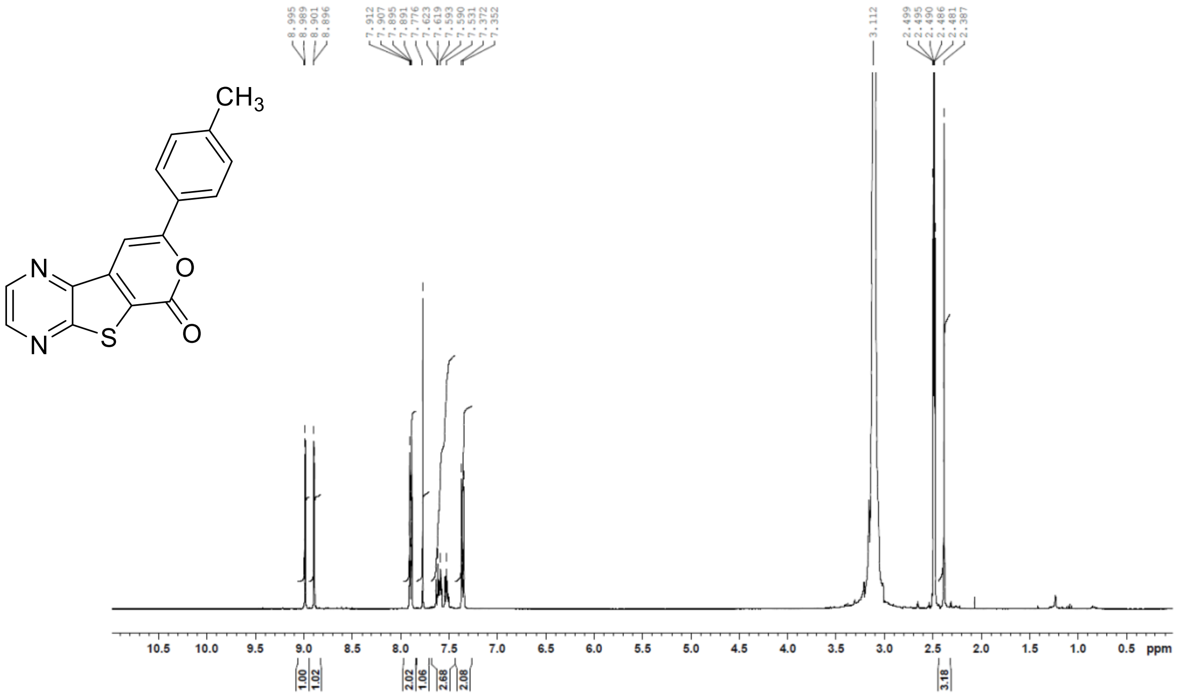

^{13}C NMR (DMSO-*d*$_6$, 100.6 MHz) of compound **3e**

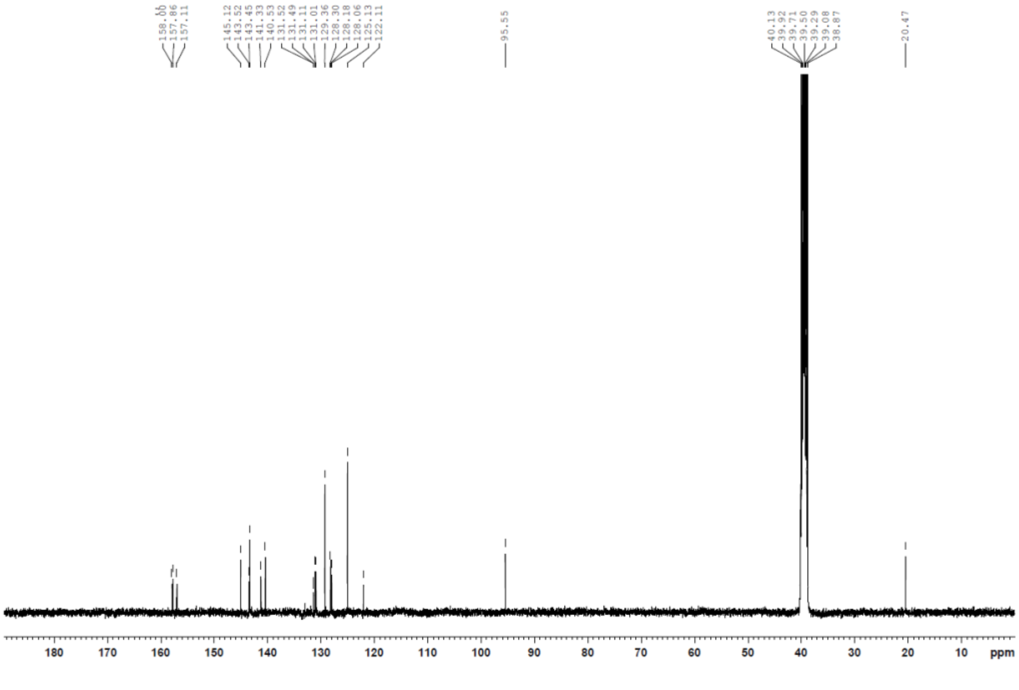

HPLC chromatogram of compound **3e**.

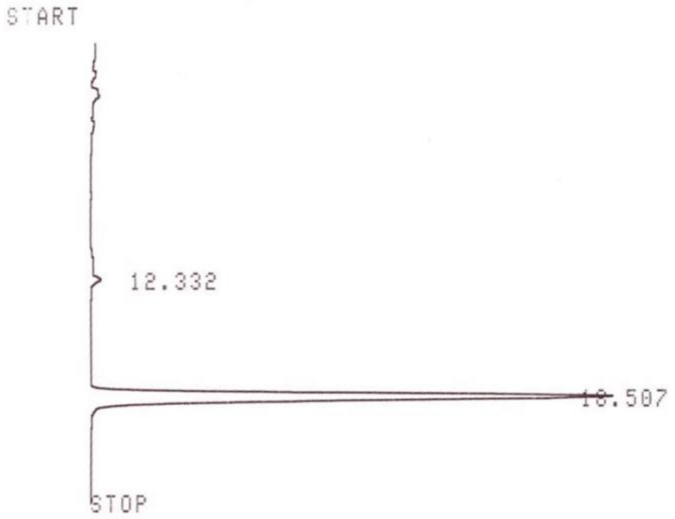

CHROMATOPAC C-R6A FILE 0
SAMPLE NO 0 METHOD 41
REPORT NO 2517

PKNO	TIME	AREA	MK	IDNO	CONC	NAME
1	12.332	1422			1.0963	
2	18.507	128256			98.9037	
	TOTAL	129678			100	

15. NMR spectra of compound 3f

^{1}H NMR (DMSO-d_6, 400 MHz) of compound **3f**

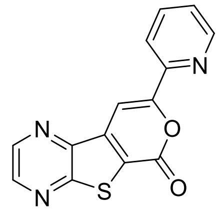

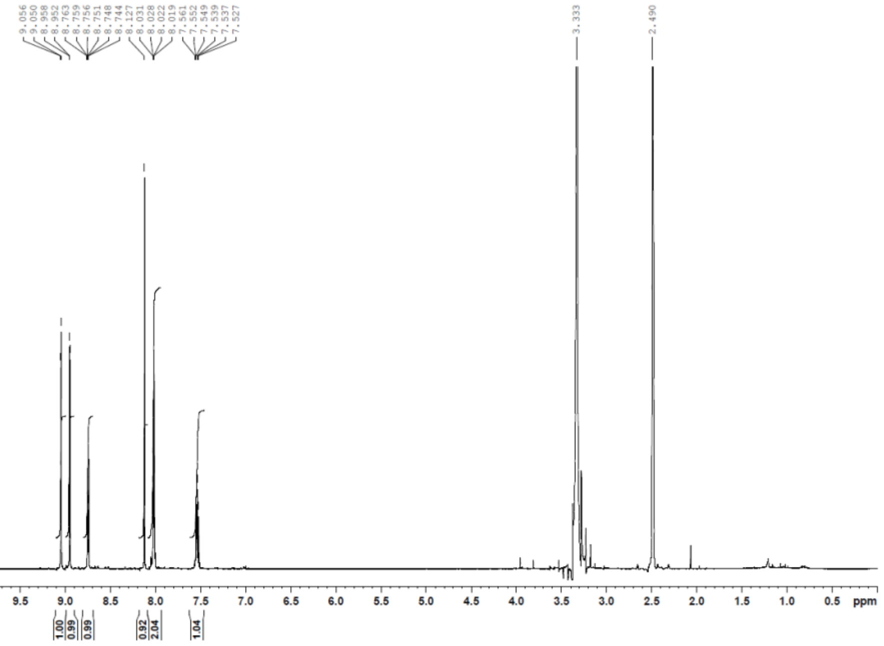

^{13}C NMR (DMSO-d_6, 100.6 MHz) of compound **3f**

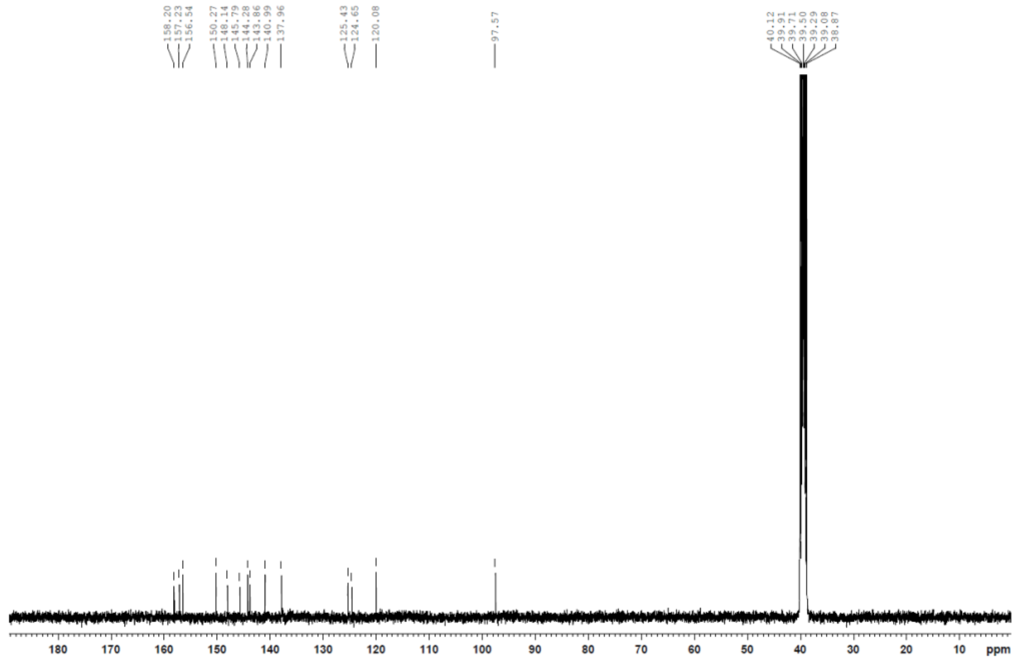

16. NMR spectra of compound 3g

^{1}H NMR (DMSO-d_6, 400 MHz) of compound **3g**

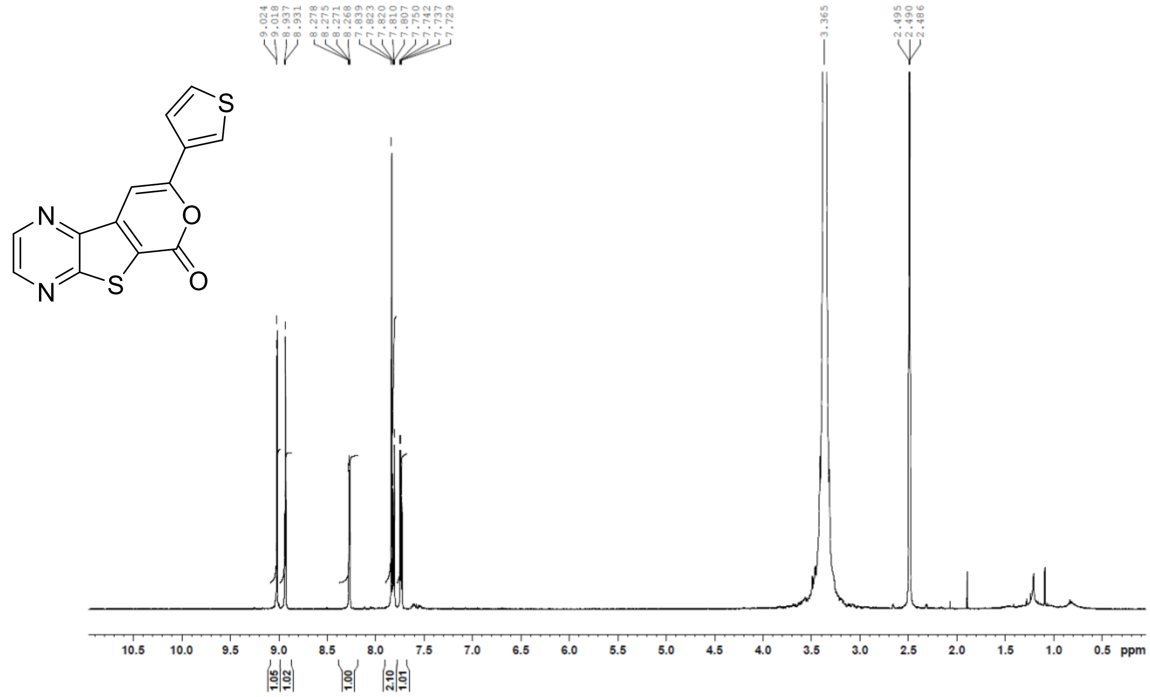

^{13}C NMR (DMSO-d_6, 100.6 MHz) of compound **3g**

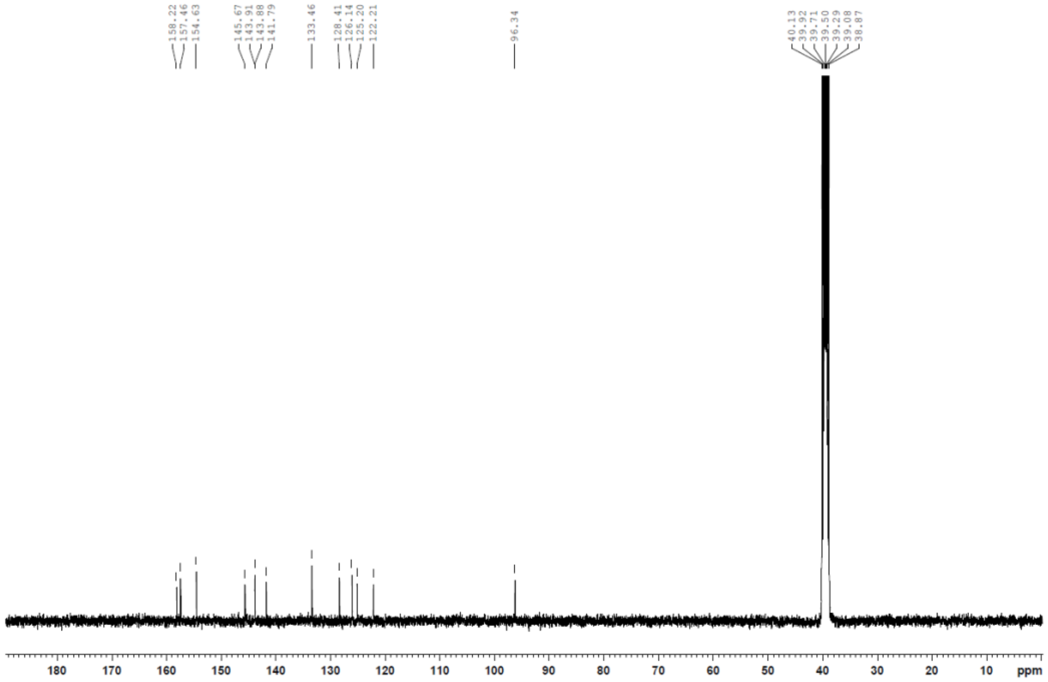

17. NMR spectra of compound 4a

^{1}H NMR (DMSO-d_6, 400 MHz) of compound **4a**

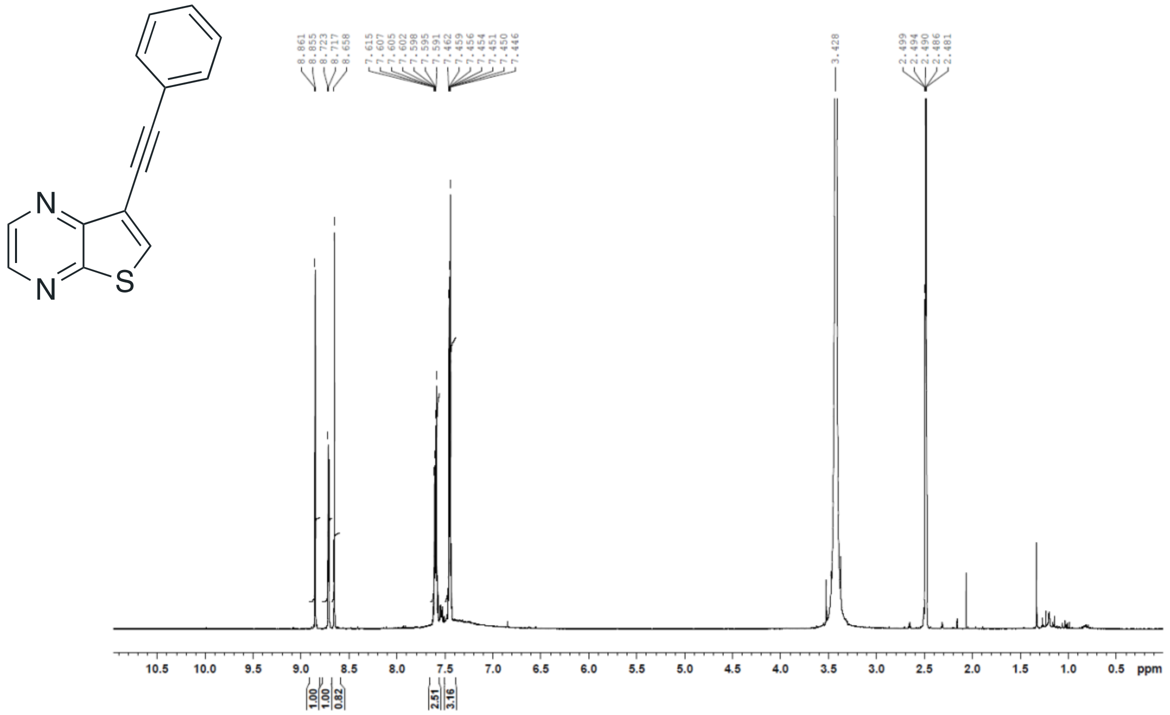

^{13}C NMR (DMSO-d_6, 100.6 MHz) of compound **4a**

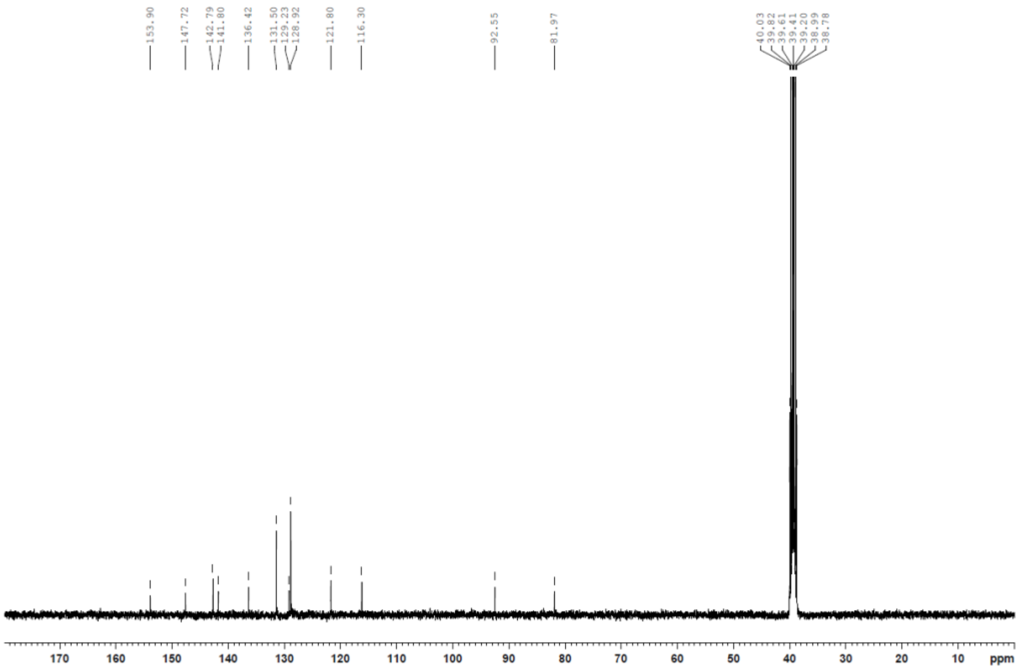

18. NMR spectra of compound 4c

^{1}H NMR (DMSO-d_6, 400 MHz) of compound **4c**

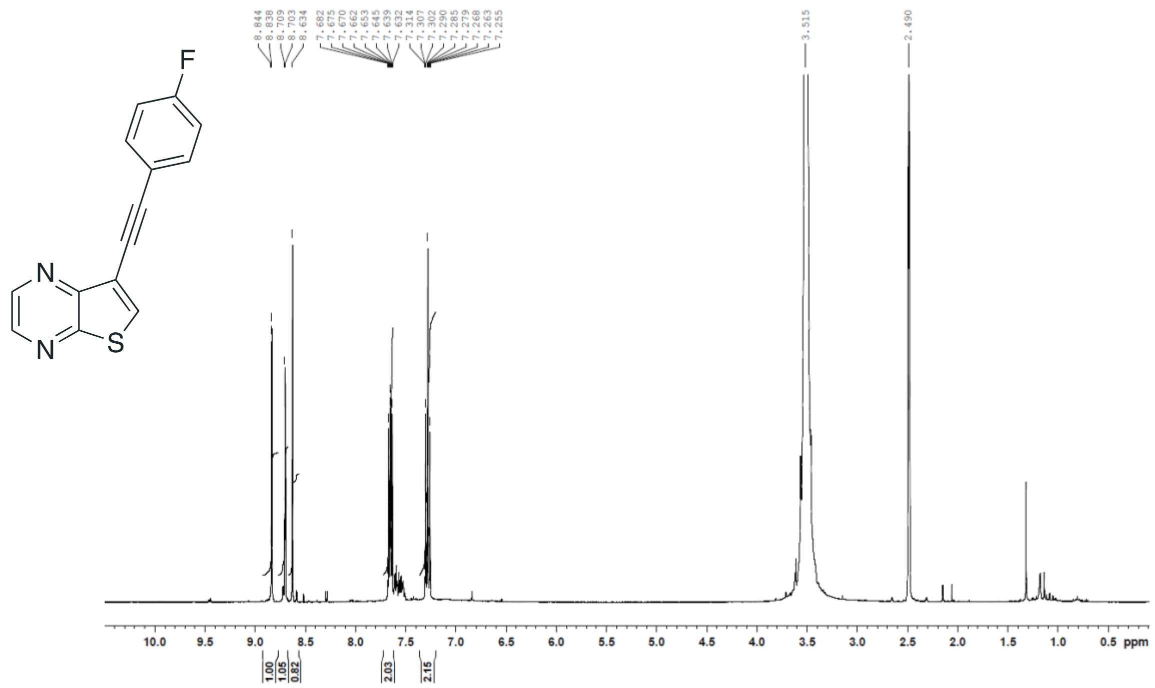

^{13}C NMR (DMSO-d_6, 100.6 MHz) of compound **4c**

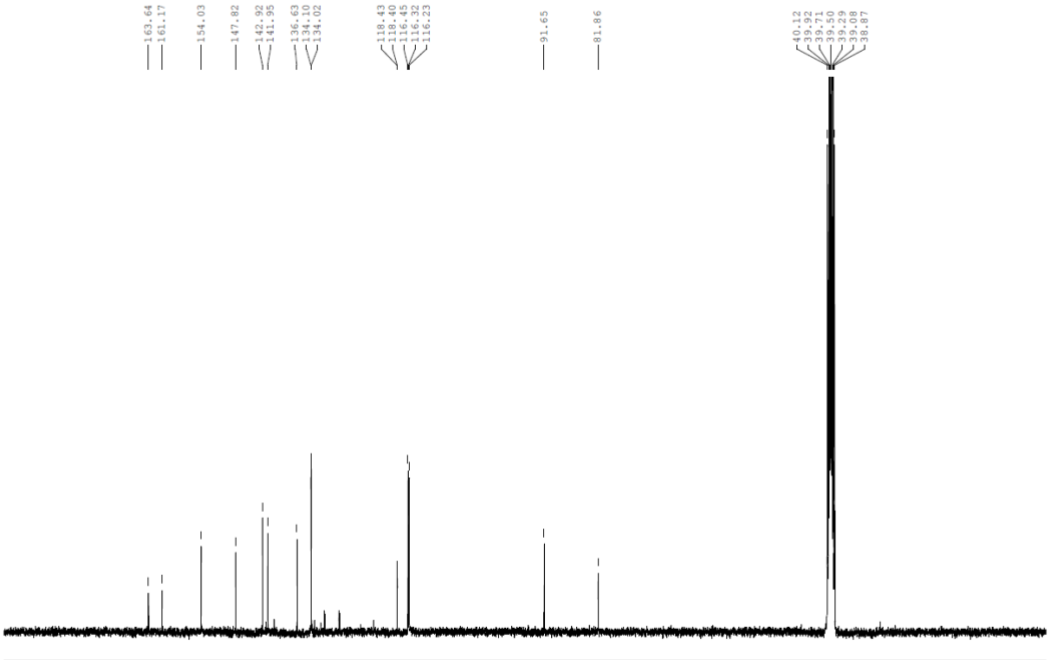

^{19}F NMR (DMSO-d_6, 282.85 MHz) of compound **4c**

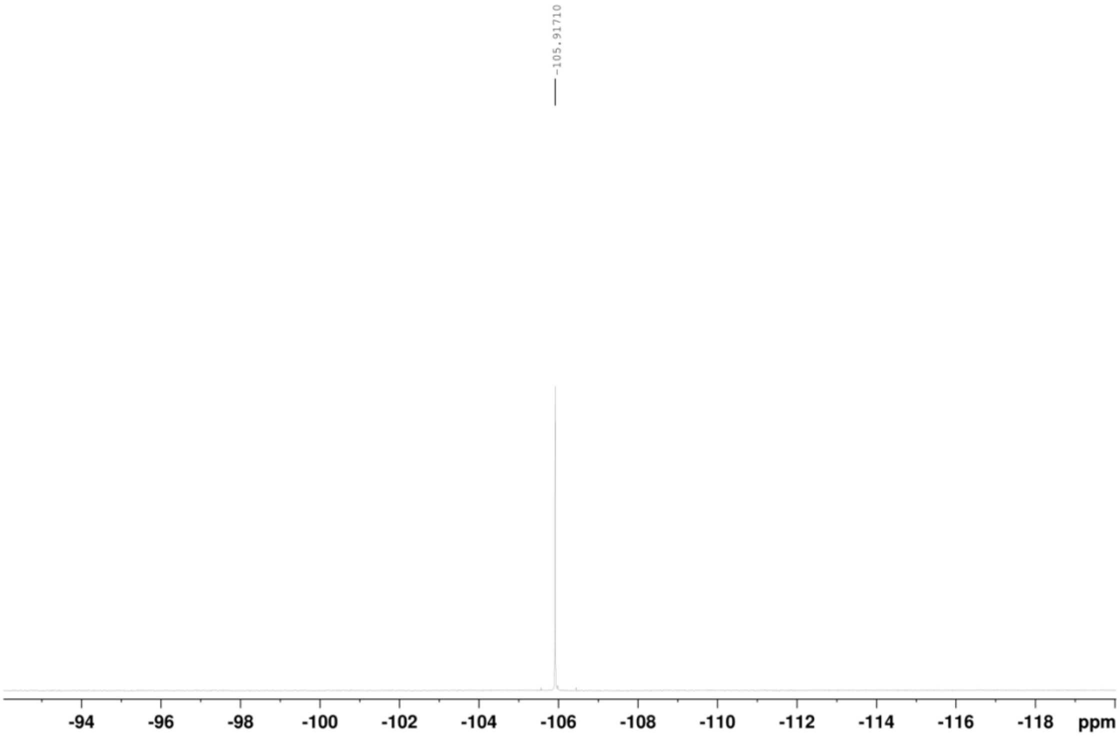

19. NMR spectra of compound 4d

^{1}H NMR (DMSO-d_6, 400 MHz) of compound **4d**

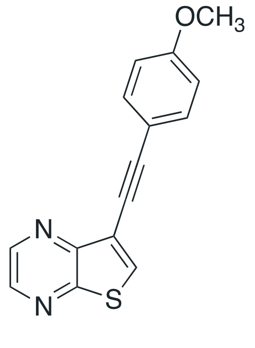

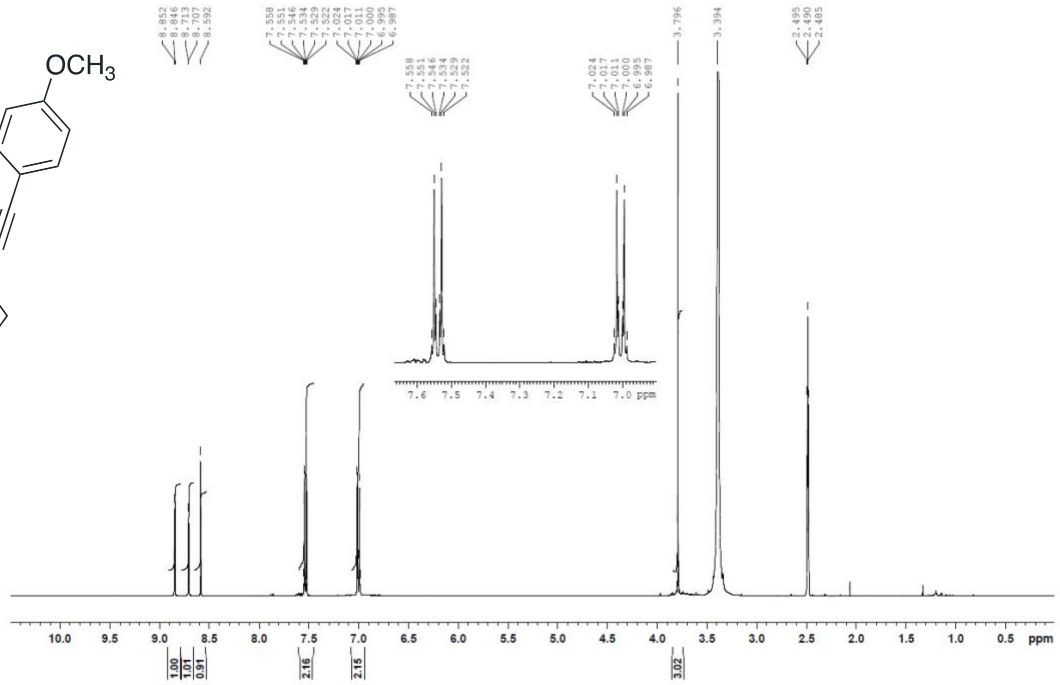

^{13}C NMR (DMSO-d_6, 100.6 MHz) of compound **4d**

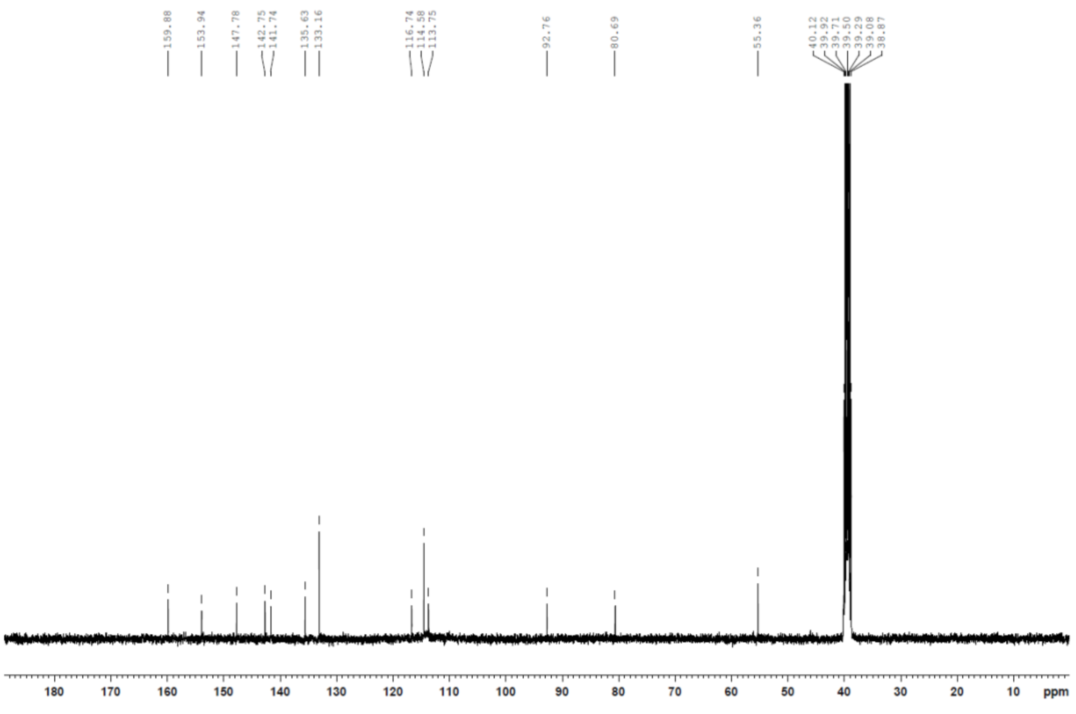

20. NMR spectra of compound 4e

^{1}H NMR (DMSO-d_6, 400 MHz) of compound **4e**

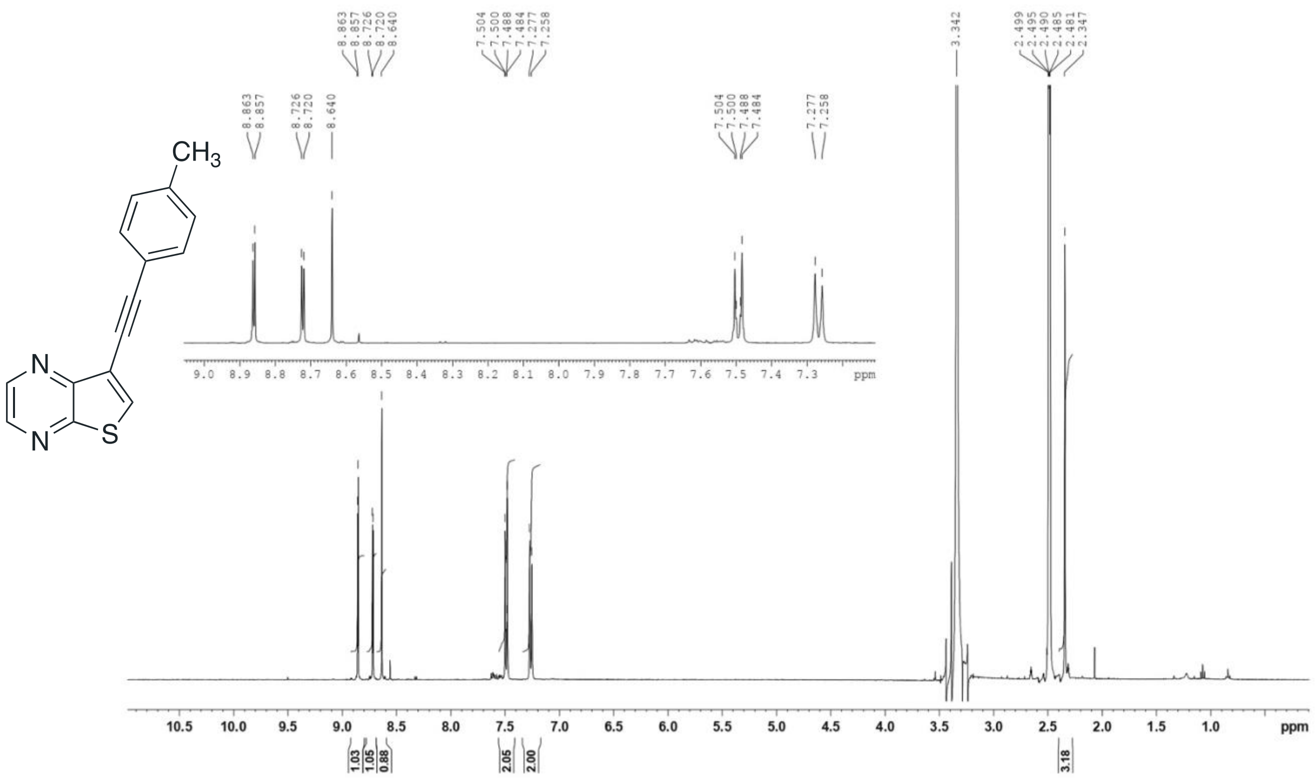

21. NMR spectra of compound 4g

^{1}H NMR (DMSO-d_6, 400 MHz) of compound **4g**

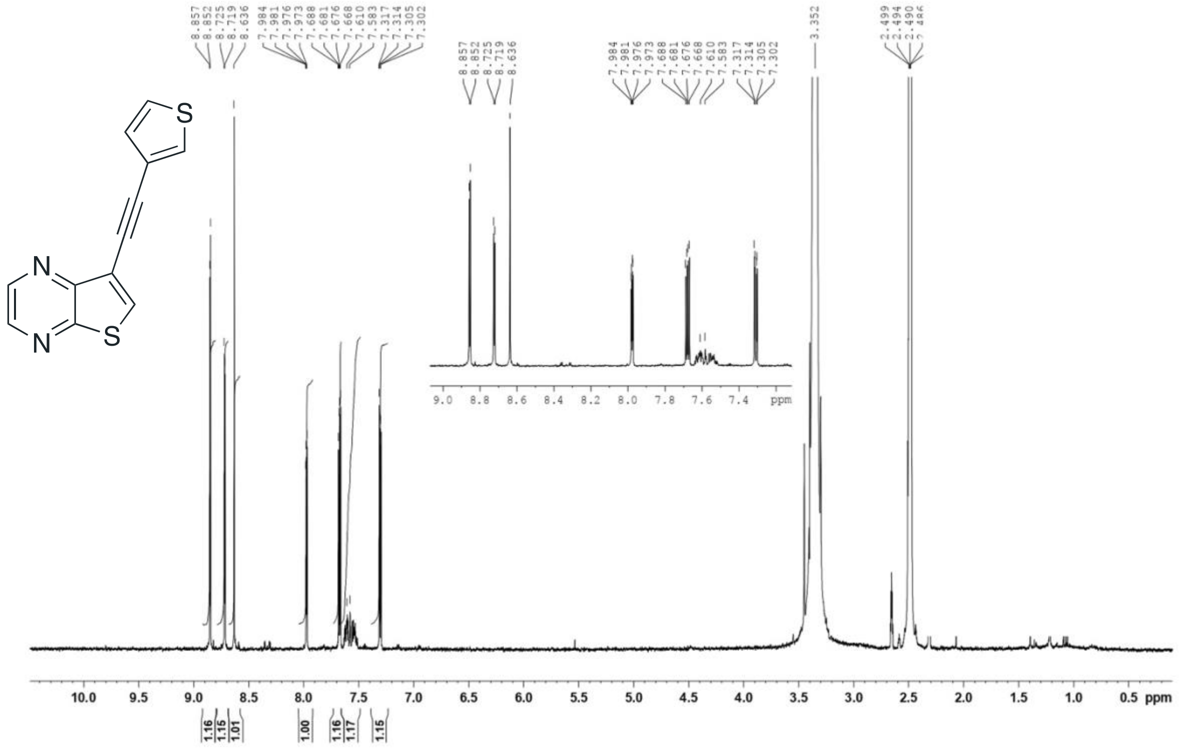

22. NMR spectra of compound 5a

^{1}H NMR (DMSO-*d₆*, 400 MHz) of compound **5a**

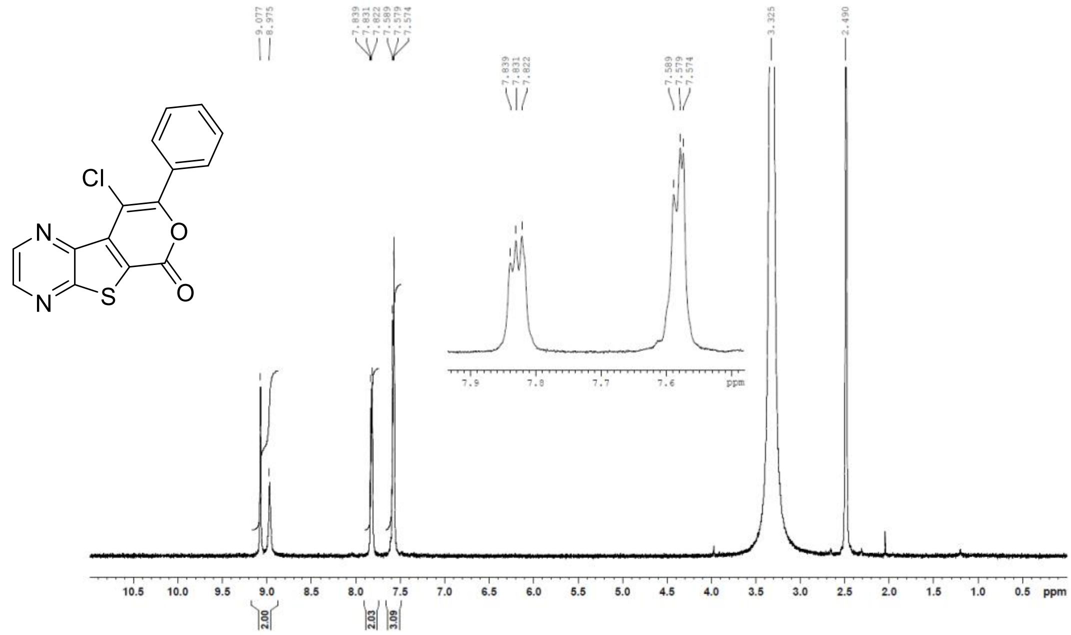

^{13}C NMR (DMSO-*d₆*, 100.6 MHz) of compound **5a**

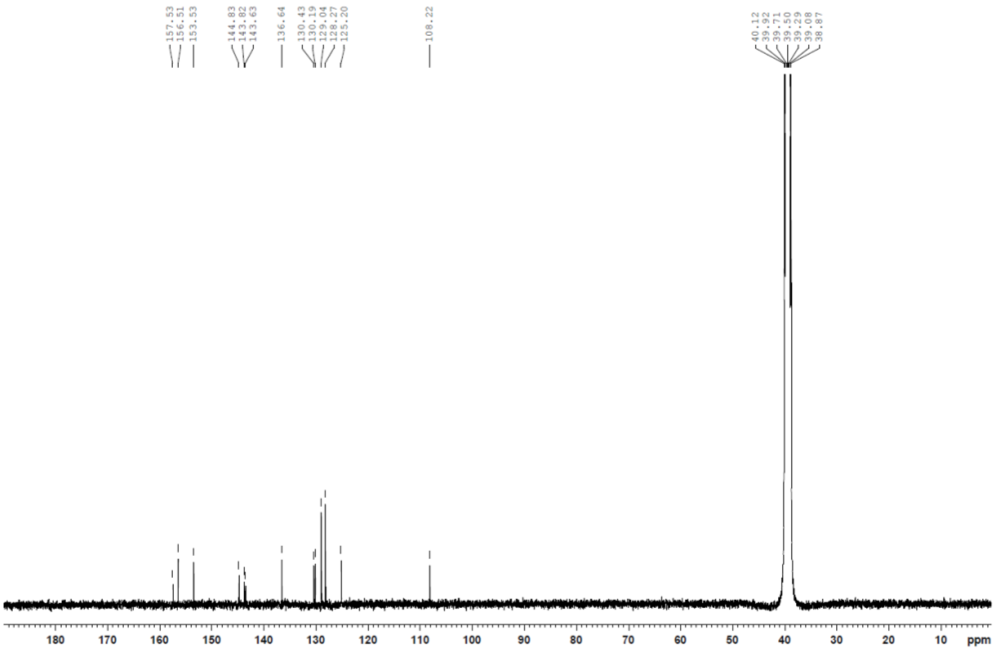

23. NMR spectra of compound 5b

^{1}H NMR (DMSO-d_6, 400 MHz) of compound **5b**

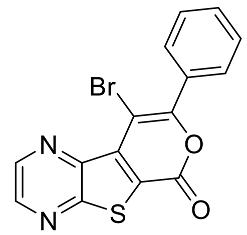

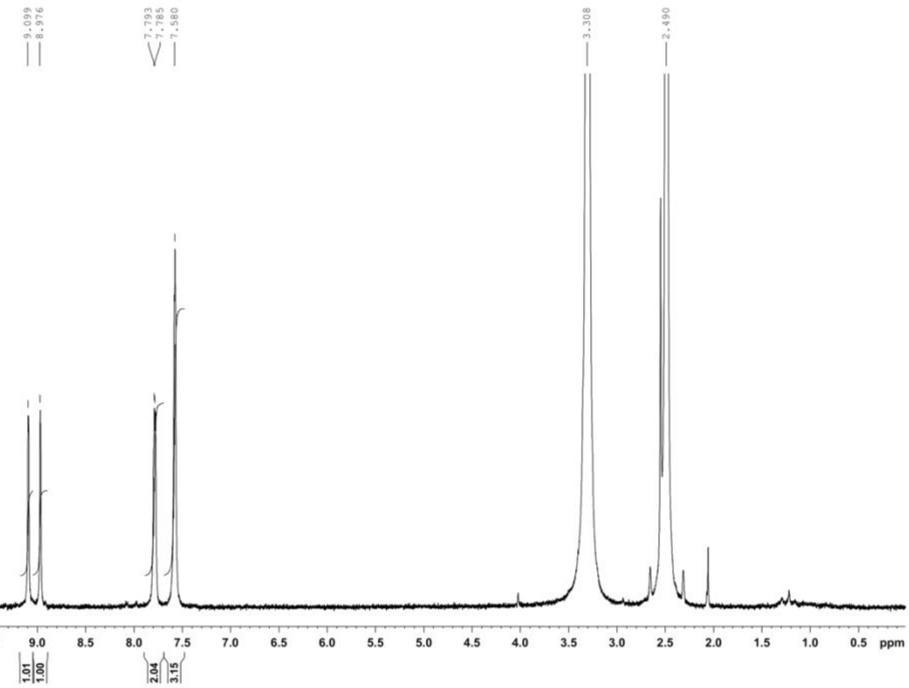

^{13}C NMR (DMSO-d_6, 100.6 MHz) of compound **5b**

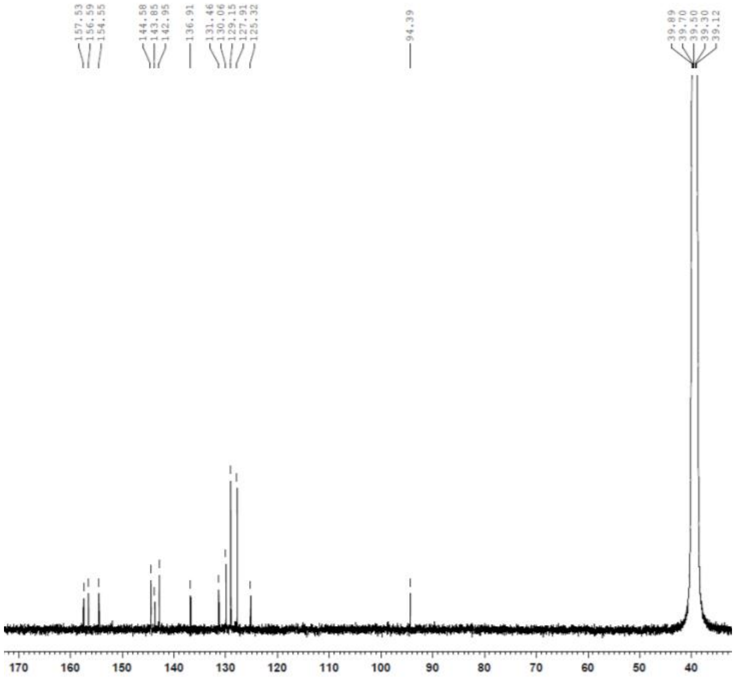

24. NMR spectra of compound 7a

^{1}H NMR (DMSO-d_6, 400 MHz, 80 °C) of compound **7a**

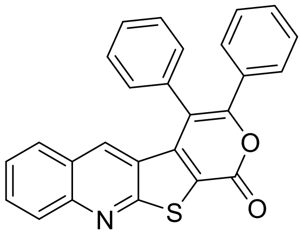

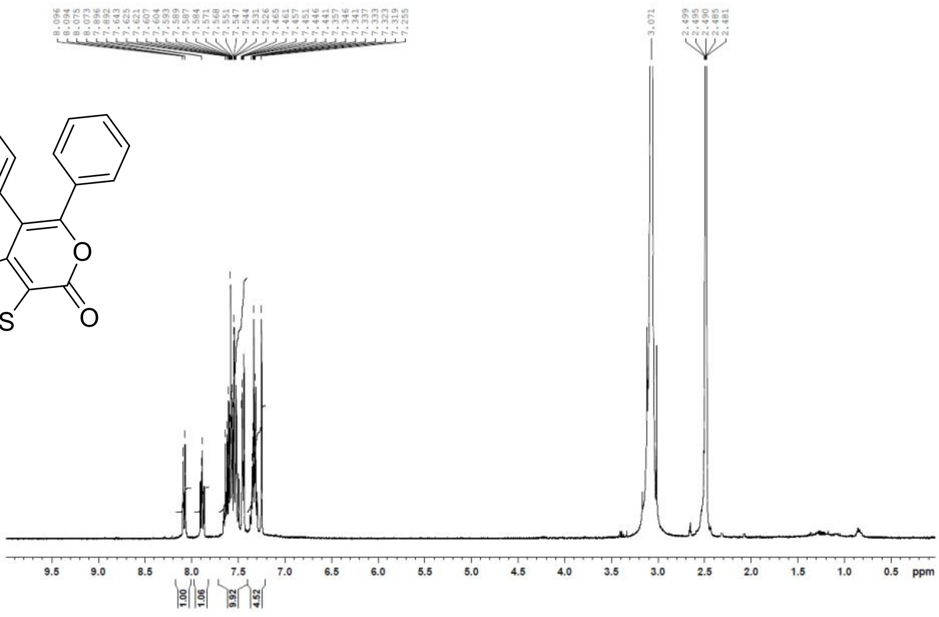

^{13}C NMR (DMSO-d_6, 100.6 MHz, 80 °C) of compound **7a**

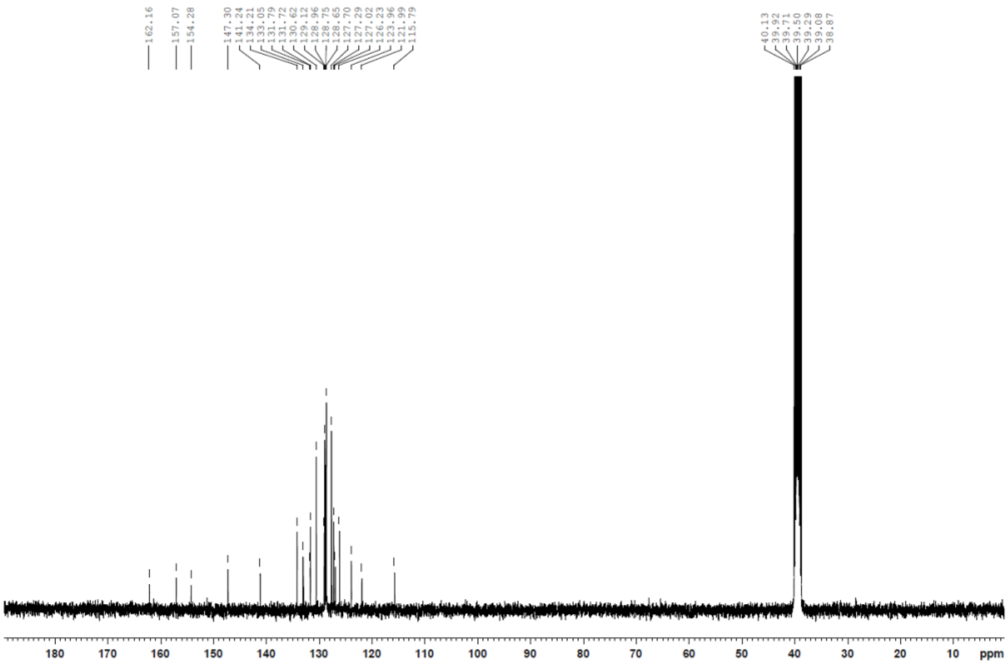

Aromatic expansion of ^1H-^{13}C bidimensional correlations - HSQC spectrum of compound **7a**

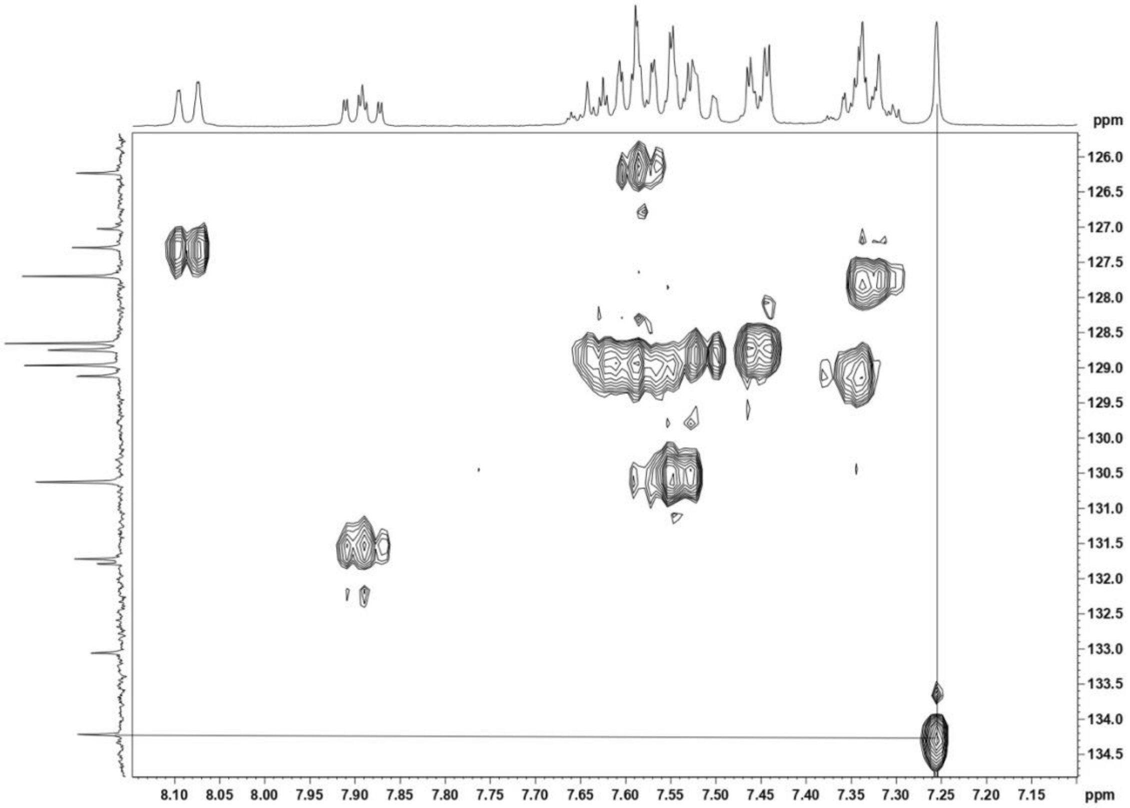

25. NMR spectra of compound 7b

^{1}H NMR (DMSO-*d6,* 400 MHz, 80 °C) of compound **7b**

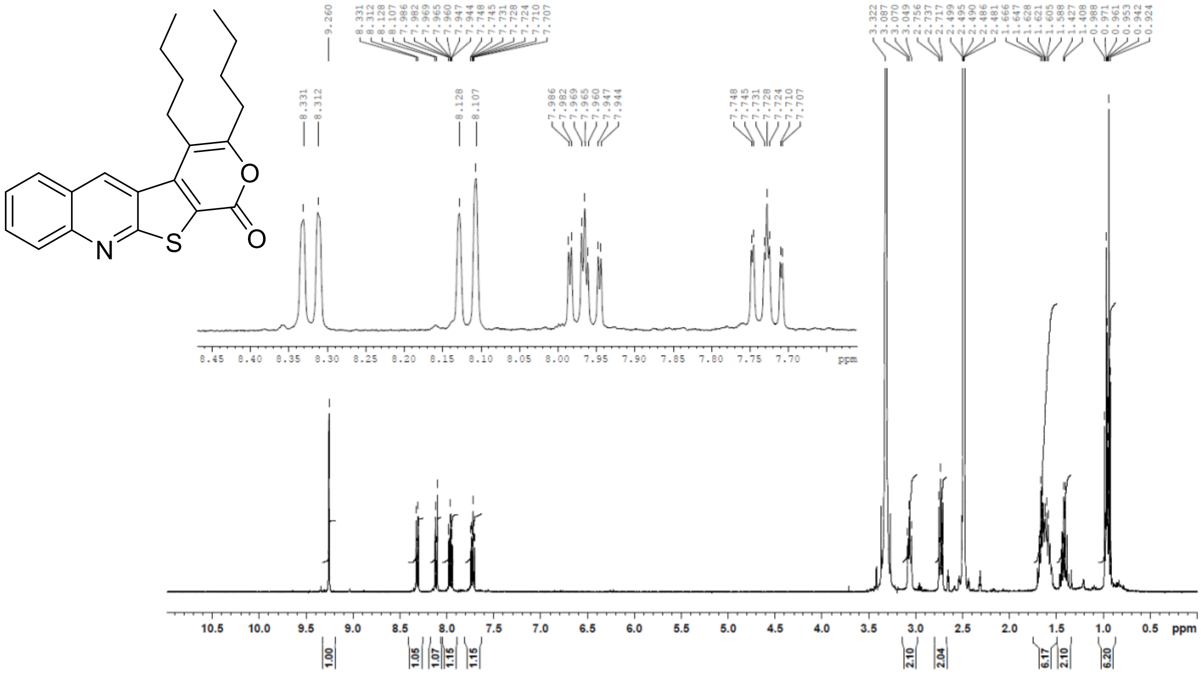

^{13}C NMR (DMSO-*d6*, 100.6 MHz, 80 °C) of compound **7b**

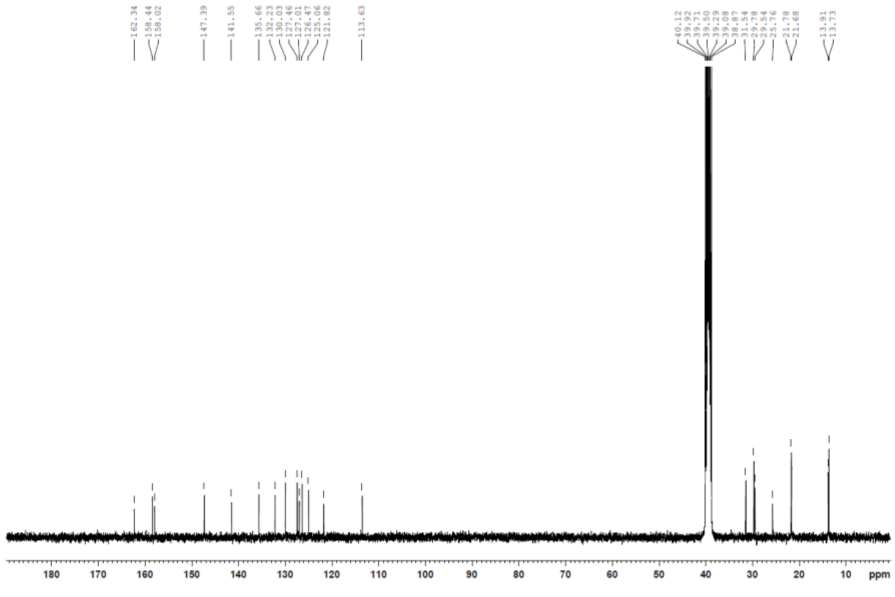

Aromatic expansion of ^{1}H-^{13}C bidimensional correlations - HSQC spectrum of compound **7b**

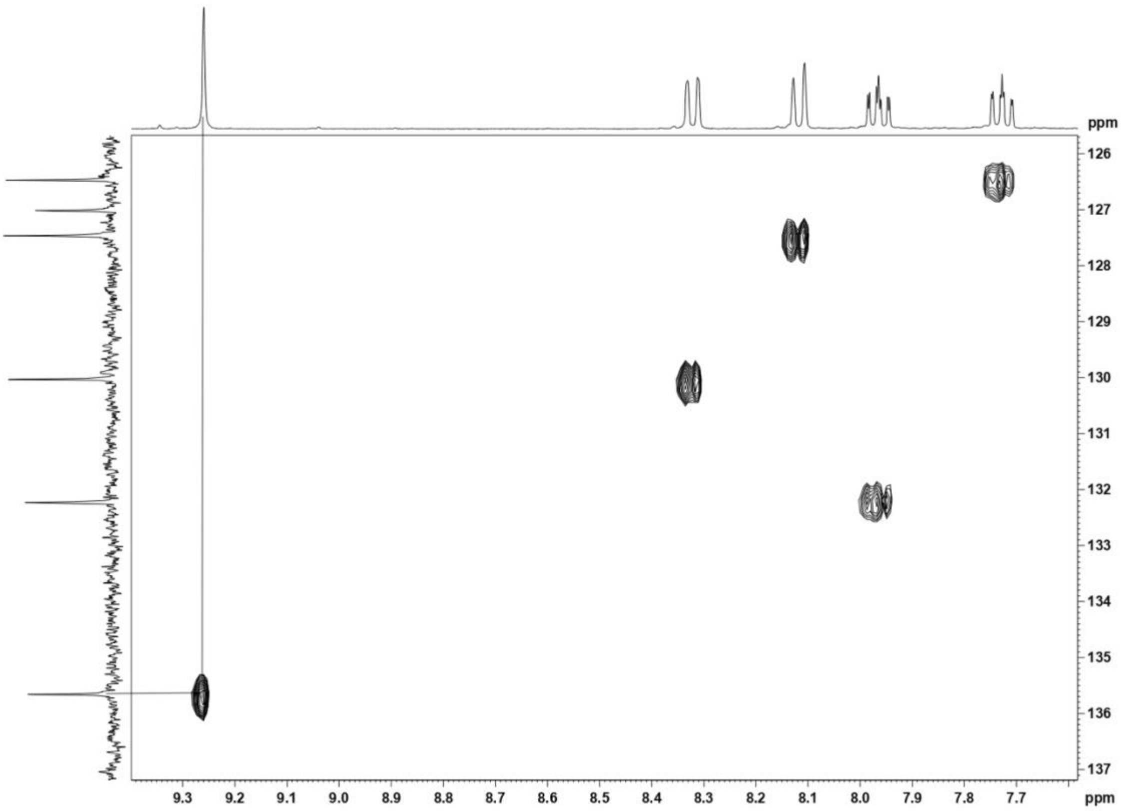

26. NMR spectra of compound 7c

^{1}H NMR (DMSO-*d6,* 400 MHz, 80 °C) of compound **7c**

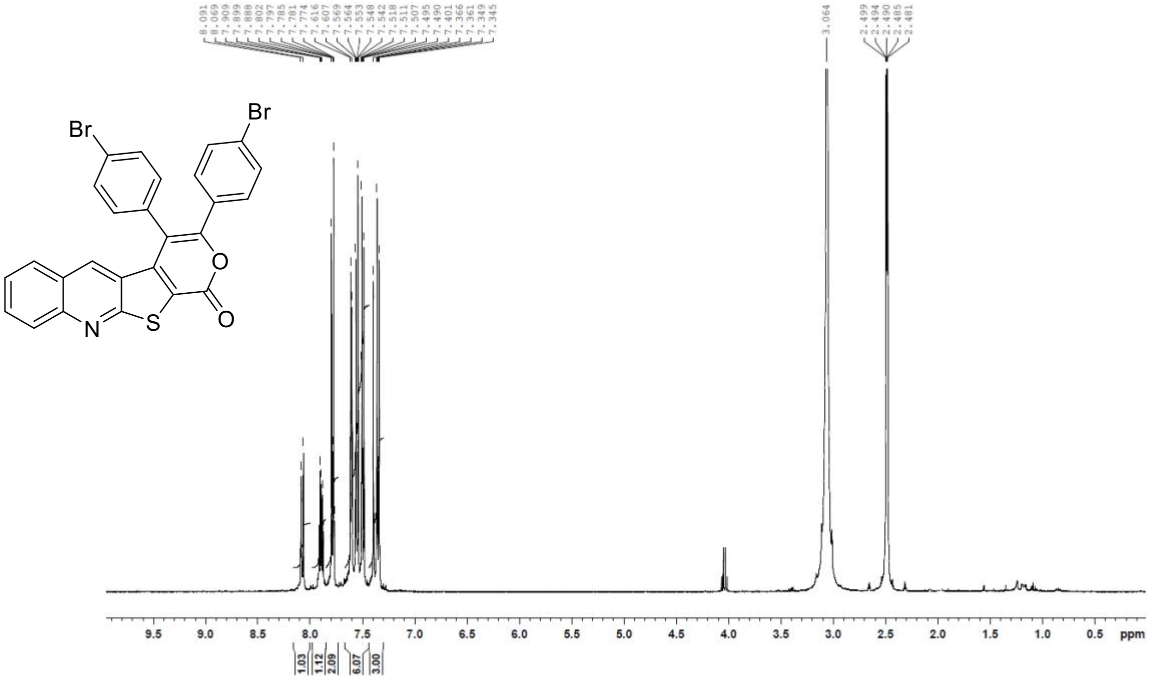

^{13}C NMR (DMSO-*d6*, 100.6 MHz, 80 °C) of compound **7c**

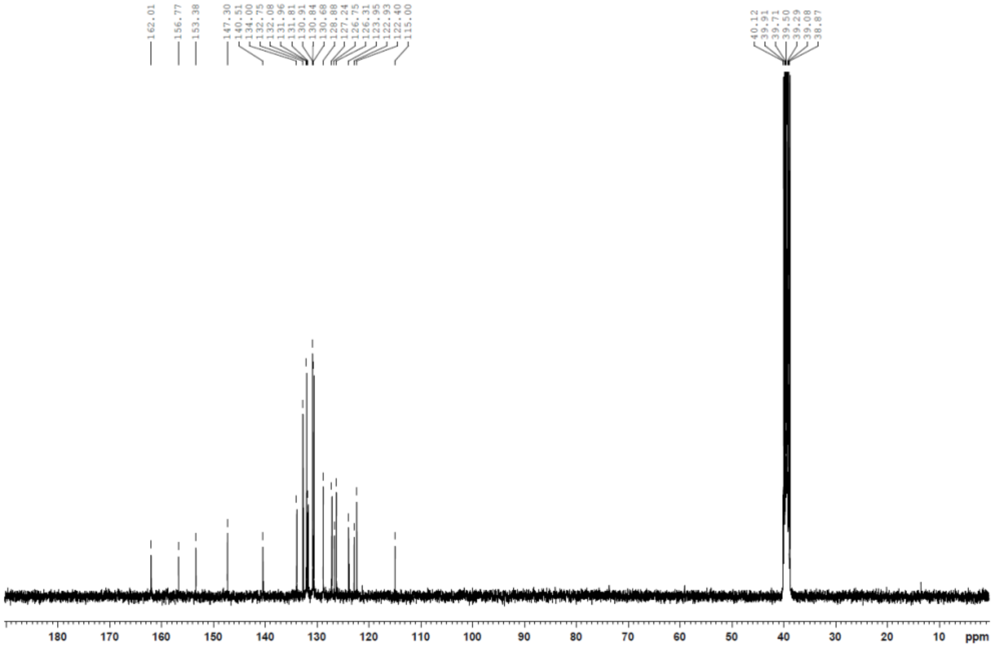

Aromatic expansion of ^{1}H-^{13}C bidimensional correlations - HSQC spectrum of compound **7c**

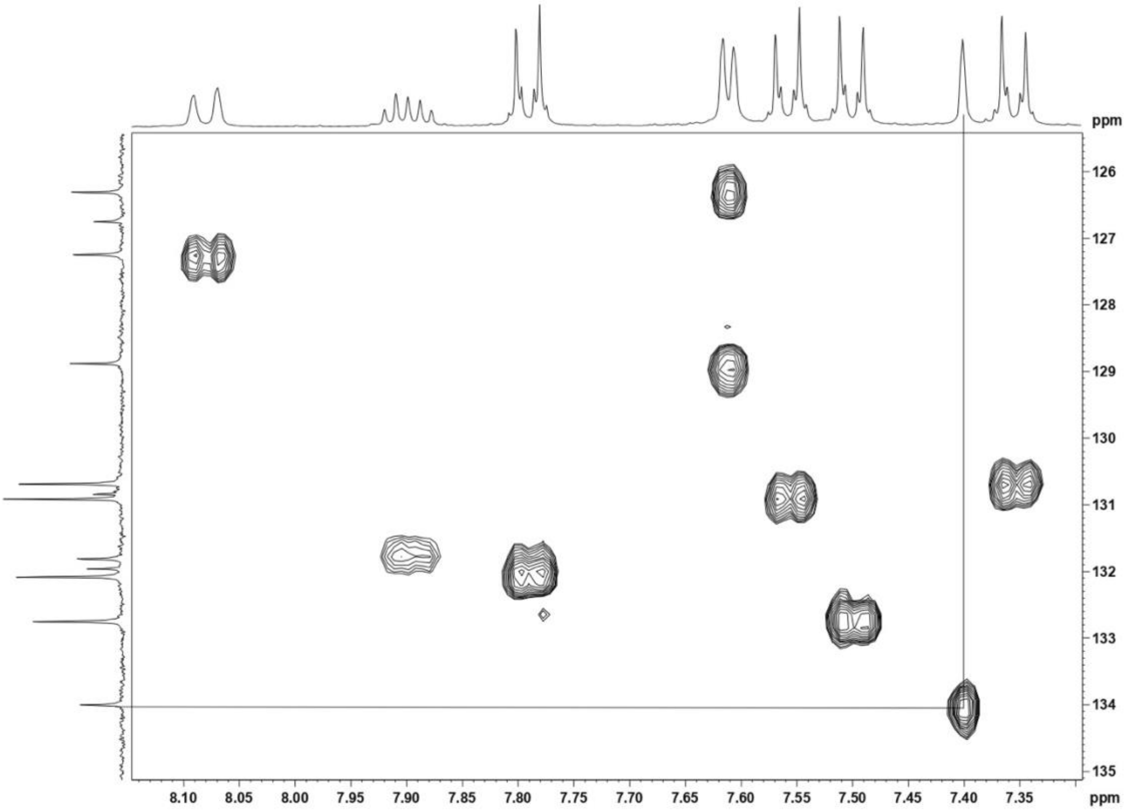

27. NMR spectra of compound 7d

^{1}H NMR (DMSO-*d*$_6$, 400 MHz, 100 °C) of compound **7d**

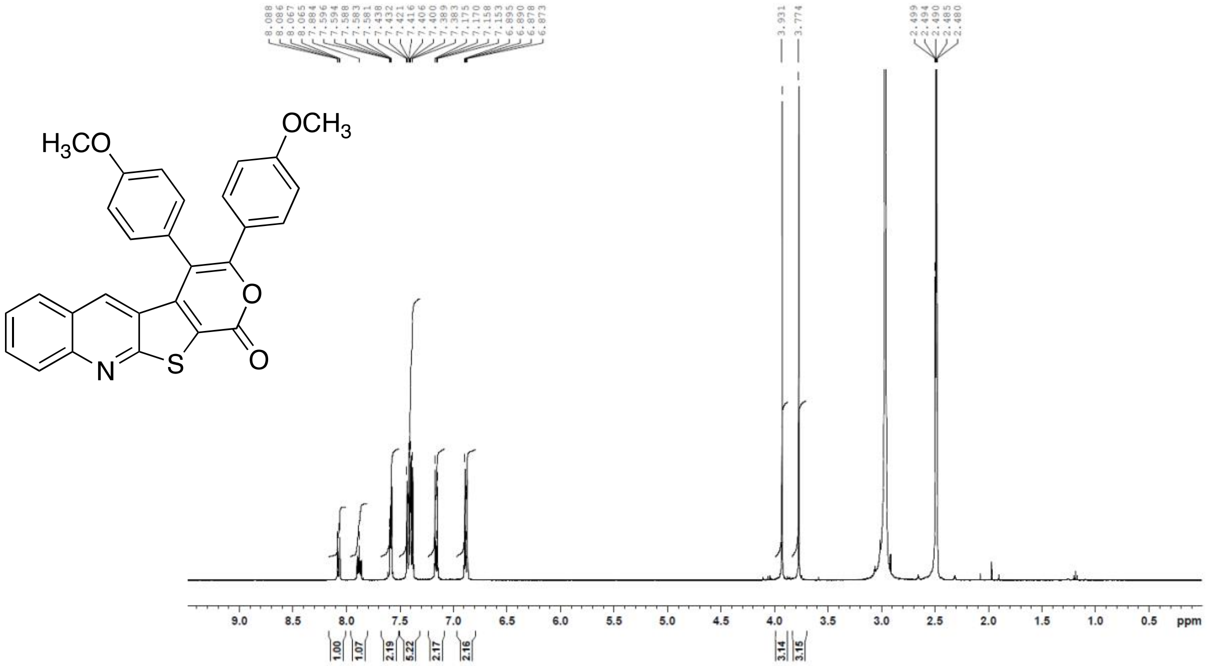

^{13}C NMR (DMSO-*d*$_6$, 100.6 MHz, 100 °C) of compound **7d**

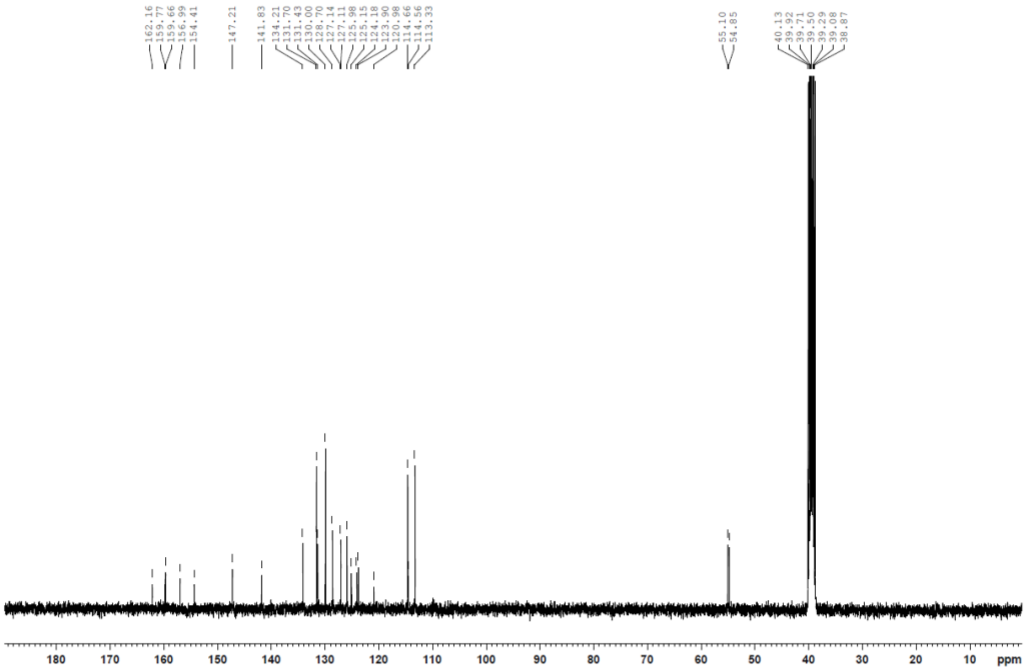

Aromatic expansion of ^{1}H-^{13}C bidimensional correlations - HSQC spectrum of compound **7d**

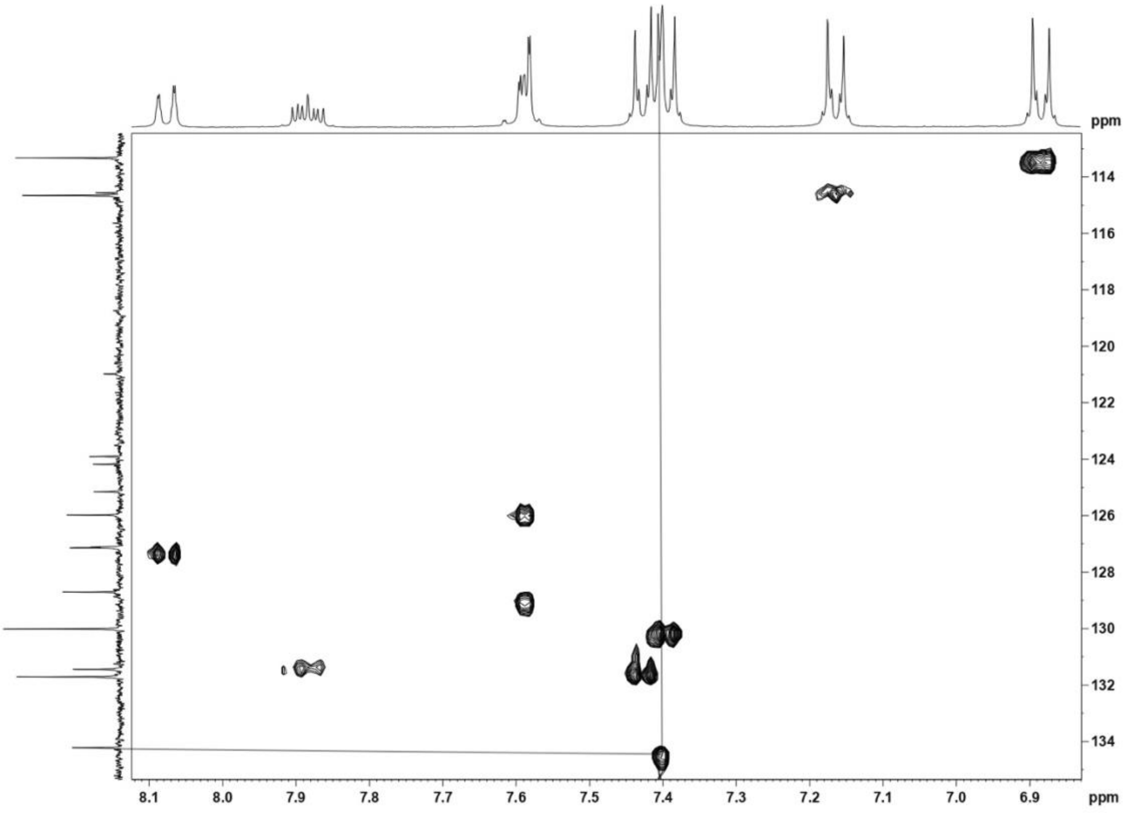

28. NMR spectra of compound 7e

^{1}H NMR (DMSO-*d*$_6$, 400 MHz, 80 °C) of compound **7e**

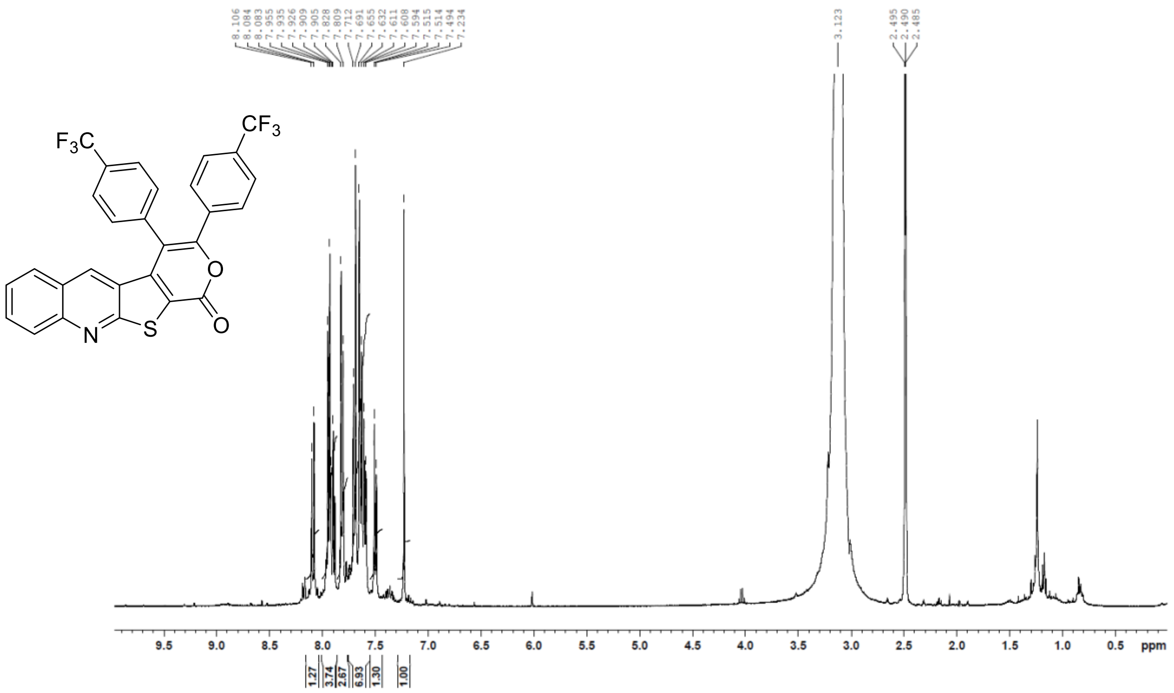

^{13}C NMR (DMSO-*d*$_6$, 100.6 MHz, 80 °C) of compound **7e**

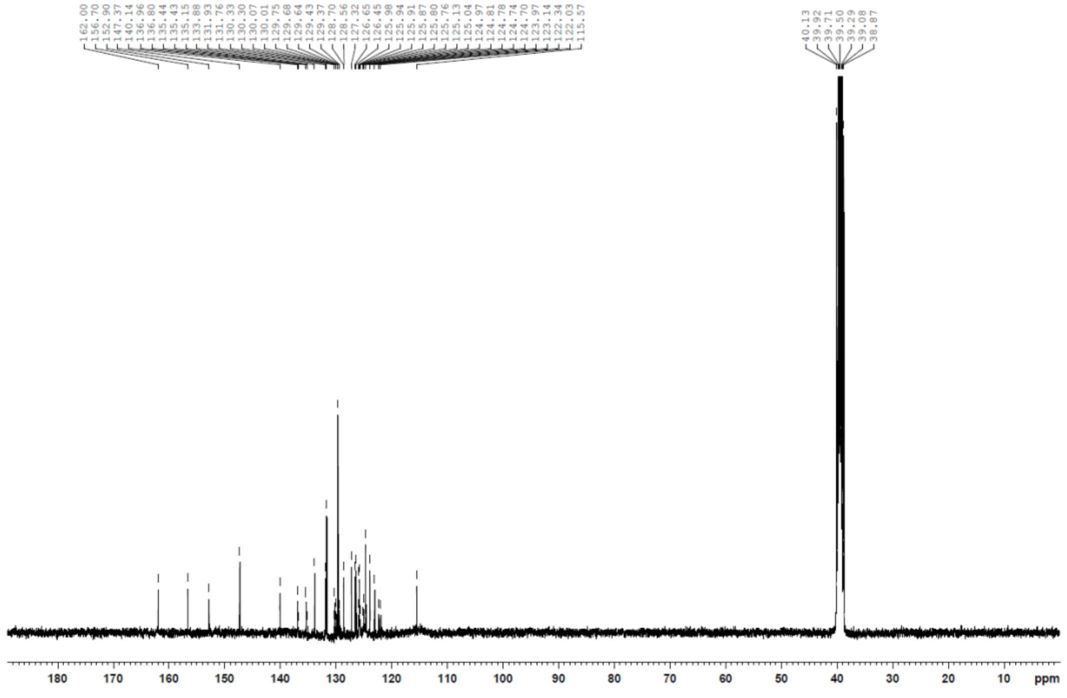

Aromatic expansion of ^{1}H-^{13}C bidimensional correlations - HSQC spectrum of compound **7e**

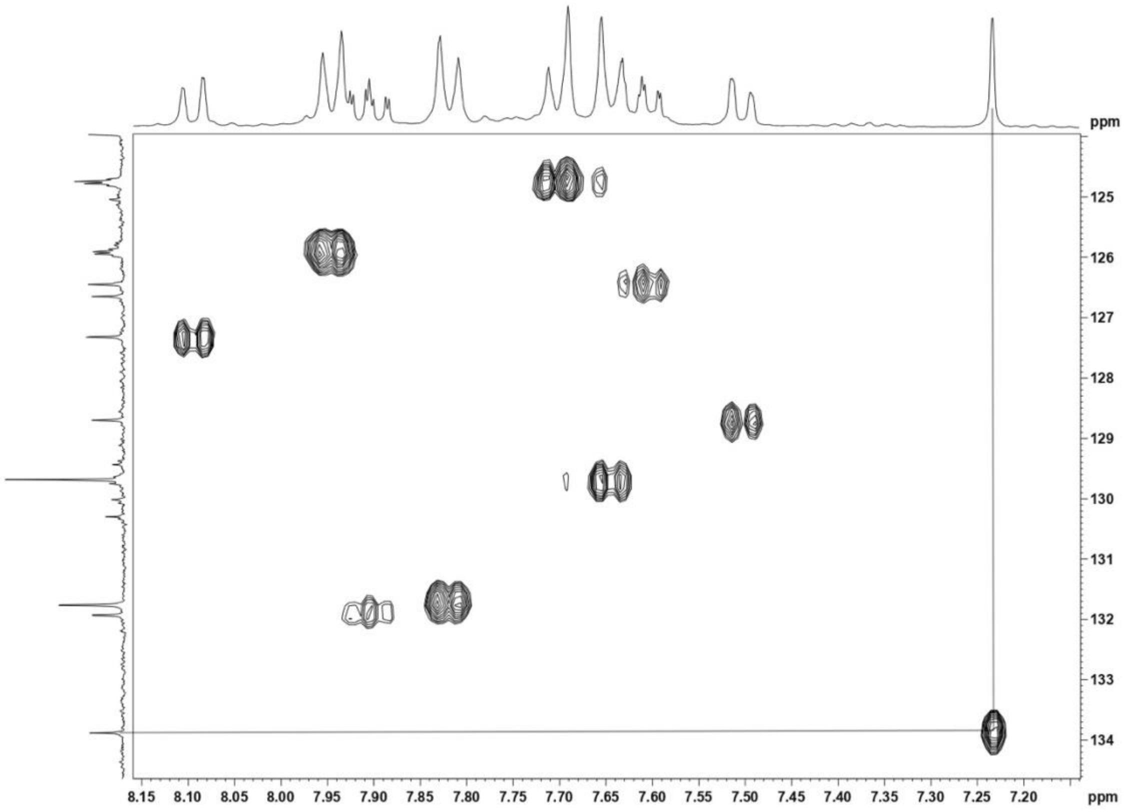

^{19}F NMR (DMSO-d_6, 282.85 MHz) of compound **7e**

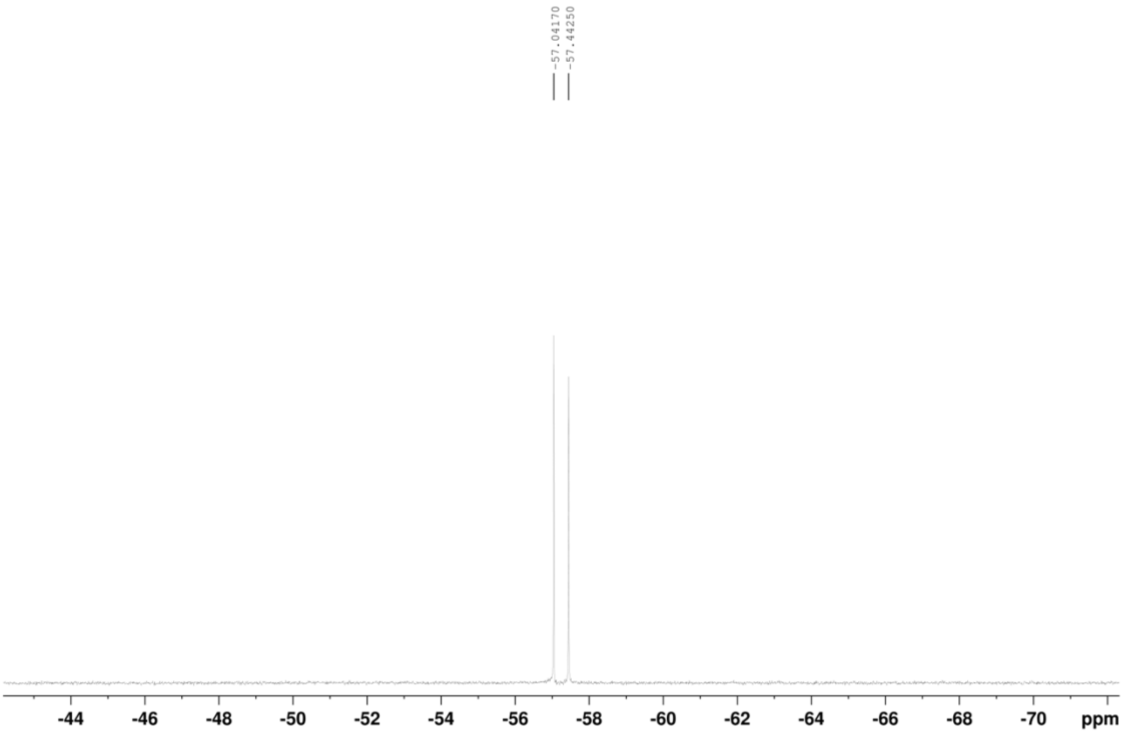

HPLC chromatogram of compound **7e**

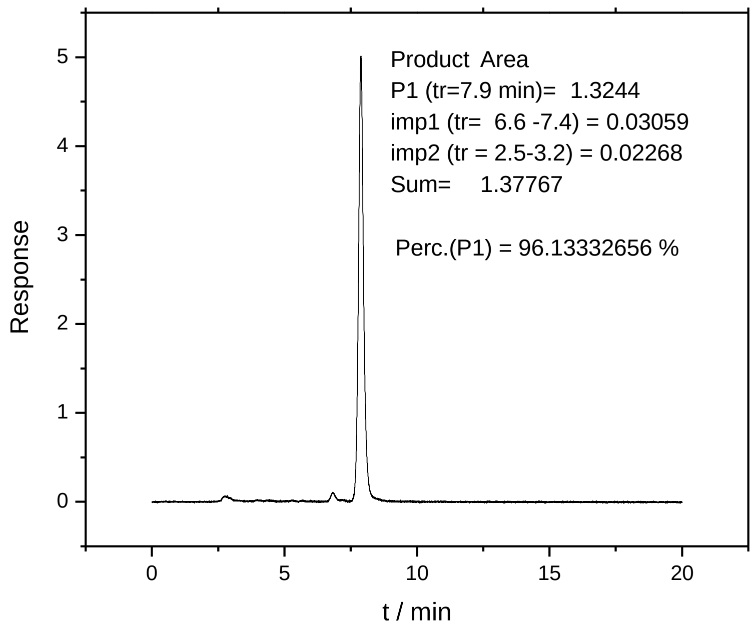

29. NMR spectra of compound 7f

^{1}H NMR (DMSO-*d*$_6$, 400 MHz, 80 °C) of compound **7f**

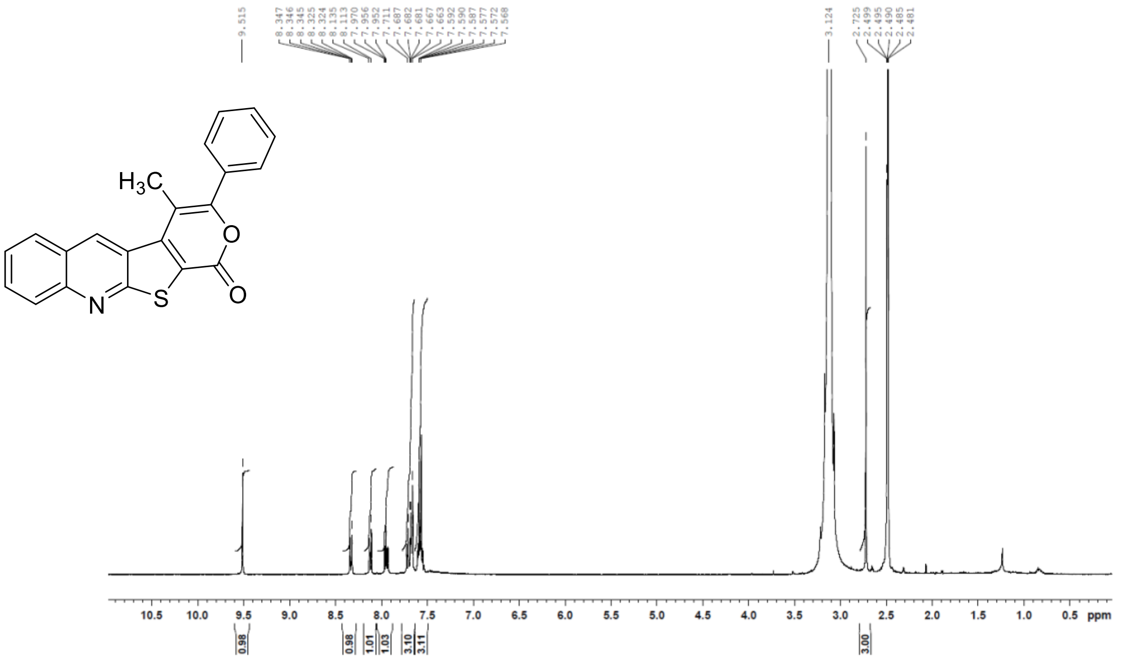

^{13}C NMR (DMSO-*d*$_6$, 100.6 MHz, 80 °C) of compound **7f**

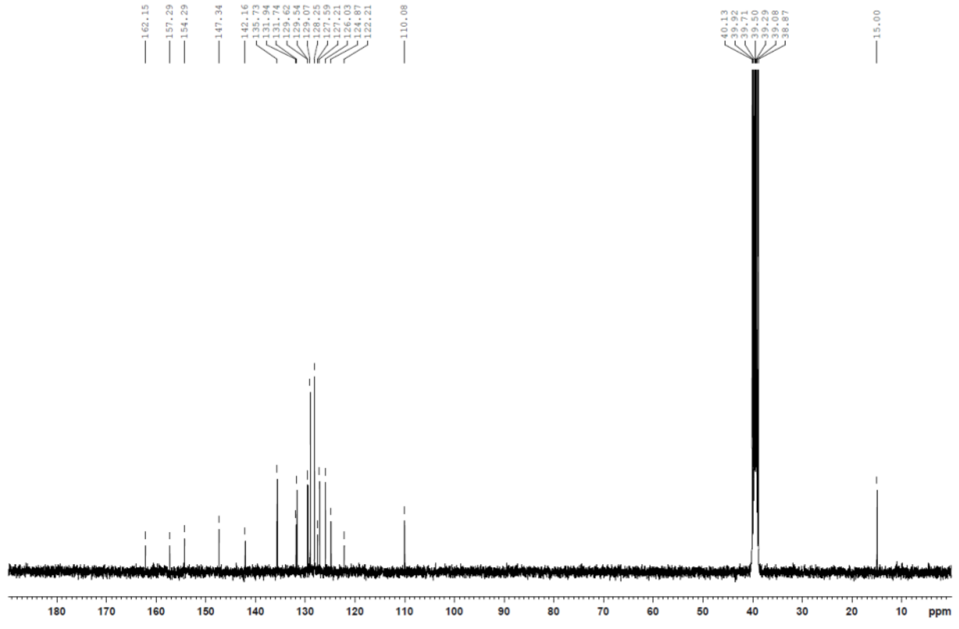

Aromatic expansion of ^1H-^{13}C bidimensional correlations - HSQC spectrum of compound **7f**

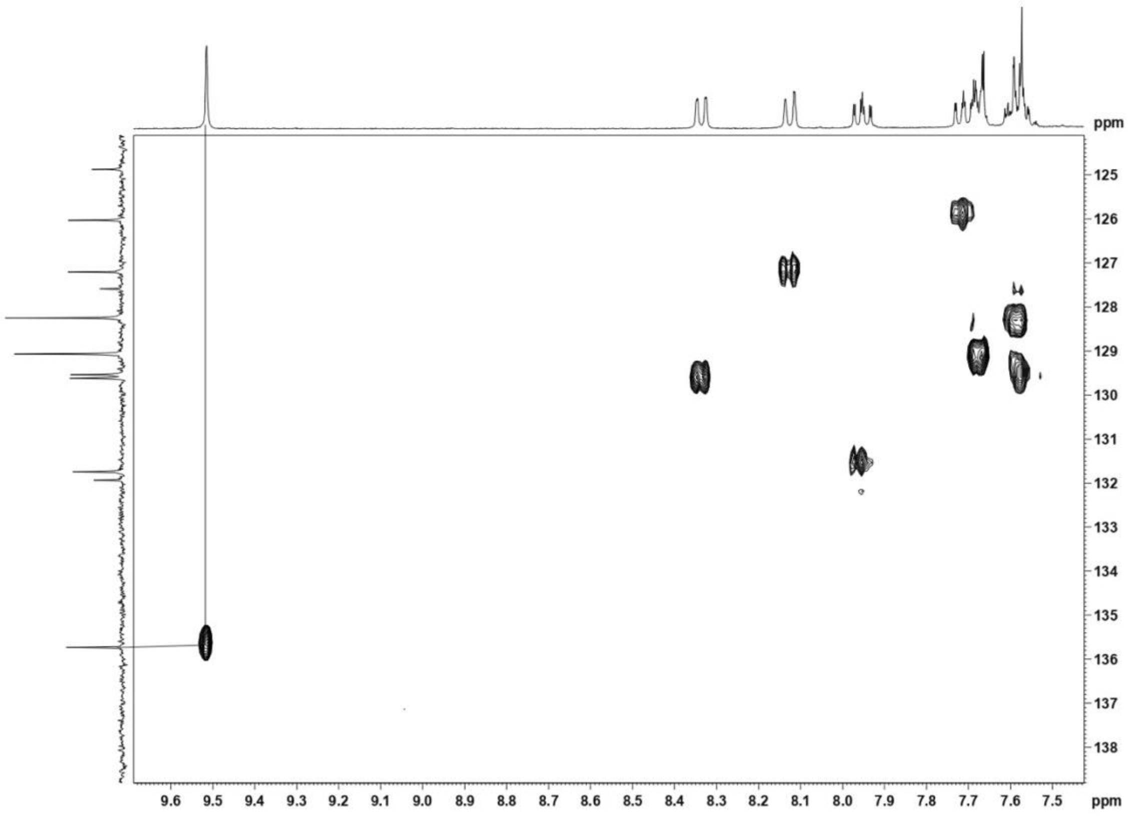

nOe between the methyl group and the 5-H of compound **7f**

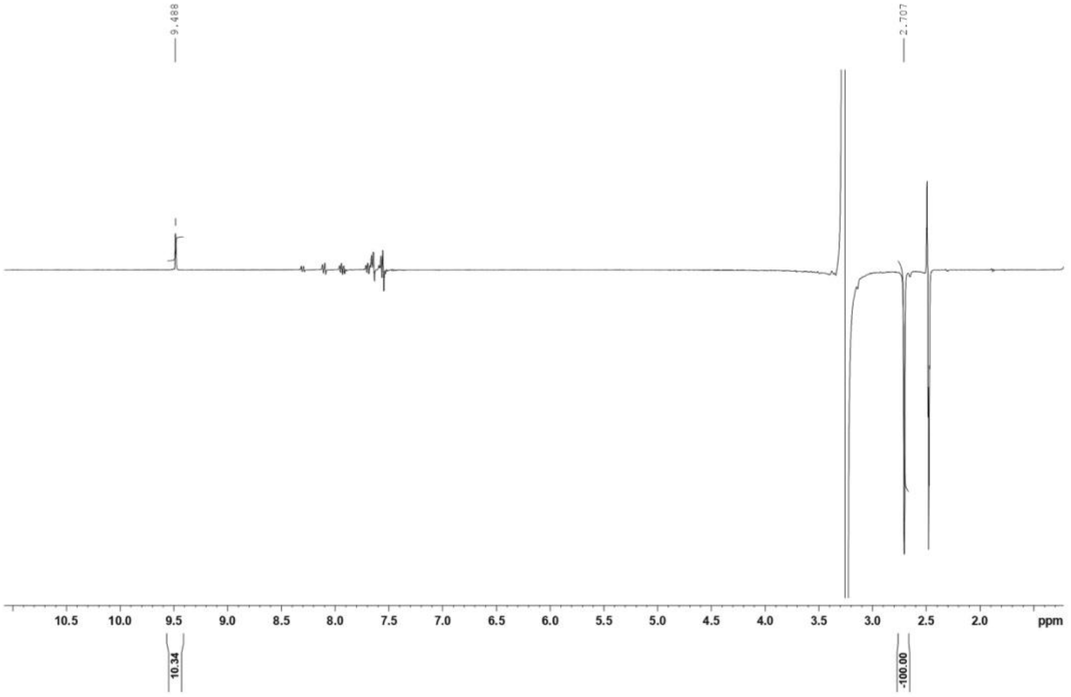

Supplement: Supplementary file 1 [file molecules-30-01999-s001.zip › molecules-3532260-supplementary.pdf]
